# The Health Inequality Impact of Liquid Biopsy to Inform First-Line Treatment of Advanced Non–Small Cell Lung Cancer: A Distributional Cost-Effectiveness Analysis

**DOI:** 10.1016/j.jval.2023.08.010

**Published:** 2023-09-22

**Authors:** Jeroen P. Jansen, Meera V. Ragavan, Cheng Chen, Michael P. Douglas, Kathryn A. Phillips

**Affiliations:** Department of Clinical Pharmacy, UCSF Center for Translational and Policy Research on Precision Medicine (TRANSPERS), San Francisco, CA, USA (Jansen, Chen, Douglas, Phillips); UCSF Helen Diller Family Comprehensive Cancer Center, San Francisco, CA, USA (Jansen, Phillips); UCSF Philip R. Lee Institute for Health Policy, San Francisco, CA, USA (Jansen, Phillips); Division of Hematology and Oncology, UCSF Department of Medicine, San Francisco, CA, USA (Ragavan).

**Keywords:** distributional cost-effectiveness analysis, health equity, health inequality impact, liquid biopsy, non-small cell lung cancer

## Abstract

**Objectives:**

To perform a distributional cost-effectiveness analysis of liquid biopsy (LB) followed by, if needed, tissue biopsy (TB) (LB-first strategy) relative to a TB-only strategy to inform first-line treatment of advanced non–small cell lung cancer (aNSCLC) from a US payer perspective by which we quantify the impact of LB-first on population health inequality according to race and ethnicity.

**Methods:**

With a health economic model, quality-adjusted life-years (QALYs) and costs per patient were estimated for each subgroup. Given the lifetime risk of aNSCLC, and assuming equally distributed opportunity costs, the incremental net health benefits of LB-first were calculated, which were used to estimate general population quality-adjusted life expectancy at birth (QALE) by race and ethnicity with and without LB-first. The degree of QALYs and QALE differences with the strategies was expressed with inequality indices. Their differences were defined as the inequality impact of LB-first.

**Results:**

LB-first resulted in an additional 0.21 (95% uncertainty interval: 0.07–0.39) QALYs among treated patients, with the greatest gain observed among Asian patients (0.31 QALYs [0.09–0.61]). LB-first resulted in an increase in relative inequality in QALYs among patients, but a minor decrease in relative inequality in QALE.

**Conclusions:**

LB-first to inform first-line aNSCLC therapy can improve health outcomes. With current diagnostic performance, the benefit is the greatest among Asian patients, thereby potentially widening racial and ethnic differences in survival among patients with aNSCLC. Assuming equally distributed opportunity costs and access, LB-first does not worsen and, in fact, may reduce inequality in general population health according to race and ethnicity.

## Introduction

Lung cancer is a major cause of cancer-related deaths, and the most common type (85%) is non–small cell lung cancer (NSCLC).^1,2^ Treatment of patients with advanced-stage NSCLC (aNSCLC), ie, stage IIIB or IV, who harbor EGFR, BRAF, MET, RET, NTRK, KRASG12C, ALK, or ROS1 alterations with targeted therapies has improved survival.^3–5^ According to guidelines, patients with aNSCLC should undergo broad genomic profiling with next-generation sequencing (NGS) to inform treatment decisions.^6,7^

Although tissue biopsy (TB)-based NGS is the gold standard for identifying driver mutations in the diagnostic workup of aNSCLC, circulating tumor DNA liquid biopsy (LB) is an emerging technology that can be used when TB may not be feasible or tissue quantity is insufficient for comprehensive NGS (the latter affecting up to 30% of patients).^8–10^ LB has the benefits of a shorter turnaround time (TAT) allowing for faster initiation of first-line therapy and avoidance of the potential for complications associated with an invasive procedure.^11^ However, given the variable sensitivity and the inability to determine PD-L1 expression, current guidelines do not support LB use in isolation if TB is feasible; follow-up TB NGS should be planned when an oncogenic driver is not identified.^6,7^

Adib et al found that the prevalence of targetable genomic alterations in NSCLC varies according to genetic ancestry.^12^ This suggests that with the currently available targeted therapies, patients with aNSCLC with different genetic ancestry may benefit from treatment to a different degree. Several studies have shown that the sensitivity of LB-based molecular profiling varies by oncogenic driver mutation (specificity is close to 100%).^13–15^ These two factors combined may result in a differential impact of LB to identify oncogenic driver mutations, inform first-line therapy of aNSCLC, and ultimately survival across subgroups according to genetic ancestry or self-reported race. Because aNSCLC is characterized by disparities in incidence and survival across race and ethnicity, with the greatest incidence and worst survival among non-Hispanic (NH)-Black individuals, understanding the distributional effects of LB is very relevant from a health equity perspective.^16^

Englmeier et al showed that it is likely cost-effective to add LB in the diagnostic workup of aNSCLC to inform first-line therapy.^17^ Cost-effectiveness analysis (CEA) as a tool to quantify the value of a health technology focuses on improvement in total health but ignores distributional effects across demographic subgroups. With a renewed and keener scrutiny of health equity issues, it is important to also determine whether a new health technology will reduce or perpetuate inequalities in health outcomes, as part of value assessment.^18,19^ Such evaluations can be achieved through a distributional CEA (DCEA), an extension of conventional CEA.^18,20–24^ When the impacts of the new intervention on average health and health inequality are opposed, a trade-off analysis can help decide whether the new technology is preferred over standard of care.^20,24^

The objective of the current study was to perform a DCEA of LB to inform first-line treatment of aNSCLC to quantify its health inequality impact. The target population was patients with aNSCLC with pathologic confirmation but with insufficient tissue for molecular testing. Equity-relevant subgroups of interest were defined according to race and ethnicity. The comparison of interest was LB followed by TB if the LB is negative (LB-first strategy) or directly repeat a TB without LB (TB-only strategy). The evaluation was performed from a US health system payer perspective. We quantified the health inequality impact of the LB-first strategy in the target patient population as the difference in the inequality of the health consequences versus the LB-only strategy, as well as the impact of LB-first on the health distribution at a general population level factoring opportunity costs and lifetime risk of aNSCLC. We are careful to distinguish the terms health disparity and inequality. Health “disparity” or “inequity” is defined as a particular type of health difference between individuals or groups that is unfair and caused by social or economic disadvantage.^25,26^ We use the term “inequality” simply to measure and express how dissimilar the health outcomes are between subgroups without making a judgment or assessment to which degree these inequalities are caused by social or economic disadvantage.^26^

## Methods

Employing a health economic model, the expected quality-adjusted life-years (QALYs) and costs were estimated with the LB-first and TB-only strategies followed by test-result-informed first-line therapy for NH-White, NH-Black, Asian, and Hispanic aNSCLC patients for a remaining lifetime horizon. Given the lifetime risk of aNSCLC with insufficient tissue for molecular testing, and assuming health opportunity costs are equally divided within the general population, the incremental net health benefits (iNHBs) of LB-first at the general population level were calculated. Adding the iNHB to reference quality-adjusted life expectancy at birth (QALE) values by race and ethnicity, we got QALE estimates when TB-only is replaced by LB-first. The change in the degree of QALYs and QALE differences with LB-first relative to TB-only was defined as the inequality impact of LB-first in the target patient population and general population, respectively. (In the online Supplemental Materials found at https://doi.org/10.1016/j.jval.2023.08.010, a detailed overview of the process of estimating health inequality impact in the target patient population and general population is provided.)

### Model Structure

The model consisted of a tree structure reflecting the possible test outcomes with the LB-first and TB-only strategy (see Appendix Fig. 1 in Supplemental Materials found at https://doi.org/10.1016/j.jval.2023.08.010) and expected progression-free survival (PFS) and overall survival (OS) for each test-informed first-line treatment obtained with a partitioned survival modeling (PSM) approach.

With LB we get a true- or false-positive test result for the presence of an EGFR, BRAF, MET, RET, NTRK, KRAS, ALK, or ROS1 alteration. Upon a negative LB test result, a follow-up TB is performed.^6,7^ This follow-up test will show a true or false-positive result for the presence of one of the driver mutations as well. If no mutation is identified (either true or false negative), treatment is based on whether the follow-up test shows a true or false-positive result for PD-L1 expression classified into a tumor proportion score (TPS) 1% to 49% or TPS 50%, ≥ or a true- or false-negative PD-L1 expression.

In the absence of LB, a tissue rebiopsy is performed that may show a true or false-positive targetable mutation. If no targetable driver mutation is identified (either true or false negative), treatment is based on a true or false-positive PD-L1 TPS 1% to 49%, TPS ≥50%, or true- or false-negative PD-L1 expression.

The following structural assumptions were made when estimating PFS and OS curves associated with each test outcome: (1) With a true positive or true negative test result, PFS and OS with matching first-line recommended treatment, as observed in routine practice, is expected. (2) Suboptimal treatment is provided with a false-positive or false-negative test result, resulting in worse PFS and OS, expressed with treatment and mutation-specific hazard ratios (HRs). (3) With a rebiopsy required for a TB-based NGS, first-line treatment initiation is delayed relative to when a positive LB result is obtained, thereby increasing the hazard of disease progression and mortality. Accordingly, each final branch of the decision tree was “linked” with a specific PFS and OS curve.

### Model Outcomes and Quantifying Health Inequality Impact

Expected QALYs, costs associated with the diagnostic workup (ie, determining the presence of mutations or PD-L1 expression), and total costs (diagnostic workup and treatment and disease management) over the model’s time horizon, all discounted at 3% per year,^27^ were estimated for each subgroup by “folding back the tree,” given its conditional probabilities, costs associated with each test performed, as defined by the decision tree, and QALYs and costs associated with treatment from the PSMs for each final branch of the tree. Expected NHBs (without and with treatment and disease management costs) with LB-first and TB-only expressed per member of the general population by race and ethnicity were calculated, assuming that health opportunity costs are equally distributed across the general population according to the following^28^:

$${\text{NHB}}_{\text{race\_ethnicity}}{\text{=incidence}}_{\text{race\_ethnicity}}\times {\text{QALY}}_{\text{race\_ethnicity}}\;\;-\sum {\text{proportion\_general\_population}}_{\text{race\_ethnicity}}\;\times {\text{incidence}}_{\text{race\_ethnicity}}{\text{×cost}}_{\text{race\_ethnicity}}\text{/opportunity cost threshold}.$$

(Actual equations are provided in the online Supplemental Materials found at https://doi.org/10.1016/j.jval.2023.08.010.) Subsequently, the iNHB with LB-first relative to TB-only were calculated per member of the general population by race and ethnicity. Applying the iNHBs to a reference distribution of health of the general population measured in QALE by race and ethnicity, which we call the “pre-LB-first” distribution, we obtained a “post LB-first” distribution of QALEs.

With the Atkinson inequality index (between 0 and 1), which works on a relative scale, and the Kolm inequality index (≥0), which measures dissimilarity on an absolute scale, we quantified the inequality in QALYs in the target patient population across race and ethnicity with LB-first and TB-only for different levels of social preference for reducing health inequalities (set with the inequality-aversion parameter).^29,30^ Smaller values of the Kolm and Atkinson indices indicate lower levels of inequality. The difference in the degree of inequality between these two strategies was defined as the health inequality impact of LB-first in the target patient population. The inequality of the “pre-LB-first” and “post LB-first” QALE distributions were also expressed with the Atkinson and Kolm inequality indices and used to quantify the health inequality impact of LB-first for the general population. A reduction in the degree of inequality with LB-first was defined as a positive (ie, favorable) health inequality impact. (Equations are provided in the online Supplemental Materials found at https://doi.org/10.1016/j.jval.2023.08.010l.)

When the impacts of LB-first on average health and health inequality are opposed, a trade-off analysis can help decide whether LB-first is preferred over TB-only by combining average QALY or QALE gain and inequality improvement in QALY or QALE in a single social welfare index: equally distributed equivalent (EDE) QALYs or QALEs (QALY_EDE_, QALE_EDE_); (equations are provided in the online Supplemental Materials found at https://doi.org/10.1016/j.jval.2023.08.010l ).^31^ The EDE is the level of health (expressed in QALYs or QALE) that, if provided uniformly across race and ethnicity, would yield the same amount of welfare to the actual distribution of health across race and ethnicity.

### Model Input Parameters and Source Data

Model input parameters and estimates are listed in Table 1^6,10,12,14,32–61^. The core elements of the model to estimate distributional effects with LB-first were as follows: proportion of NH-White, NH-Black, Asian, and Hispanic in the general population,^32^ baseline QALE,^33,34^ lifetime risk of aNSCLC (obtained by multiplying life expectancy at birth with age-standardized incidence rates^35^) by race and ethnicity, proportion of aNSCLC patients with insufficient tissue for NGS,^10^ prevalence of driver mutations^12^ and PD-L1 expression by race and ethnicity,^36–38^ diagnostic test performance (ie, true/false positive/negative rates) by driver mutation with LB- and TB-based NGS,^14,39^ and PFS and OS by driver mutation with matched and unmatched therapy.^40–52^

PFS and OS with the different therapies were obtained from real-world studies that provided Kaplan-Meier (KM) curves. If not available, clinical trials were used. Published KM curves were digitized and pseudo-individual patient time-to-event data (IPD) created according to Guyot et al to facilitate fitting Weibull survival models.^62^ Scale and shape parameters (estimated with R flexsurv) were used as input for the PSM part of the simulation model. PFS and OS with mismatched treatment as a result of a false test results were adjusted with HRs obtained from a large study of mutation-treatment interactions using real-world clinicogenomics data.^52^ The impact of faster time to treatment with LB relative to TB because of a shorter TAT on PFS and OS was estimated based on an analysis of digitized OS KM curves provided for immunotherapy (IO) and best supportive care (BSC) by Shokoohi et al.^54^ With 1.5-week TAT with LB and a 4.5-week TAT with TB, as reported by Raez et al,^55^ we assumed that during TAT patients experience mortality according to the BSC KM curve and thereafter mortality according to the IO curve. By generating pseudo-IPD for these two “BSC-followed-by-therapy” curves, an HR was estimated for the 1.5-week versus 4.5-week TAT, corresponding to a 3-week earlier time to treatment with LB.^62^ We assumed this estimate applied to all treatments. This HR was also transformed into a 1-week-faster time-to-treatment initiation with LB relative to TB assuming a log-linear relationship with time (see Appendix Fig. 2 in Supplemental Materials found at https://doi.org/10.1016/j.jval.2023.08.010). To capture differences in prognostic factors of NSCLC survival across the race and ethnicity subgroups beyond differences in the distribution of oncogenic driver mutations (ie, survival disparities), we calibrated the modeled OS in the absence of LB to match subgroup-specific Surveillance, Epidemiology, and End Results aNSCLC mortality estimates.^53^ In the online Supplemental Materials, the actual PFS and (extrapolated) OS curves “linked” to each final branch of the decision tree comparing LB-first and TB-only are presented (Appendix Figs. 3-28 in Supplemental Materials found at https://doi.org/10.1016/j.jval.2023.08.010).

Duration of first-line treatment was modeled according to Food and Drug Administration labeling information. Because we did not explicitly model second-line PFS, the duration of second-line treatment was assumed based on a ratio of real-world second-line PFS and OS as reported by Marmarelis et al and Bains et al^60,61^ We assumed that half of the patients opt for BSC upon first-line progression.^63,64^ No drug therapy beyond second-line was included. We used 2022 Federal Supply Schedule drug costs for targeted and nontargeted therapies.^65^ More information about annual drug costs is provided in Appendix Tables 1 and 2 in Supplemental Materials found at https://doi.org/10.1016/j.jval.2023.08.010. Healthcare resources for disease management in the pre- and post-progression states were based on Stargardter et al^58^ (see Appendix Tables 3 and 4 in Supplemental Materials found at https://doi.org/10.1016/j.jval.2023.08.010). The frequency of healthcare resource use was assumed the same for all therapies and subgroups. Costs of NGS with LB and TB were based on a prior study.^57^ Separate estimates were used for Medicare and commercial payer perspectives. Costs from a Medicare perspective associated with intravenous drug administration and disease management were calculated based on the resource use^58^ multiplied by unit cost obtained from the Centers for Medicare & Medicaid Services 2022 fee schedule.^66^ Costs from a commercial payer perspective were based on a prior study.^58^ Where needed, costs were inflated to 2022 US dollars based on the medical care component of the Consumer Price Index.^67^ Costs were calculated based on a blended NSCLC population where 67% are Medicare patients and the rest is commercially insured.^59^ Costs for managing treatment-related adverse events were not included because of the relatively limited contribution to overall costs.

Health utility (to estimate QALYs) was assumed only to be affected by time in the pre- and post-progression states^56^; we did not assume disutility associated with TB, potential complications, or treatment-related adverse events.

### Model Analyses

The model was developed and analyses were performed with the hesim package in R.^68^ Uncertainty in input parameters was expressed with appropriate probability distributions (Table 1) and propagated through the model with 2nd-order Monte Carlo simulation. Simulation results of model outcomes were summarized with the mean and 2.5th and 97.5th percentiles to reflect a 95% uncertainty interval. A base-case analysis was performed without costs associated with aNSCLC treatment and disease management, using a 3-week treatment delay with TB relative to LB, an opportunity cost threshold of $150 000 per QALY, and an Atkinson and Kolm inequality index of, respectively, 11 and 0.15.^31,34,69^ Additional analyses were performed incorporating costs associated with treatment and disease management: using a 1-week difference in TAT between LB and TB, different opportunity cost thresholds ($50 000, $100 000, and $200 000/QALY), and different degrees of inequality aversion (Atkinson 0–15; Kolm 0–0.3).

## Results

**Figure 1.**
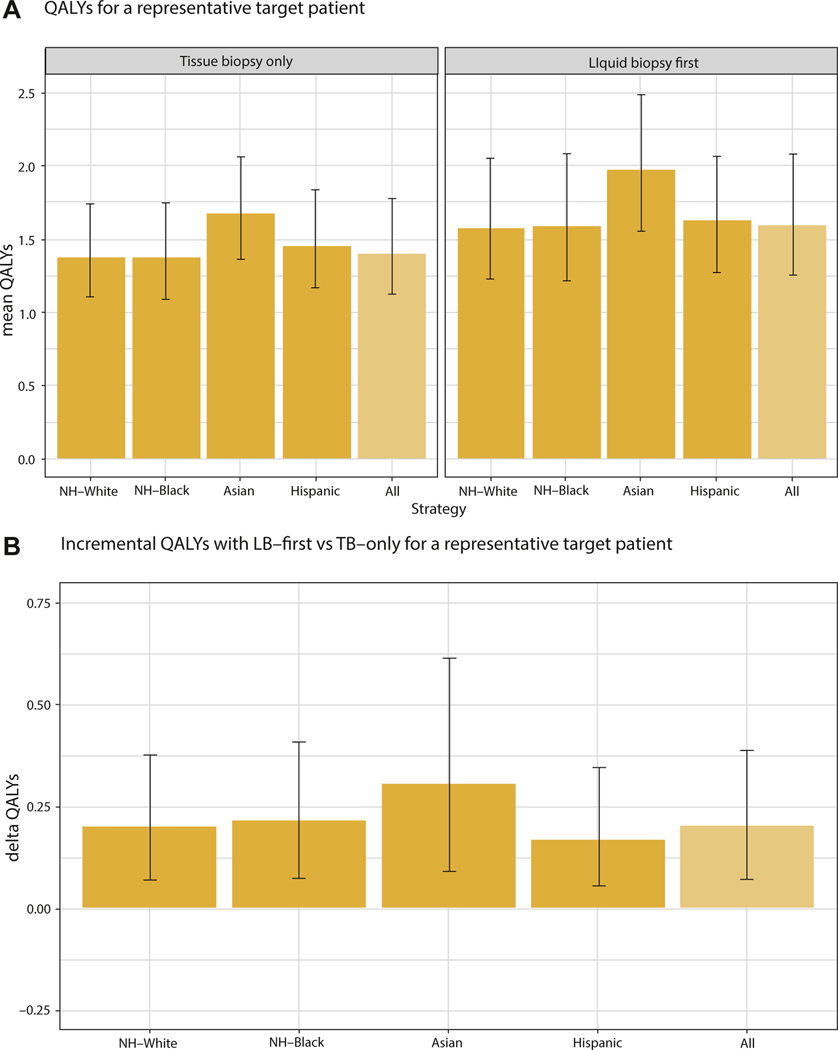

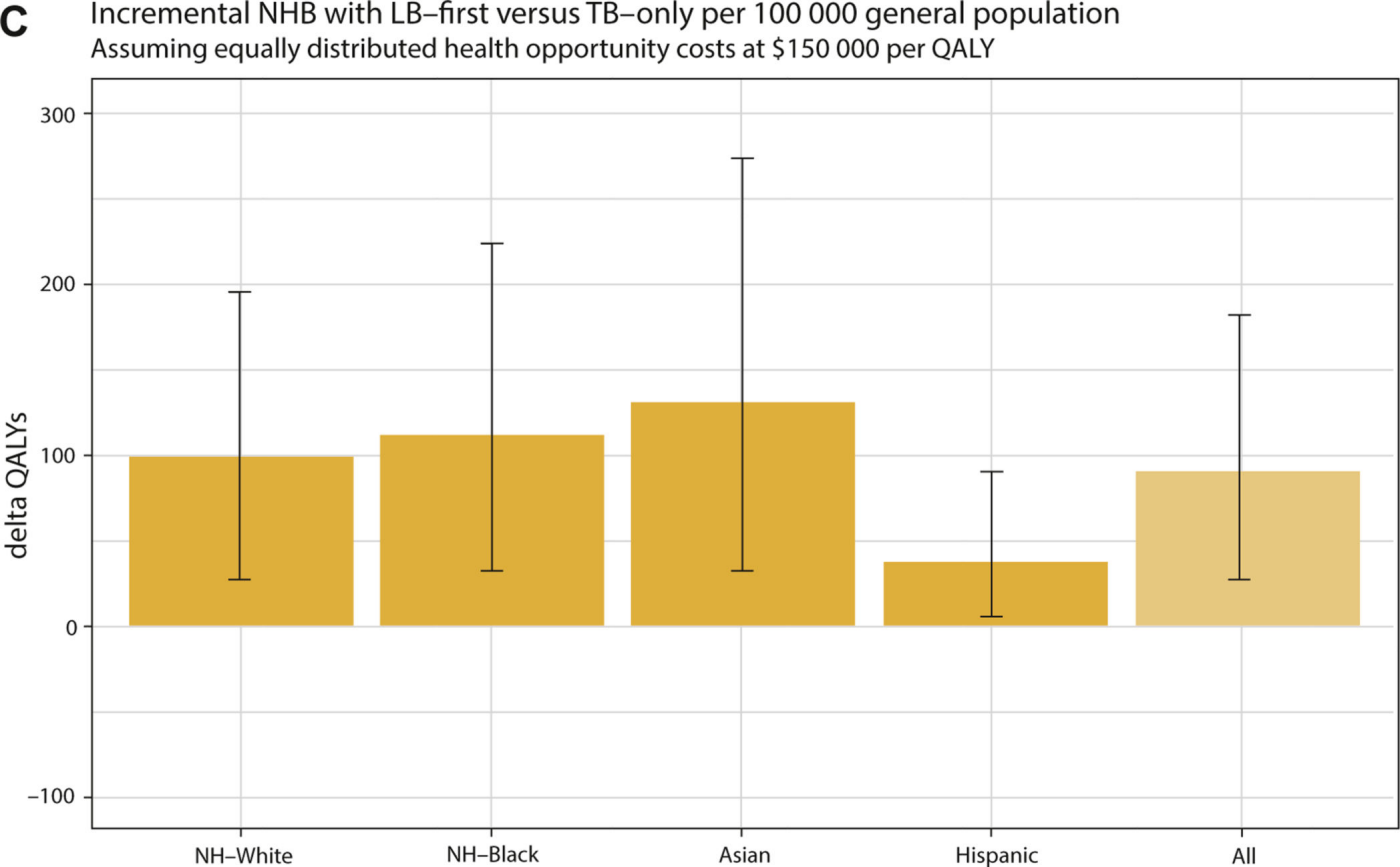
Expected discounted QALYs per target patient (A); incremental QALYs per target patient (B); and iNHB per 100 000 individuals of the general population based on the lifetime risk of aNSCLC without sufficient tissue for molecular testing (NH-White 1.81% × 30%, NH-Black 1.92% × 30%, Asian 1.54% × 30%, and Hispanic 0.95% × 30%) and factoring in equally distributed opportunity costs related to diagnostic workup at a threshold of $150 000 by race and ethnicity (C). aNSCLC indicates advanced non–small cell lung cancer; iNHB, incremental net health benefit; LB, liquid biopsy; NH, non-Hispanic; QALY, quality-adjusted life-year; TB, tissue biopsy.

In Figure 1 the expected QALYs per target patient with TB-only and LB-first are presented (Fig. 1A), the incremental QALYs per target patient with LB-first (Fig. 1B), and the iNHB (incorporating equally distributed health opportunity costs related to diagnostic workup) per 100 000 individuals of the general population with LB-first (Fig. 1C). In the base-case analysis, LB-first resulted in an additional 0.21 QALYs per patient relative to TB-only (Table 2). The greatest gain was observed among Asian patients (0.31 QALYs). LB-first was associated with greater costs related to diagnostic workup than TB-only (+$3270). The iNHB with LB-first relative to TB-only, which reflects the general population net health gains, was 91 QALYs per 100 000 individuals of the general population, indicating LB-first is cost-effective at $150 000/QALY. The iNHB was almost 4 times as large for Asian than Hispanic individuals. Applying these iNHB estimates to the baseline QALE estimates, we get the “post LB-first” general population QALE estimates, as presented in Table 2.

Table 3 shows the inequality metrics. The relative inequality in QALYs in the target patient population was greater with LB-first (0.01291 at an Atkinson inequality-aversion value of 11) than with TB-only (0.01109); LB-first resulted in a 20% (95% uncertainty interval −14% to 93%) increase in relative inequality. In terms of absolute inequality (Kolm), the increase was 76% (4%,−214%). The relative inequality in QALE for the general population showed a very minor decrease with LB-first; the change in inequality was −0.014% (−0.03% to −0.003%). Similar results were obtained in terms of absolute inequality.

**Figure 2.**
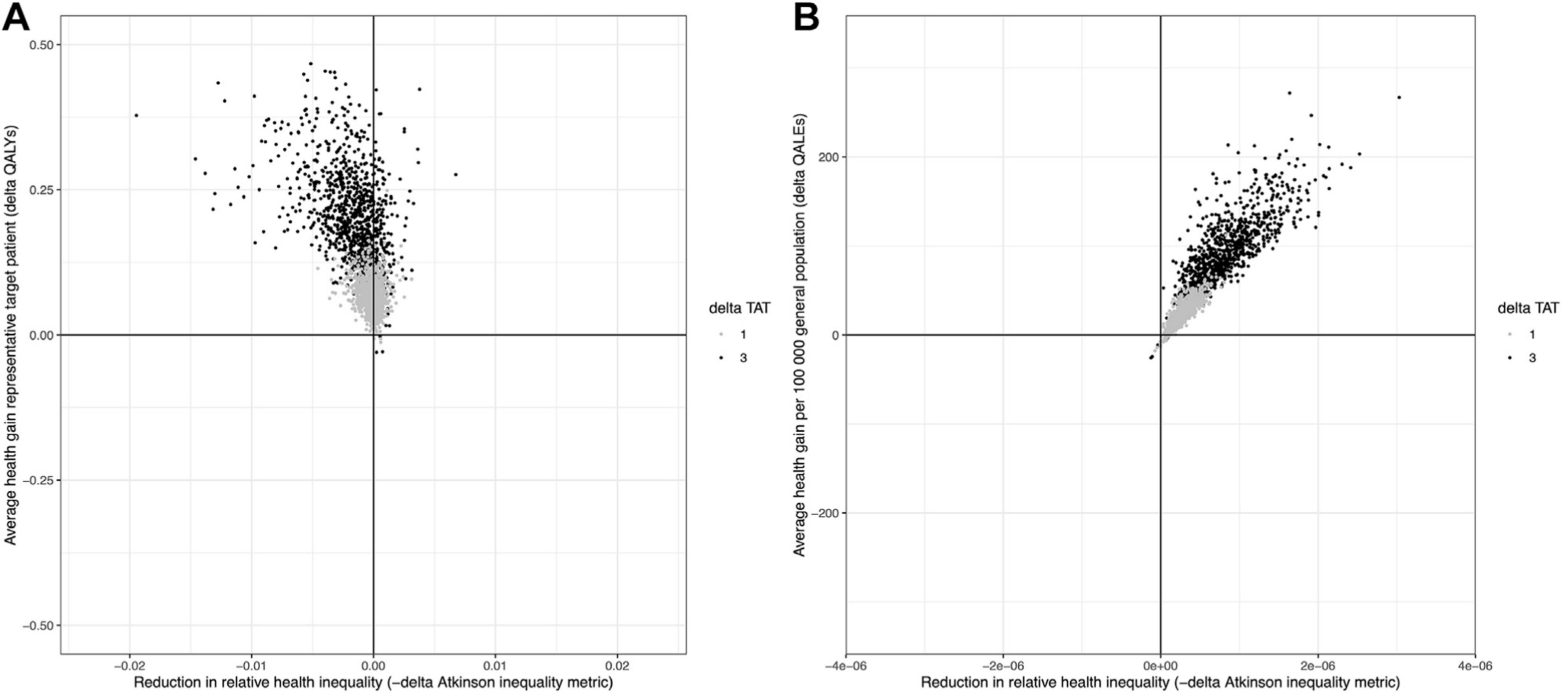
The joint uncertainty distribution of the reduction in the relative inequality in QALYs in the target patient population (A) or QALE in the general population (B) and average gain in QALYs per target patient and QALE per 100 000 individuals of the general population with the LB-first strategy is presented assuming an opportunity cost threshold of $150 000 for the base-case scenario assuming 3 weeks faster treatment initiation with LB (black dots) and the alternative scenario assuming a 1-week faster treatment initiation with LB (gray dots). Each dot reflects a model simulation result with a different set of input parameters sampled from the model input distributions. LB indicates liquid biopsy; QALE, quality-adjusted life expectancy at birth; QALY, quality-adjusted life-year; TAT, turnaround time.

In Figure 2, the average health gain is presented against the corresponding improvement (ie, reduction) in inequality for the target patient population in terms of QALYs (Fig. 2A) and the general population in terms of QALE (Fig. 2B) for each of the 2nd order Monte Carlo simulations. In the base-case (black dots), there is a >95% probability that LB-first results in an increase in average QALYs combined with an increase in inequality in QALY in the target population (ie, >95% of the simulation results fall in the upper-left quadrant of Fig. 2A). There is >99% probability that LB-first results in greater health outcomes without an increase in inequality in health outcomes in the general population.

**Figure 3.**
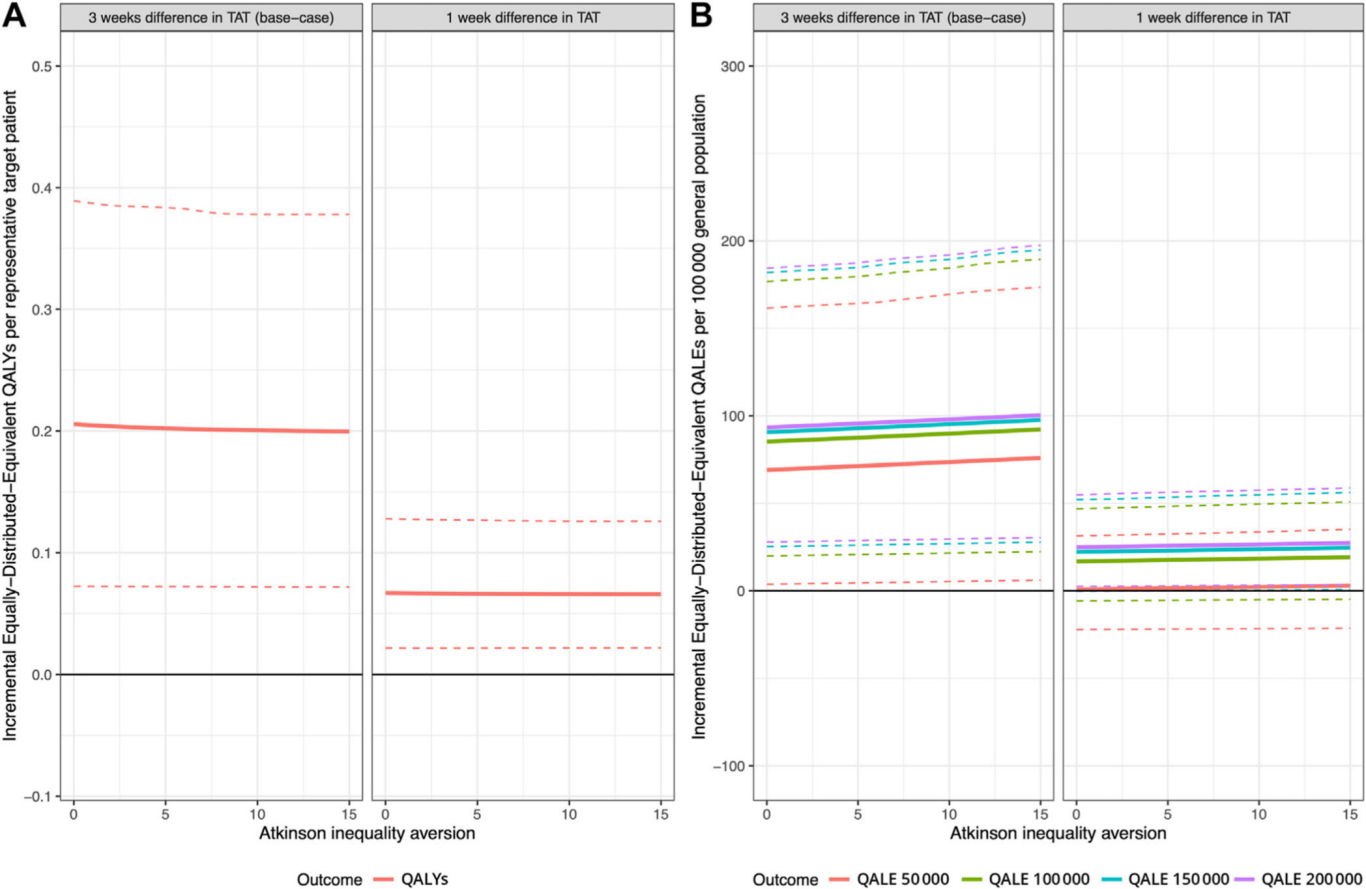
Incremental equally distributed equivalent QALYs and QALE with the LB-first strategy for different degrees of relative inequality aversion, opportunity cost thresholds, and assuming a 3-week or 1-week faster treatment initiation with LB. Dashed lines reflect 95% uncertainty interval of the estimates. LB indicates liquid biopsy; QALE, quality-adjusted life expectancy at birth; QALY, quality-adjusted life-year; TAT, turnaround time.

The Atkinson-based incremental QALY_EDE_ and QALE_EDE_ are presented in Figure 3. For the base-case (3-week-faster TAT with LB) the 95% uncertainty intervals exclude the null and estimates hardly changed as a function of the degree of relative inequality aversion in both the target patient population (Fig. 3A) and general population (Fig. 3B). Hence, the LB-first strategy is preferred over the TB-only strategy when both average health gains and inequality impact are considered.

When costs related to subsequent treatment and management were incorporated in the evaluation, the iNHB with LB-first was negative at a threshold of $150 000/QALY because the annual treatment and disease management costs are >$150 000 per year (see Appendix Table 5 in Supplemental Materials found at https://doi.org/10.1016/j.jval.2023.08.010). Accordingly, the positive QALY gain with LB-first relative to TB-only per patient resulted in a QALE loss at a general population level when opportunity costs were included. The inequality in corresponding QALE estimates did not increase (see Appendix Table 6 in Supplemental Materials found at https://doi.org/10.1016/j.jval.2023.08.010).

The impact of the opportunity cost threshold on the iNHB and health inequality impact estimates for different degrees of inequality aversion are presented in the online supplement (Appendix Tables 7-9 in Supplemental Materials found at https://doi.org/10.1016/j.jval.2023.08.010). The relative inequality estimates (Atkinson) barely increased for greater opportunity cost thresholds, and the impact in terms of absolute inequality (Kolm) was independent of the opportunity cost threshold because of the assumption of equally distributed opportunity costs.

When the TAT difference between LB and TB was just 1 week, the direction of QALY and QALE gains and inequality impact were the same, but the magnitude smaller (Appendix Tables 10 and 11 in Supplemental Materials found at https://doi.org/10.1016/j.jval.2023.08.010; Fig. 2, gray dots). The 95% intervals of the incremental QALE_EDE_ only excluded no difference for an opportunity cost threshold of $200 000 (Fig. 3).

## Discussion

In this DCEA, an LB-first strategy to inform first-line aNSCLC therapy resulted in QALY gains along with an increase in the differences in QALYs between patients of different race and ethnicity relative to a TB-only strategy. The greatest gains were estimated for Asian patients. Given the opposite impact of LB on average health gain and health inequality in the target patient population, a trade-off analysis was performed for the effect in the target patient population, which indicated that the LB strategy remained beneficial across a range of values for inequality aversion. Given the lifetime risk of aNSCLC (with insufficient tissue for molecular testing) and assuming equally distributed health opportunity costs related to diagnostic workup resulted in a positive iNHB, indicating the cost-effectiveness of LB-first, and a (minor) reduction in general population health inequality. To clarify these findings, LB-first (slightly) reduces general population health inequality because it delivers larger general population health gains for less healthy race and ethnicity subgroups among the vast majority of the general population. Specifically, a larger gain for the NH-Black general population subgroup than the better off NH-White group (ie, greater “pre-LB-first” QALE), and a larger gain for the NH-White group than the better off Hispanic group. The largest general population health gain goes to the healthiest group, ie, the Asian group, who only make up about 6% of the US general population. Because the Atkinson and Kolm summary indices of inequality embody the (reasonable) value judgment that an inequality reduction among the vast majority of the population outweighs an inequality increase for a relatively small group at the top of the health distribution, we yield an overall reduction in general population health inequality.

When costs involving treatment for NSCLC were included in addition to the costs related to diagnostic workup, an LB-first approach resulted in a negative iNHB. However, the interpretation of this should factor in the expensive nature of first-line aNSCLC treatment regardless of whether LB or TB is used; guideline-compliant first-line drug therapies cost beyond $150 000 per year in the United States.

In line with guidelines, we assumed that, after a negative LB test result, a repeat biopsy and TB test is performed, which is considered the gold standard.^6,7^ As such, false-negative LB results will be identified with a TB. With false-positive driver mutation findings negligible, the clinical utility of LB as characterized by our model depended on the following: (1) the sensitivity of LB, (2) how much faster treatment can be initiated because of the shorter TAT with LB than TB, and (3) whether delayed treatment hurts PFS and OS. Accordingly, heterogeneity in expected PFS and OS between race and ethnicity subgroups was the result of the different distributions of driver mutations in each subgroup and the LB test performance that varies by driver mutation. EGFR is the driver mutation that varies the most between race and ethnicity and most prevalent among Asian individuals. Furthermore, LB exhibits greater sensitivity for detecting EGFR mutations compared with most other mutations. These factors together explain the greatest QALY gains estimated with LB-first in the Asian subgroup.

The impact of a faster TAT on PFS and OS is the key factor in the model. We inferred and used an HR associated with a 3-week faster treatment initiation of 0.72 (0.56–0.93) based on data reported by Shokoohi et al.^54^ Most published studies that evaluated the impact of earlier versus delayed treatment were either prone to “immortal time” bias or not applicable because they concerned starting treatment before test results were available.^70,71^ Sheinson et al reported that >3-week-faster ALK targeted treatment is associated with an OS HR of about 0.5, which is a larger impact than we used.^72^

Madison et al compared PFS among patients on matched first-line therapies after LB or TB using data from the Flatiron Health-Foundation Medicine Clinico-Genomic Database and found a PFS HR of 0.68 (95% CI 0.36–1.26) favoring LB.^73^ Our model estimated the difference in PFS between LB-first and TB-only at 1.7 months, corresponding roughly to an average PFS HR of 0.85. This is similar to Madison et al, thereby validating our model output estimates and the used TAT impact parameter.

The current DCEA has some limitations. We defined subgroups solely based on race and ethnicity, but a more-complete health inequality impact evaluation should also consider age, socioeconomic status, geographic location, or a combination of these factors.^74^ Differential access to a new health technology is an important factor when estimating its health inequality impact. Frequently, disadvantaged individuals have reduced access to new health technology. In the case of diagnostic workup to inform first-line aNSCLC treatment, it can be argued that LB provides easier access to a complete molecular assessment for patients in geographically remote areas, thereby contributing to a positive equity impact.^75–78^ The assumption of equally distributed opportunity costs in this study is convenient and arguably conservative but may not be realistic. Although determining appropriate distributions is challenging, an attempt should be made.^28^ In this study, we focused on 1 specific clinical scenario (insufficient tissue material to perform NGS), but other clinical scenarios for LB (eg, when a patient is unfit for a biopsy or monitoring disease progression) are worthy of evaluation as well.

We quantified the health inequality impact of LB-first in 2 ways: (1) as the difference in inequality of the health consequences expressed as QALYs between LB-first and TB-only in the target patient population and (2) as the difference in inequality of the “post LB-first” and “pre-LB-first” QALE distributions in the general population. This second approach factors in health opportunity costs across society and the occurrence of the condition for which the new technology is indicated across subpopulations. This second approach is in line with recommendations for DCEA.^20^ We opted for the first approach as well because we believe that understanding the distributional effect of a new intervention on outcomes in the target patient population is very relevant information; the two sets of analyses provide complementary information and ensure that a complete picture of the impact of the technology of interest is obtained.

The findings of this article bring up an interesting policy question. How would payers value a new technology when it has a small impact on increasing inequality in patient outcomes combined with a small positive impact on reducing general population health inequality? Are they willing to pay a bit less because it increases inequality in patient outcomes or a bit more because it reduces general population health inequality? The answer depends on their primary policy goal: reducing inequality in treatment outcomes or reducing general population health inequality (with inequality in treatment outcomes only matters insofar as it contributes to this broader goal). A future study to better understand preferences in this regard would be interesting.

In conclusion, given the evidence available and the assumptions of our modeling study, LB-first to inform first-line aNSCLC therapy can result in improved health outcomes for patients. With current diagnostic performance for different driver mutations, the benefit of LB-first is likely the greatest among Asian patients, thereby potentially widening existing differences in survival between patients with aNSCLC of different race and ethnicity. An improvement in LB test sensitivity is wanted to avoid this. Assuming equally distributed opportunity costs and access, LB-first for aNSCLC does not worsen and, in fact, may reduce inequality in general population health according to race and ethnicity.

## Supplementary Material

Supplement

## Figures and Tables

**Table 1.** Model input parameters.

Parameter		Estimates	NH-Black	Asian	Hispanic		Distribution	Source	Comment
		NH-White							
Distribution race and ethnicity in US		60.6%	13.9%	6.2%	19.3%		Fixed	US Census (2022)^32^	
Baseline quality-adjusted life expectancy (QALE) general population		68.798	65.446	74.878	71.762		Fixed	KFF (2023)^33^; Kowal et al (2022)^34^	Kowal estimates adjusted according to KFF life expectancy data by race and ethnicity
Standardized incidence rate aNSCLC (events per 100 000 per year)		23.0	25.7	18.0	11.6		Fixed	SEER^35^	Distant and regional adenocarcinoma, large cell carcinoma, and squamous cell carcinoma. Rates are multiplied with ife expectancy by race and ethnicity (NH-White 78.8; NH-Black 74.8; Asian 85.6; Hispanic 80.2) to obtain lifetime risk of aNSCLC estimates^33^
Proportion aNSCLC patients with insufficient tissue for molecular testing		30%	30%	30%	30%		Fixed	Hagemann et al (2015)^10^	This estimate is multiplied with the lifetime risk of aNSCLC estimates
Prevalence of oncogenic driver mutations among NSCLC patients	N* EGFR ALK KRAS ROS1 BRAF NTRK MET RET no driver mutation	2778 12.9% 2.2% 13.8% 1.0% 1.8% 0.1% 2.9% 1.0% 64.3%	93 20.4% 3.2% 9.7% 2.2% 3.2% 1.1% 0.0% 2.2% 58.0%	124 52.4% 4.8% 3.2% 1.6% 0.8% 0.0% 1.6% 1.6% 33.9%	55 14.5% 1.8% 5.5% 0.0% 3.6% 0.0% 3.6% 5.5% 65.4%		Dirichlet by race and ethnicity	Adib et al (2022)^12^	Driver mutations are mutually exclusive.
Prevalence of PD-L1 expression	N* PD-L1 ≥50 PD-L1 1–49 PD-L1 <1	305 33.8% 33.8% 32.5%	59 25.4% 15.3% 59.3%	128 33.6% 32.8% 33.5%	120 70.8% 20.8% 8.3%		Dirichlet by race and ethnicity	Planchard et al^36^; Choudhury et al^37^; Saravia et al (2019)^38^	Distribution assumed applicable in the absence of driver mutations
		N*	TP, FP, TN, FN		Sensitivity	Specificity			
Liquid biopsy - NGS test performance	EGFR	168	0.083; 0.006; 0.875; 0.036		0.700	0.993	Dirichlet for TP, FP, TN, FN by driver mutation. Sampled values used to calculate sensitivity and speciff city as model input.	Pritchett et al (2019)^14^	Test performance is independent of race and ethnicity.
	ALK	301	0.01; 0.003; 0.973; 0.013		0.429	0.997			
	KRAS	147	0.327; 0.007; 0.585; 0.082		0.800	0.989			
	ROS1	301	0.01; 0.003; 0.973; 0.013		0.429	0.997			
	BRAF	151	0.04; 0.007; 0.934; 0.02		0.667	0.993			
	NTRK	301	0.01; 0.003; 0.973; 0.013		0.429	0.997			
	MET	143	0.028; 0.007; 0.937; 0.028		0.500	0.993			
	RET	301	0.01; 0.003; 0.973; 0.013		0.429	0.997			
Tissue biopsy - NGS test performance^†^	EGFR, ALK, KRAS, ROS1, BRAF, NTRK, MET, RET, PD-L1	200	0.495; 0.005; 0.495; 0.005		0.990	0.990	Dirichlet by driver mutation and PD-L1 expr.	Pritchett et al^14^; Torlakovic et al (2020)^39^	Test performance is independent of race and ethnicity.
		Log-scale estimate	SE	Shape estimate	SE	Correlation			
Scale and shape parameter of Weibull for PFS	EGFR	−0.5511	0.0514	0.1378	0.0313	−0.5863	Bivariate normal distribution by driver mutation and PD-L1 expression	Li et al (2019)^40^	See drug costs for corresponding treatments; Scale and shape parameters the same for all subgroups with the exception of EGFR. (See next parameter).
	ALK	−0.0388	0.0480	−0.0542	0.0377	−0.1127		Jahanzeb et al (2020)^41^	
	KRAS^‡^	−0.1532	0.0478	−0.3170	0.0955	0.0000		Sun et al (2021)^42^	
	ROS1	−0.6338	0.1415	−0.1189	0.1177	−0.2741		Doebele et al (2021)^43^	
	BRAF	−0.1386	0.1859	0.0855	0.1387	−0.3093		Johnson et al (2022)^44^	
	NTRK	−1.1810	0.4178	−0.1083	0.3434	−0.2122		Drilon et al (2022)^45^	
	MET	−0.7555	0.1891	−0.0202	0.1514	0.0313		Paik et al (2022)^46^	
	RET	−0.3707	0.1547	0.2126	0.1247	0.1699		Popat et al (2022)^47^	
	PD-L1 ≥50	−0.5142	0.0954	0.1492	0.0628	−0.4095		Velcheti et al (2022)^48^	
	PD-L1 1–49	0.2313	0.1264	−0.0046	0.1027	0.0789		Velcheti et al (2021)^49^	
	Wild type	0.2879	0.1254	0.1068	0.0968	0.0752		Velcheti et al (2021)^49^	
Scale and shape parameter of Weibull for OS	EGFR	−1.1604	0.0659	0.1046	0.0364	−0.7443	Bivariate normal distribution by driver mutation and PD-L1 expression	Li et al (2019)^40^	
	ALK	−1.0618	0.0675	−0.1311	0.0507	−0.4514		Jahanzeb et al (2020)^41^	
	KRAS	−0.7059	0.1091	−0.3170	0.0955	−0.0138		Sun et al (2021)^42^	
	ROS1	−1.6419	0.2177	−0.1847	0.1820	−0.3954		Doebele et al (2021)^43^	
	BRAF	−1.2094	0.2811	0.1381	0.1794	−0.6516		Johnson et al (2022)^44^	
	NTRK	−2.2890	0.6915	0.4589	0.3686	−0.6907		Drilon et al (2022)^45^	
	MET	−1.2892	0.2050	0.2974	0.1469	−0.3873		Paik et al (2022)^46^	
	RET	−1.7389	0.2681	−0.1642	0.2318	0.0811		Popat et al (2022)^47^	
	PD-L1 ≥50	−1.1603	0.1114	0.0659	0.0761	−0.5963		Velcheti et al (2022)^48^	
	PD-L1 1–49	−0.6783	0.1573	0.0910	0.1312	−0.3198		Velcheti et al (2021)^49^	
	wild type	−0.6930	0.1594	0.1994	0.1290	−0.2920		Velcheti et al (2021)^49^	
Adjustment of PFS and OS with EGFR (HR)^§^		NH-White	NH-Black	Asian	Hispanic		Fixed	Ramalingam et al^50^;	
		0.675	0.675	0.75	0.675			Gibson et al (2019)^51^	
Effect of mismatched treatment on PFS and OS due to false test results		HR	95% CI low	95% CI high			Normal distribution for log-transformed HRs	Liu et al (2022)^52^	
	FP EGFR	1.85	1.26	2.72					
	FP ALK	3.45	2.13	5.57					
	FP KRAS	1.03	0.93	1.14					
	FP ROS1	1.06	0.93	1.22					
	FP BRAF	1.16	1.03	1.31					
	FP NTRK	1.30	1.10	1.54					
	FP MET	1.18	0.99	1.39					
	FP RET	1.59	1.10	2.28					
	TP PD-L1 + FN mut.	1.34	0.89	2.01					
	FP PD-L1 + FN mut.	1.58	1.05	2.37					
	FN PD-L1 + FN mut.	1.14	0.76	1.70					
	TN PD-L1 + FN mut.	1.34	0.89	2.01					
	FP PD-L1 + TN mut.	1.18	0.97	1.44					
	FN PD-L1 + TN mut.	0.85	0.70	1.03					
Calibration of PFS and OS (HR)		NH-White	NH-Black	Asian	Hispanic		Fixed	SEER^53^	
		1.345	1.461	1.419	1.432				
Impact of faster TAT and treatment initiation with LB than TB on PFS and OS		HR	95% CI low	95% CI high			Normal distribution for log-transformed HRs	Shokoohi et al (2021)^54^;	Applicable to PFS and OS for all treatments and test results; See online supplement for details
	3 weeks (base-case)	0.72	0.56	0.93				Raez et al (2022)^55^	
	1 week	0.90	0.82	0.98					
Utility		Estimate	95% CI low	95% CI high			Beta	Chouaid et al (2013)^56^	Assumed the same for all subgroups
	Pre-progression	0.71	0.67	0.76					
	Post-progression	0.67	0.59	0.75					
Probability complication with rebiopsy		Estimate	SE				Beta	Vanderpoel et al (2022)^57^	Assumed the same for all subgroups; se assumed at 5% of estimate
		0.073	0.00365						
		Medicare estimate	SE	Commercial estimate	SE				
Type of insurance		67.4%		32.6%			Fixed	Ganti et al (2021)^59^	Assumed the same for all race and ethnicity subgroups.
Cost LB NGS (US$)		3,425		6,722			Fixed	Vanderpoel et al (2022)^57^	Inflated to 2022
Cost TB NGS (US$)	Rebiopsy	324		1,628					
	rebiopsy w/compl.	4,020		18,290					
	NGS tissue	1,773		4,758					
Post-diagnosis disease management cost (annualized; US$)	Pre-progression	12,254	613	42,875	2,144		Gamma	Stargardter et al (2021)^58^	See online supplement for details; Inflated to 2022; Same for all subgroups; se assumed at 5% of estimate.
	Post-progression	85,250	4,263	153,680	7,684				
Annualized drug costs (US$)^ǁ^			Pre-progression			Post-progression	Fixed	NCCN guidelines^6^; FSS; Bains et al (2022)^60^	
			Year 1	Year 2	Year 2+				
EGFR		1L:osimertinib; 2L:afatinib+cetuximab	178 132	178 132	178 132	83 423			
ALK		1L: alectinib, brigatinib, or lorlatinib; 2L: lorlatinib or pembrolizumab + carboplatin + pemetrexed	205 476	205 476	205 476	54 132			
KRAS		1L: pembrolizumab + carboplatin + pemetrexed (pembrocarb-pem); 2L: sotorasib	185 608	185 465	7803	58 484			
ROS1		1L: entrectinib; 2L: lorlatinib	221 185	221 185	221 185	56 714			
BRAF		1L: dabrafenib + trametinib; 2L: pembrocarb-pem	313 415	313 415	313 415	57 477			
NTRK		1L: arotrectinib; 2L: pembro-carb-pem	406 928	406 928	406 928	57 477			
MET		1L: tepotinib 2L: pembro-carb-pem	185 711	185 711	185 711	57 477			
RET		1L: pralsetinib; 2L: pembro-carb-pem	237 320	237 320	237 320	57 477			
PD-L1 >.=50		1L: pembro; 2L: docetaxel	180 187	180 187	-	1400			
PD-L1 1–49		1L: pembro-carb-pem (nonsquamous)/pembro-carb-cisplatin (squamous); 2L: docetaxel	197 393	183 115	5453	738			
PD-L1 <1		1L: pembro-carb-pem (nonsquamous)/pembro-carb-cisplatin (squamous); 2L: docetaxel	195,869	183 383	5721	738			

a NSCLC indicates advanced non–small cell lung cancer; BSC, best supportive care; Chemo, chemotherapy; Expr., expression; FN, false negative; FP, false positive; FSS, Federal supply schedule; HR, hazard ratio; IO, immunotherapy; KFF, Kaiser Family Foundation; LB, liquid biopsy; Mut., mutant; NCCN, National Comprehensive Cancer Network; NGS, next-generation sequencing; NH, non-Hispanic; OS, overall survival; PFS, progression-free survival; RCT, randomized controlled trial; SEER, Surveillance, Epidemiology, and End Results; TKI, tyrosine kinase inhibitor; TN, true negative; TP, true positive; US, United States.

* Ns are presented to help interpret the “degree of uncertainty” in the percentages as captured with the Dirichlet distribution.

† TB considered gold standard. For each mutation and PD-L1 expression we assumed 99% sensitivity and specificity with uncertainty based on n = 200 and 50% cases.

‡ PFS scale and shape imputed based on KRAS OS scale and shape in combination with the ratio between PFS and OS for scale and shape for other mutations. Correlation assumed 0 given negligible correlation between scale and shape for KRAS OS.

§ Used real-world evidence (RWE) for EGFR is non-osimertinib TKI treatment. HR for osimertinib versus other TKI from RCT applied to the estimated RWE PFS and OS scale parameters to obtain RWE PFS and OS scale parameters with osimertinib; Efficacy of osimertinib vs other TKIs in Asian patients is different from non-Asian patients.

ǁ Assumption: Second-line (2L) cost estimate includes the assumption that 50% of patients get BSC without active drug therapy after first-line (1L); adjustment of post-progression drug cost based on duration of 2L, informed by ratio of 2L median PFS and OS as obtained from literature: after PD-L1 mono, 2L single agent chemo PFS/OS = 4.56 / 6.59 (months) = 0.692; after PD-L1+chemo, 2L single agent chemo PFS/OS = 2.56 / 7.02 = 0.365^60^; after any IO, 2L IO PFS/OS = 5.5 / 10.7 = 0.514; after any IO, 2L ChemoIO PFS/OS = 4.9 / 7.9 = 0.620; after any IO, 2L Chemo PFS/OS: 4.9 / 8.4 = 0.583.^61^

**Table 2.** Expected discounted QALYs per patient, discounted costs related to diagnostic workup per patient, iNHB per 100 000 individuals of the general population factoring in equally distributed opportunity costs related to diagnostic workup at a threshold of $150 000, and QALE per member of the general population by race and ethnicity without and with LB-first.

Outcome	Group (proportion)	Tissue biopsy only		Liquid biopsy first		Difference	
		mean	95% uncertainty interval	mean	95% uncertainty interval	mean	95% uncertainty interval
QALYs (per patient)	NH-White (66.77%)	1.38	(1.11–1.74)	1.58	(1.23–2.06)	0.20	(0.07–0.38)
	NH-Black (16.24%)	1.38	(1.09–1.75)	1.59	(1.22–2.09)	0.21	(0.07–0.41)
	Asian (5.83%)	1.67	(1.37–2.06)	1.98	(1.56–2.50)	0.31	(0.09–0.61)
	Hispanic (11.15%)	1.46	(1.18–1.84)	1.63	(1.28–2.07)	0.17	(0.05–0.35)
	All (100%)	1.41	(1.13–1.77)	1.61	(1.26–2.09)	0.21	(0.07–0.39)
Costs (US$, diagnostic workup, per patient)	NH-White (66.77%)	4072	(4016–4127)	7398	(7064–7731)		
	NH-Black (16.24%)	4072	(4016–4127)	7267	(6847–7698)		
	Asian (5.83%)	4072	(4016–4127)	6608	(6099–7125)		
	Hispanic (11.15%)	4072	(4016–4127)	7503	(7041–7963)		
	All (100%)	4072	(4016–4127)	7342	(7011–7699)	3270	(2934–3633)
Incremental NHB* (in QALYs per 100 000 individuals of the general population)	NH-White (60.57%)					99	(28–195)
	NH-Black (13.89%)					112	(32–224)
	Asian (6.23%)					131	(32–273)
	Hispanic (19.31%)					37	(4–89)
	All (100%)					91	(25–182)

		Pre-LB-first distribution	Post LB-first distribution	
QALE (per individual general population)	NH-White (60.57%)	68.798	68.799	(68.798–68.800)
	NH-Black (13.89%)	65.446	65.448	(65.447–65.449)
	Asian (6.23%)	74.878	74.880	(74.879–74.881)
	Hispanic (19.31%)	71.762	71.762	(71.762–71.763)
	All (100%)	69.283	69.284	(69.284–69.285)

a NSCLC indicates advanced non–small cell lung cancer; iNHB, incremental net health benefit; LB, liquid biopsy; NH, non-Hispanic; NHB, net health benefit; QALE, quality-adjusted life expectancy at birth; QALY, quality-adjusted life-year; TB, tissue biopsy.

* Presented estimates by race and ethnicity are incremental QALYs per 100 000 individuals of each race and ethnicity subgroup of the general population. The incremental NHB by race and ethnicity reflects the health gains by race and ethnicity in the general population as a result of using LB-first instead of TB-only to inform first-line therapy for aNSCLC minus the health opportunity costs (ie, health losses) by race and ethnicity in the general population because of the greater costs of using LB-first ($3270 per patient), which are evenly divided over all members of the general population.

**Table 3.** Inequality metrics for expected QALYs per patient and expected QALE per individual of the general population factoring in equally distributed opportunity costs related to diagnostic workup at a threshold of $150 000 and different degrees of inequality aversion.

Outcome	Metric	Inequality aversion	Tissue biopsy only		Liquid biopsy first		Difference*	
			mean	95% uncertainty interval	mean	95% uncertainty interval	mean	95% uncertainty interval
QALYs (per patient)	Atkinson	0.9	0.00134	(0.00048–0.00256)	0.00170	(0.00063–0.00328)	0.00036	(−0.00014 to 0.00118)
		5	0.00627	(0.00234–0.01184)	0.00760	(0.00303–0.0143)	0.00134	(−0.00077 to 0.00487)
		11 (base-case)	0.01109	(0.00439–0.02149)	0.01291	(0.00542–0.02404)	0.00182	(−0.00145 to 0.00803)
		15	0.01337	(0.00535–0.02654)	0.01530	(0.00657–0.02966)	0.00193	(−0.00201 to 0.01026)
	Kolm	0.025	0.00008	(0.00003–0.00015)	0.00013	(0.00005–0.00028)	0.00006	(0.00000–0.00017)
		0.1	0.00031	(0.00013–0.00061)	0.00053	(0.0002–0.00111)	0.00022	(0.00001–0.00065)
		0.15 (base-case)	0.00046	(0.0002–0.00091)	0.0008	(0.00029–0.00166)	0.00033	(0.00002–0.00097)
		0.3	0.00092	(0.00039–0.0018)	0.00157	(0.00058–0.00327)	0.00065	(0.00003–0.0019)
QALE (per individual general population)	Atkinson	0.9	0.0004948	(0.0004948–0.0004948)	0.0004947	(0.0004947–0.0004948)	−0.0000001	(−0.0000001 to 0.0000000)
		5	0.0027011	(0.0027011–0.0027011)	0.0027008	(0.0027004–0.002701)	−0.0000004	(−0.0000008 to −0.0000001)
		11 (base-case)	0.0058032	(0.0058032–0.0058032)	0.0058024	(0.0058015–0.005803)	−0.0000008	(−0.0000018 to −0.0000002)
		15	0.0077995	(0.0077995–0.0077995)	0.0077984	(0.0077971–0.0077992)	−0.0000011	(−0.0000024 to −0.0000003)
	Kolm	0.025	0.065946	(0.065946–0.065946)	0.0659395	(0.0659311–0.0659447)	−0.0000065	(−0.0000149 to −0.0000013)
		0.1	0.2569327	(0.2569327–0.2569327)	0.2569052	(0.2568691–0.2569265)	−0.0000275	(−0.0000635 to −0.0000061)
		0.15 (base-case)	0.3791431	(0.3791431–0.3791431)	0.3791010	(0.3790459–0.3791334)	−0.0000421	(−0.0000972 to −0.0000097)
		0.3	0.7259447	(0.7259447–0.7259447)	0.7258599	(0.7257466–0.7259244)	−0.0000848	(−0.0001981 to −0.0000203)

QALE indicates quality-adjusted life expectancy at birth; QALY, quality-adjusted life-year.

* Difference: Atkinson_LB-first_ – Atkinson_TB-only_ or Kolm_LB-first_ – Kolm_TB-only_. A positive number implies an increase in inequality.
