# Supplementary material for: The Health Inequality Impact of Liquid Biopsy to Inform First-Line Treatment of Advanced Non–Small Cell Lung Cancer: A Distributional Cost-Effectiveness Analysis
Source: Value Health. Author manuscript; Available in PMC 2024 Feb 12. (PMC10859998; doi:10.1016/j.jval.2023.08.010)
Supplement: Supplement [file NIHMS1965263-supplement-Supplement.pdf]

ONLINE SUPPLEMENT

Process of estimating health inequality impact in target patient population and general population **3**

Model structure **4**

Quantifying the impact of faster treatment with LB than with TB **6**

PFS and OS curves by test result with LB-first and TB-only strategies **7**

Model input: Resource use and unit costs associated with treatment and disease management **34**

- Table S1: Model input - Treatment costs with 1st line therapy according to driver mutation 35
- Table S2: Model input - Treatment costs with 2nd line therapy according to initial driver mutation 40
- Table S3: Model input – Unit costs associated with disease management 42
- Table S4: Model input – Resource use and costs associated with disease management 43

Calculating net health benefit **44**

Atkinson and Kolm inequality equations **45**

Additional results **45**

- Table S5: Expected discounted QALYs per patient, discounted costs related to diagnostic workup, treatment, and disease management per patient, incremental net health benefit (iNHB) per 100,000 individuals of the general population factoring in equally distributed opportunity costs at a threshold of $150k, and QALE per member of the general population by race and ethnicity without and with LB-first. 46

- Table S6: Inequality metrics for expected QALYs per patient and expected QALE per individual of the general population factoring in equally distributed

opportunity costs related to diagnostic workup, treatment, and disease management at a threshold of $150k and different degrees of inequality aversion. 47

- Table S7: Estimated iNHB with LB-first per 100,000 individuals of the general population factoring in equally distributed opportunity costs at a threshold of $50k, $100k, $150k, $200k by race and ethnicity. 49

- Table S8: Inequality metrics for QALE per individual of the general population factoring in equally distributed opportunity costs related to diagnostic workup at threshold of $50k, $100k, $150k, $200k for different degrees of inequality aversion. 50

- Table S9: Inequality metrics for QALE per individual of the general population factoring in equally distributed opportunity costs related to diagnostic workup, treatment, and disease management at threshold of $50k, $100k, $150k, $200k for different degrees of inequality aversion. 53

- Table S10: Expected discounted QALYs per patient, discounted costs related to diagnostic workup per patient, iNHB per 100,000 individuals of the general population factoring in equally distributed opportunity costs at a threshold of $150k, and QALE per member of the general population by race and ethnicity without and with LB-first under the assumption of a 1-week shorter TAT with LB-first. 56

- Table S11: Inequality metrics for expected QALYs per patient and expected QALE per individual of the general population factoring in equally distributed opportunity costs related to diagnostic workup at a threshold of $150k and different degrees of inequality aversion under the assumption of a 1-week shorter TAT with LB-first. 57

Process of estimating health inequality impact in target patient population and general population

This overview shows the process of estimating the health inequality impact in the target patient population and general population along with information where the (intermediate) results are presented in the manuscript with tables and figures.

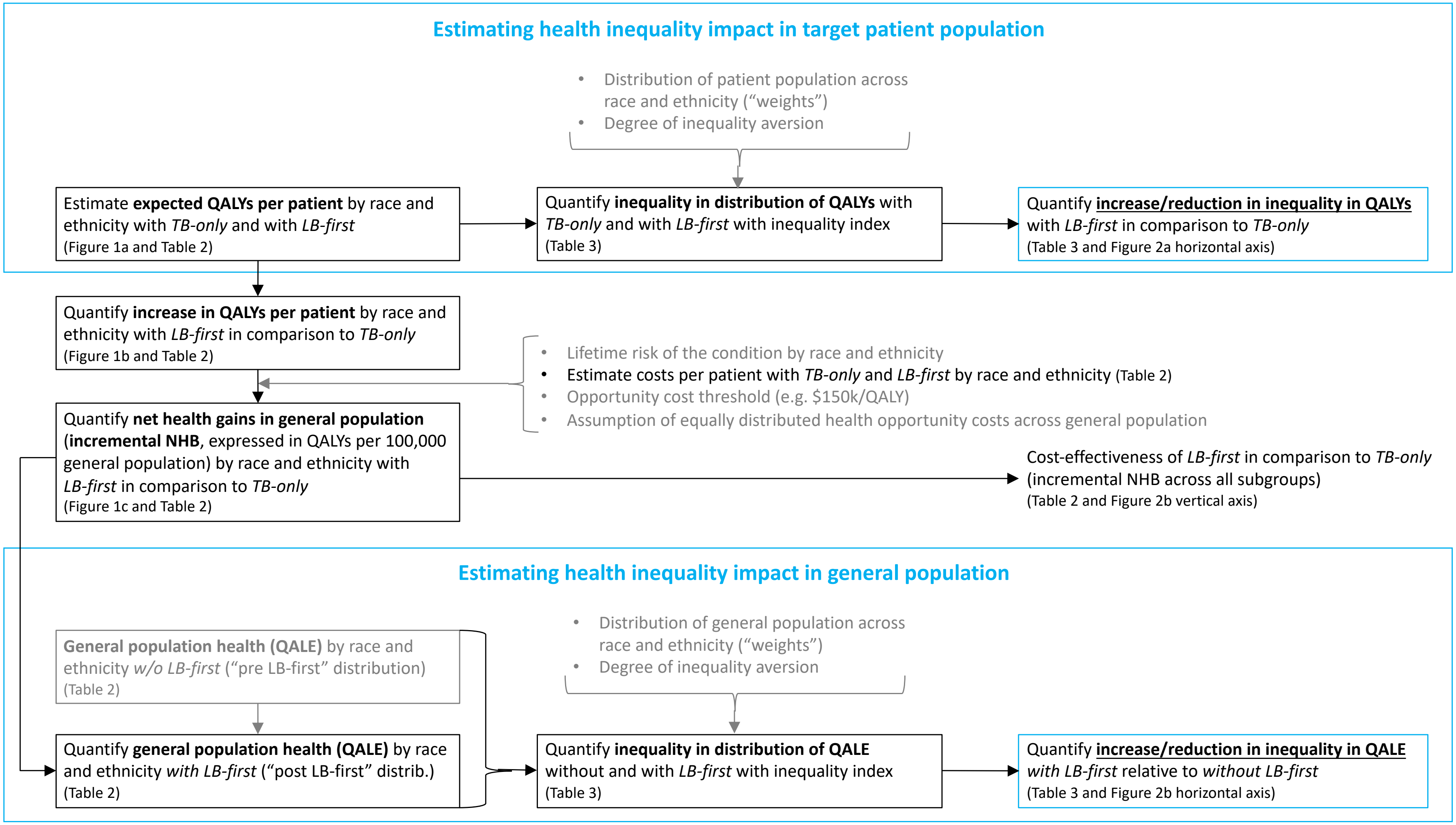

Note: Text in black reflects information that is obtained with analyses performed Text in grey reflects external information (not obtained with analyses performed)

Model structure

The model consisted of a tree structure reflecting the possible test outcomes with the liquid biopsy (LB)-first and tissue biopsy (TB)-only strategies (see Figure S1). Expected progression-free survival (PFS) and overall survival (OS) for each test-informed first-line (1L) treatment were obtained with a partitioned survival modeling (PSM) approach. With 3-state PSMs, the probability that a patient is in each of three distinct health states, pre-progression, post-progression, and death, at a given point of time when treated with a particular therapy informed by a test result was simulated (Figures S3-S28). State membership was estimated from PFS and OS survival curves using an "area under the curve" approach. Lifetime quality adjusted life years (QALYs) and costs associated with treatment for each final branch were computed using the time present value given a flow of state values (i.e., utility and annualized treatment costs) that change as patients transition between health states or as costs vary as a function of time since treatment initiation. Expected QALYs and costs were estimated for each subgroup by "folding back the tree" given its conditional probabilities, costs associated with each test performed as defined by the decision tree, and QALYs and costs associated with treatment from the PSMs for each final branch of the tree.

Figure S1: Model structure reflecting LB-first and TB-only strategies

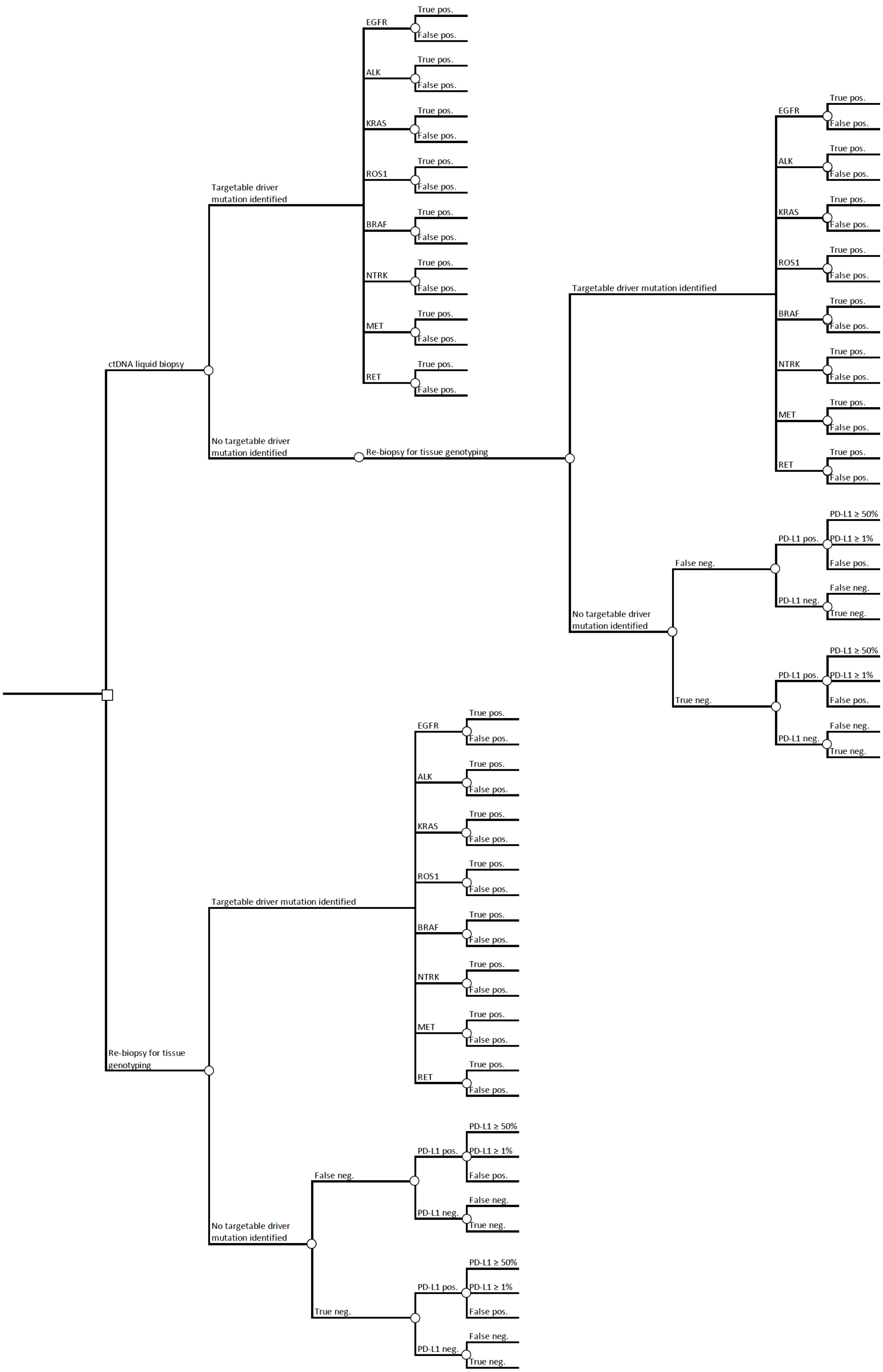

Quantifying the impact of faster treatment with LB than with TB

The impact of a shorter turn-around time (TAT) and therefore faster treatment initiation with LB relative to TB on PFS and OS was based on data presented by Shokoohi et al. (2021). While waiting for test results, 1.5 weeks with LB and 4.5 weeks with TB, patients experience mortality according to best supportive care (BSC) followed by mortality according to immuno-oncology (IO) therapy once treatment starts, as depicted in Figure S2. Based on these created Kaplan Meier curves from the data by Shokoohi et al., a hazard ratio (HR) of 0.72 (95%CI 0.56 - 0.93) was estimated that was subsequently applied to all treatment-specific PFS and OS curves in the model to adjust for faster treatment with LB. This 3-week HR was translated into an HR for a 1-week difference in TAT for sensitivity analyses according to $\log(HR_{1\text{-week}}) = \log(HR_{3\text{-weeks}})/3$ thereby assuming that an HR for 0-week faster treatment initiation equals 1.

Figure S2: 4.5 and 1.5 weeks of waiting for results of molecular workup followed by IO therapy to estimate the hazard ratio (HR) of 3 weeks faster treatment initiation with liquid biopsy.

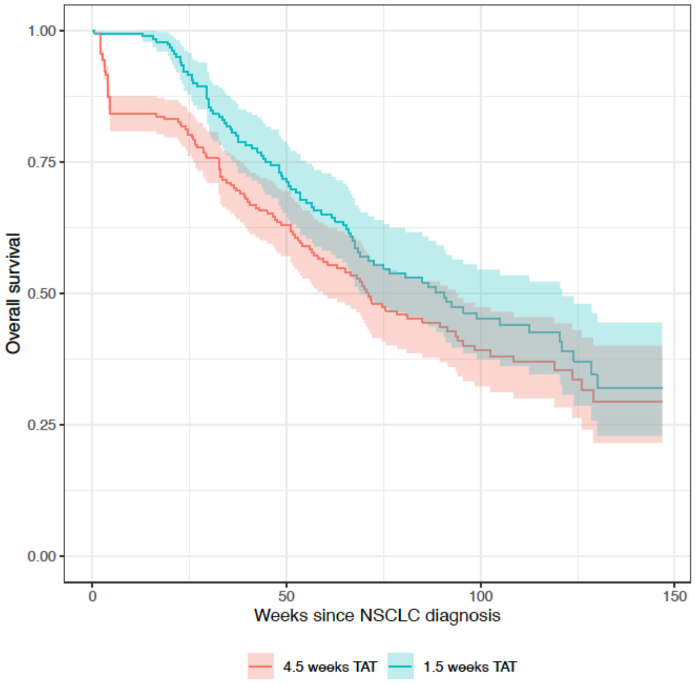

PFS and OS curves by test result with LB-first and TB-only strategies

In Figures S3-S28, the simulated PFS and OS curves are presented corresponding to each final branch of the decision tree stratified by subgroup according to race and ethnicity and LB-first or TB-only strategy. The curves were simulated based on the Weibull scale and shape parameter estimates obtained by analyzing pseudo-individual patient data created from digitized published Kaplan-Meier curves for the relevant treatment studies, and the scale parameter adjusted for the impact of a false test result and shorter time to treatment initiation where applicable, and calibration to SEER 5-year conditional survival data, as outlined in the paper.

Figure S3: PFS and OS curves with treatment for true positive EGFR mutation with LB-first and TB-only strategy (Branch 1)

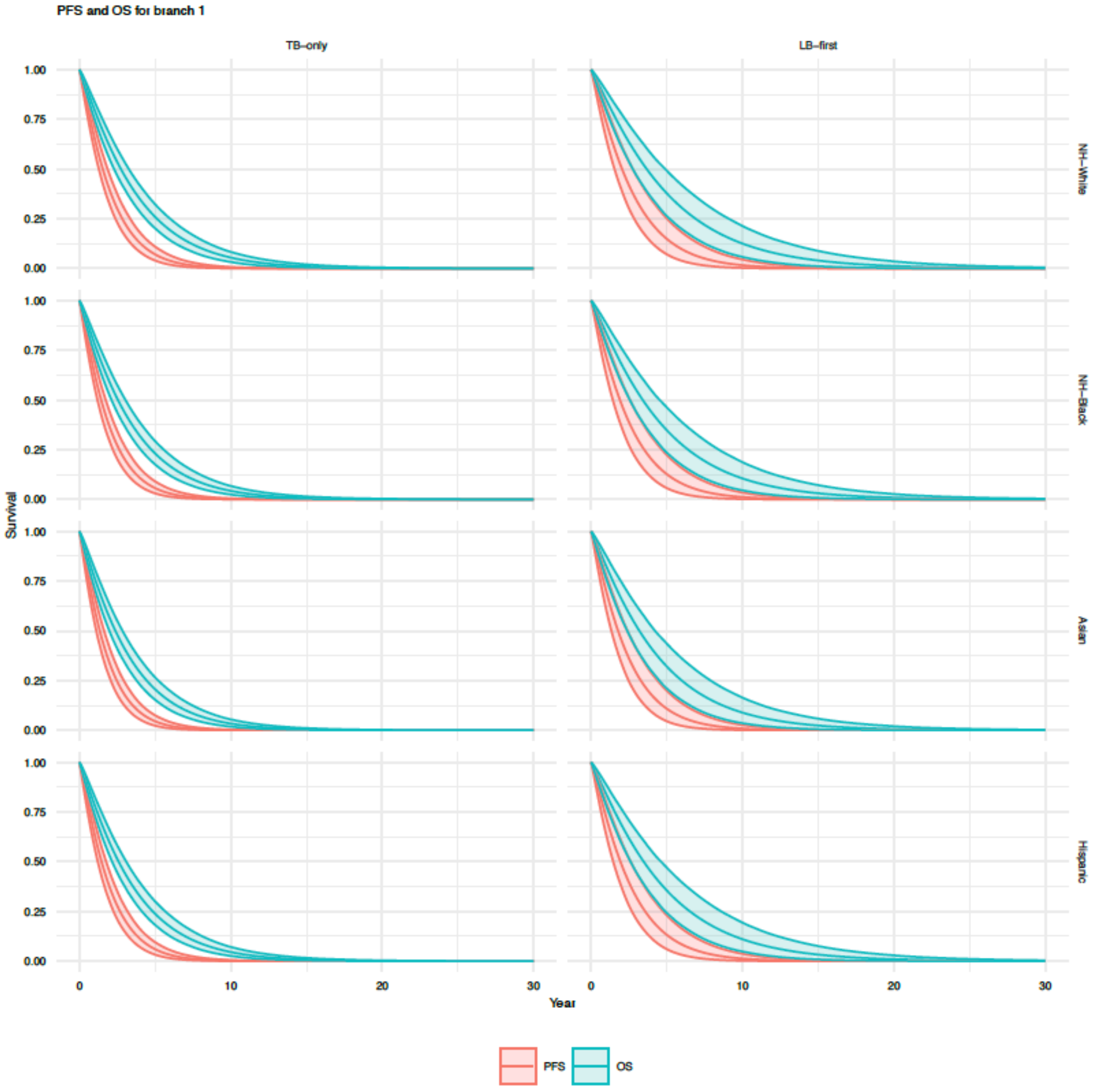

Figure S4: PFS and OS curves with treatment for false positive EGFR mutation with LB-first and TB-only strategy (Branch 2)

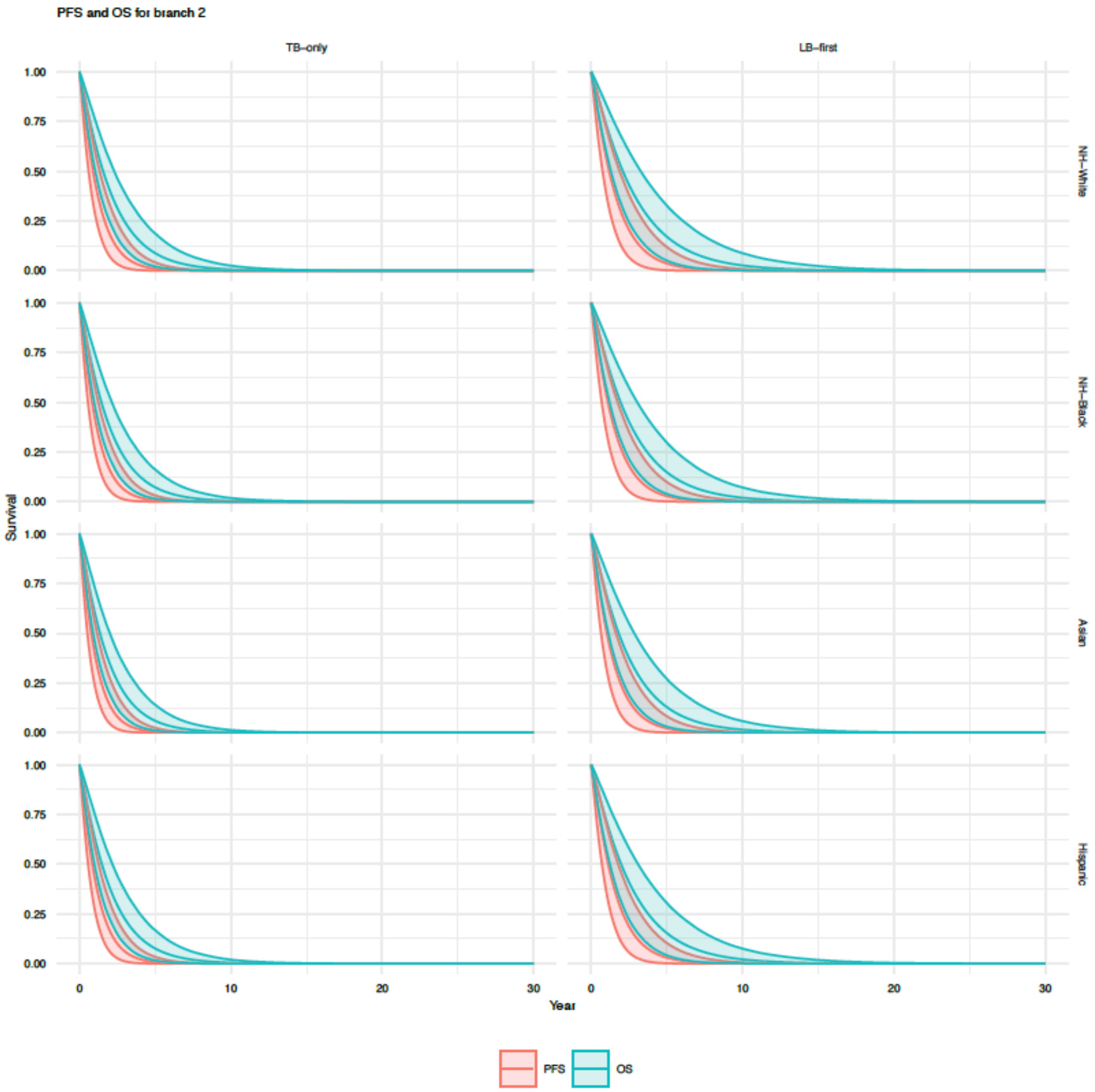

Figure S5: PFS and OS curves with treatment for true positive ALK mutation with LB-first and TB-only strategies (Branch 3)

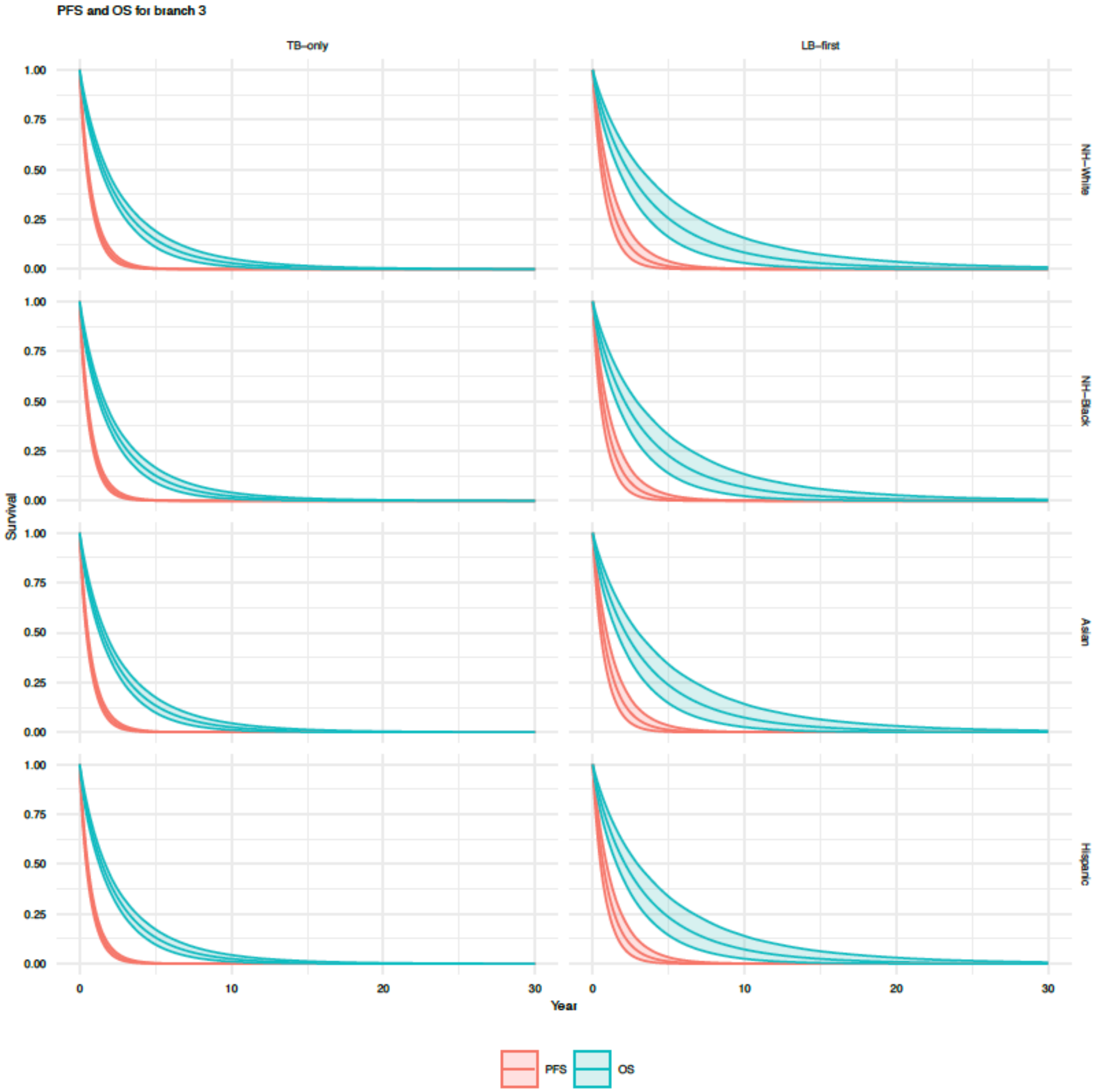

Figure S6: PFS and OS curves with treatment for false positive ALK mutation with LB-first and TB-only strategies (Branch 4)

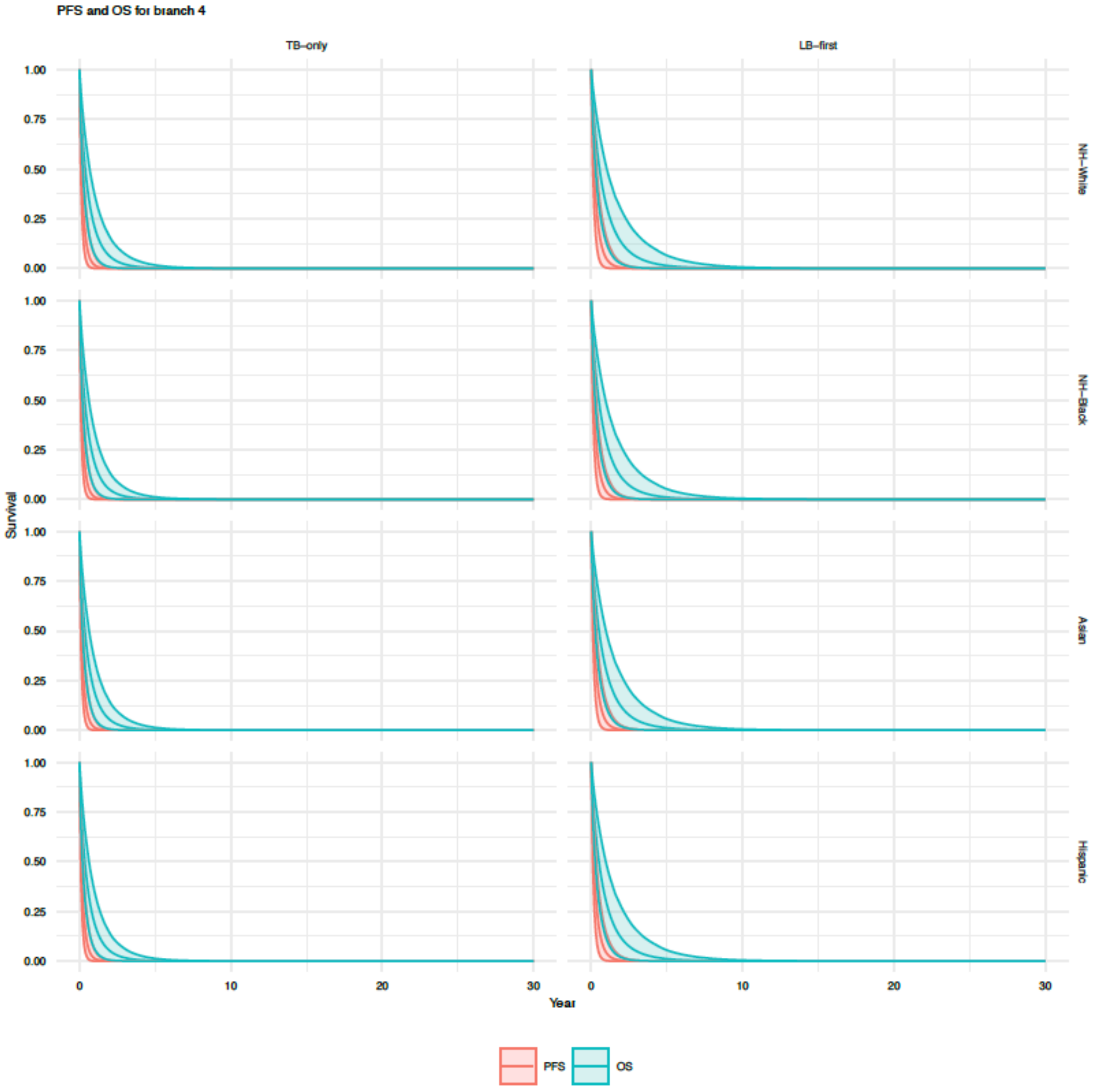

Figure S7: PFS and OS curves with treatment for true positive KRAS mutation with LB-first and TB-only strategies (Branch 5)

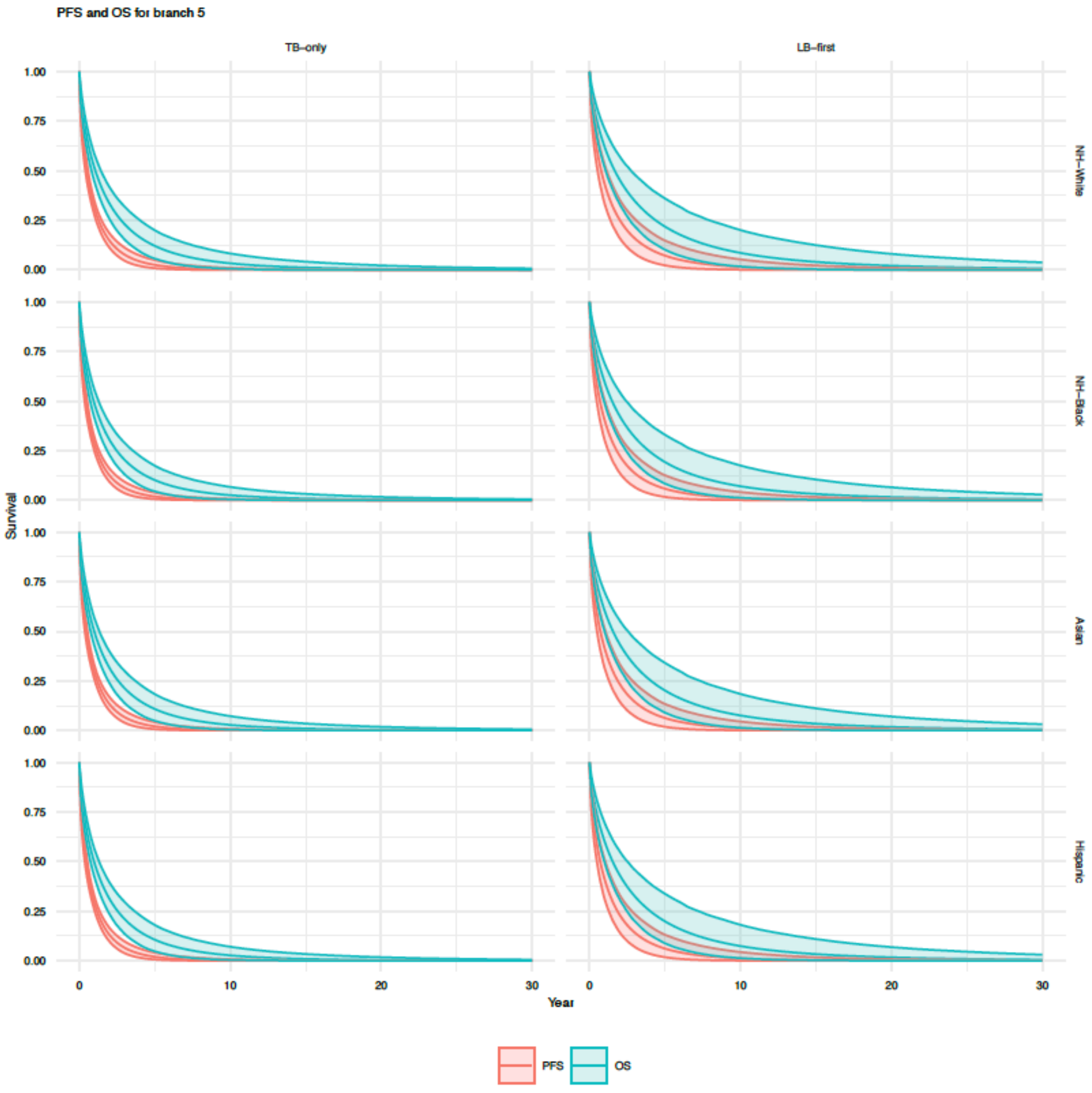

Figure S8: PFS and OS curves with treatment for false positive KRAS mutation with LB-first and TB-only strategies (Branch 6)

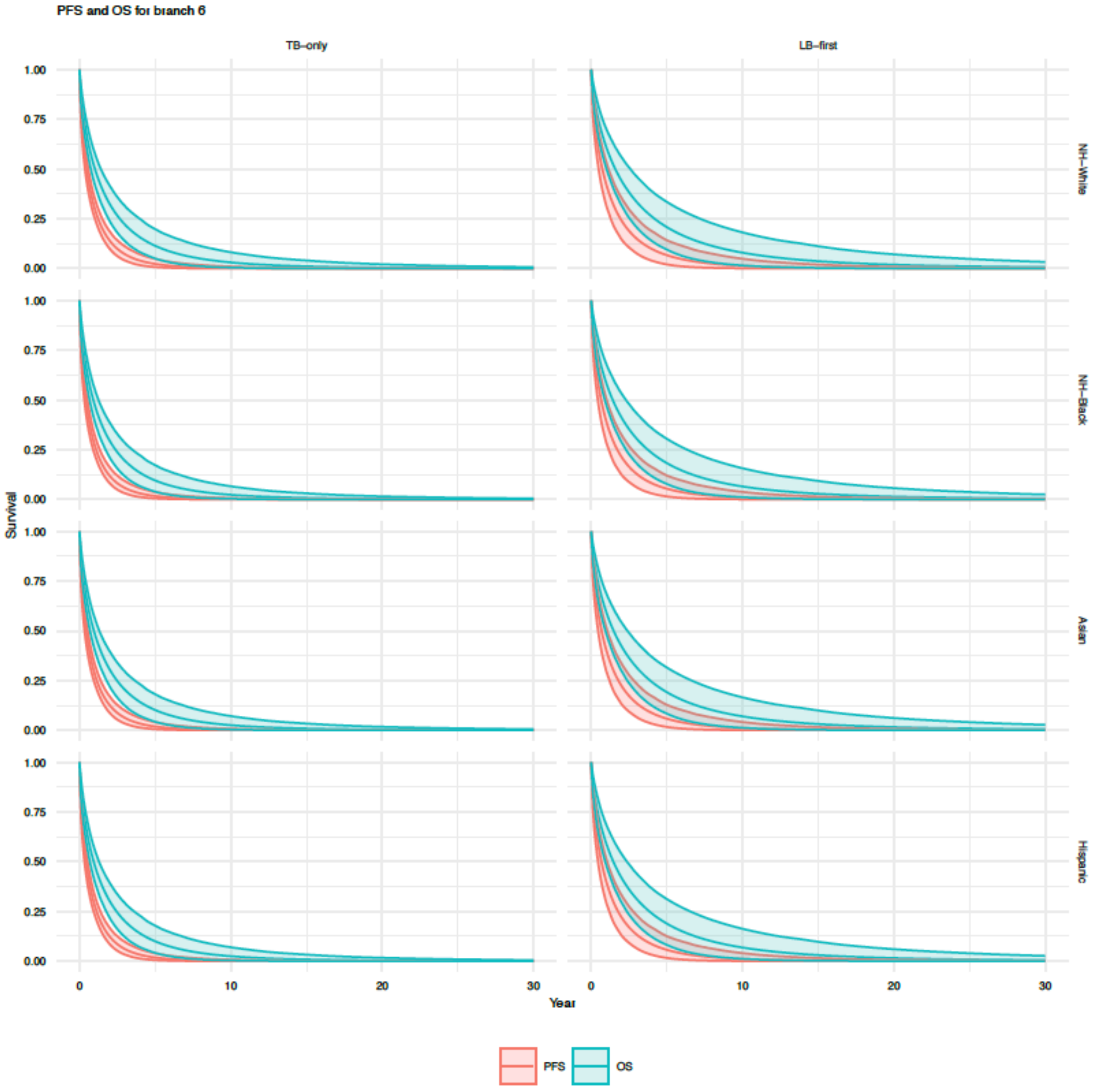

Figure S9: PFS and OS curves with treatment for true positive ROS1 mutation with LB-first and TB-only strategies (Branch 7)

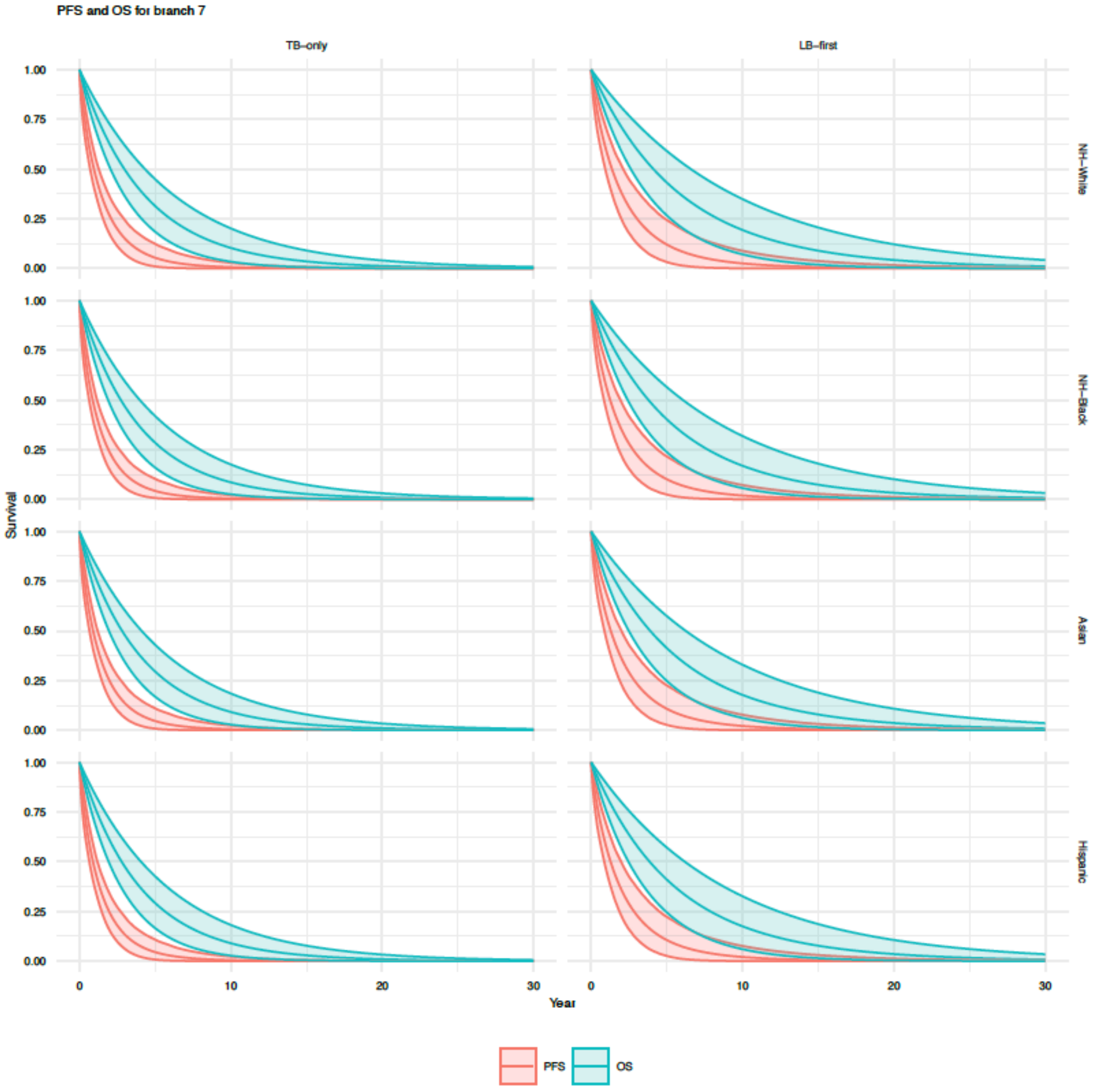

Figure S10: PFS and OS curves with treatment for false positive ROS1 mutation with LB-first and TB-only strategies (Branch 8)

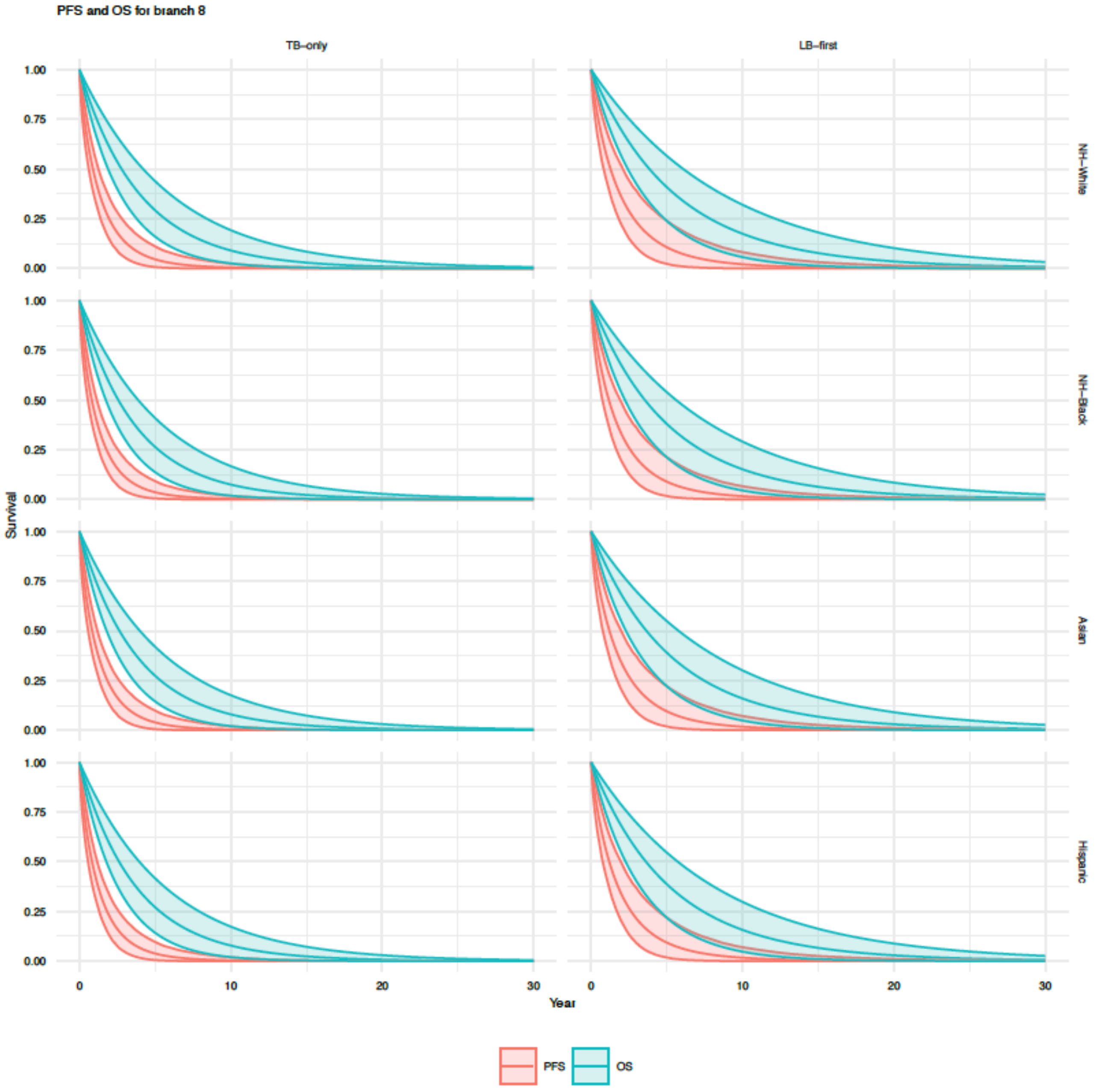

Figure S11: PFS and OS curves with treatment for true positive BRAF mutation with LB-first and TB-only strategies (Branch 9)

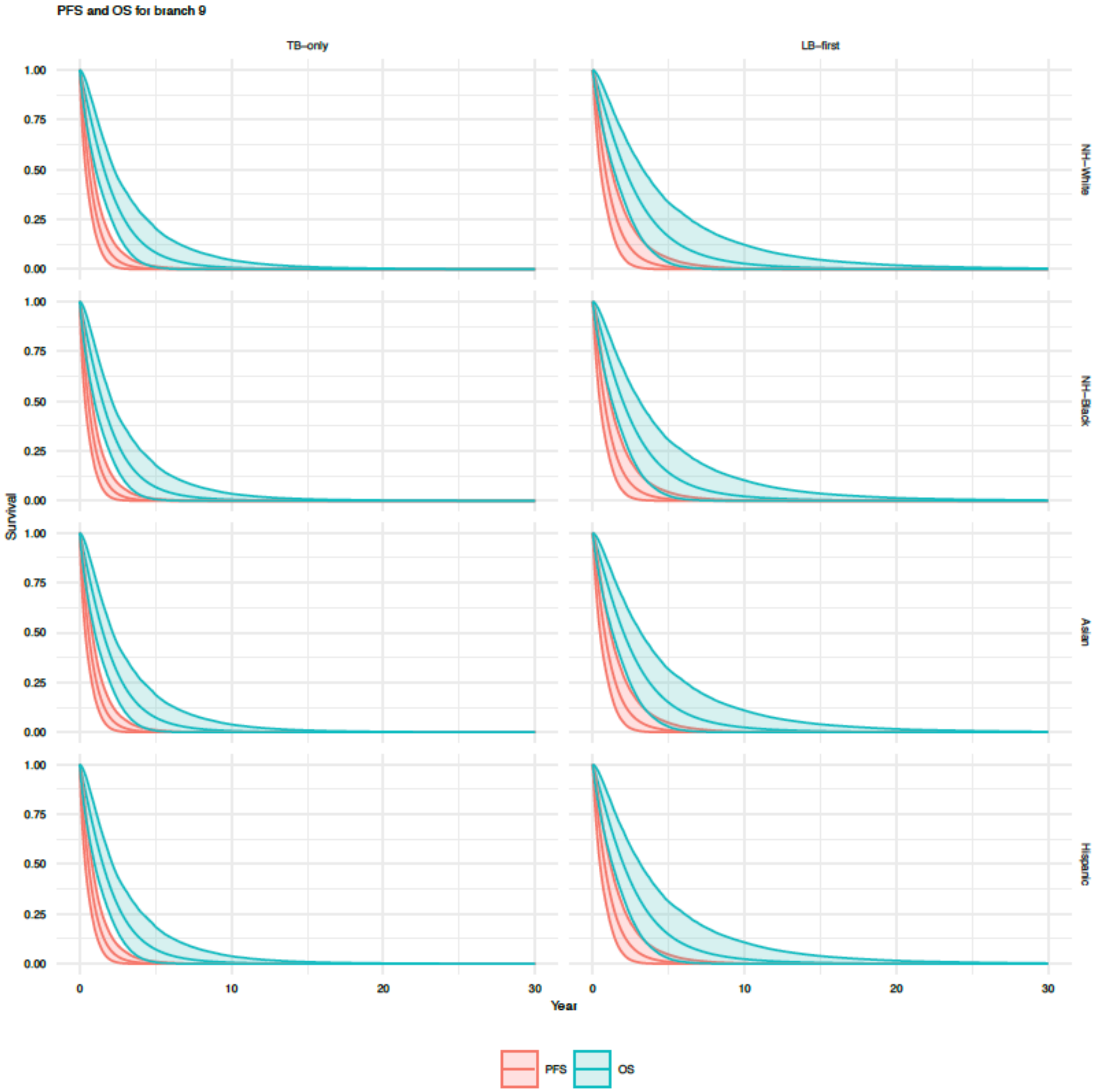

Figure S12: PFS and OS curves with treatment for false positive BRAF mutation with LB-first and TB-only strategies (Branch 10)

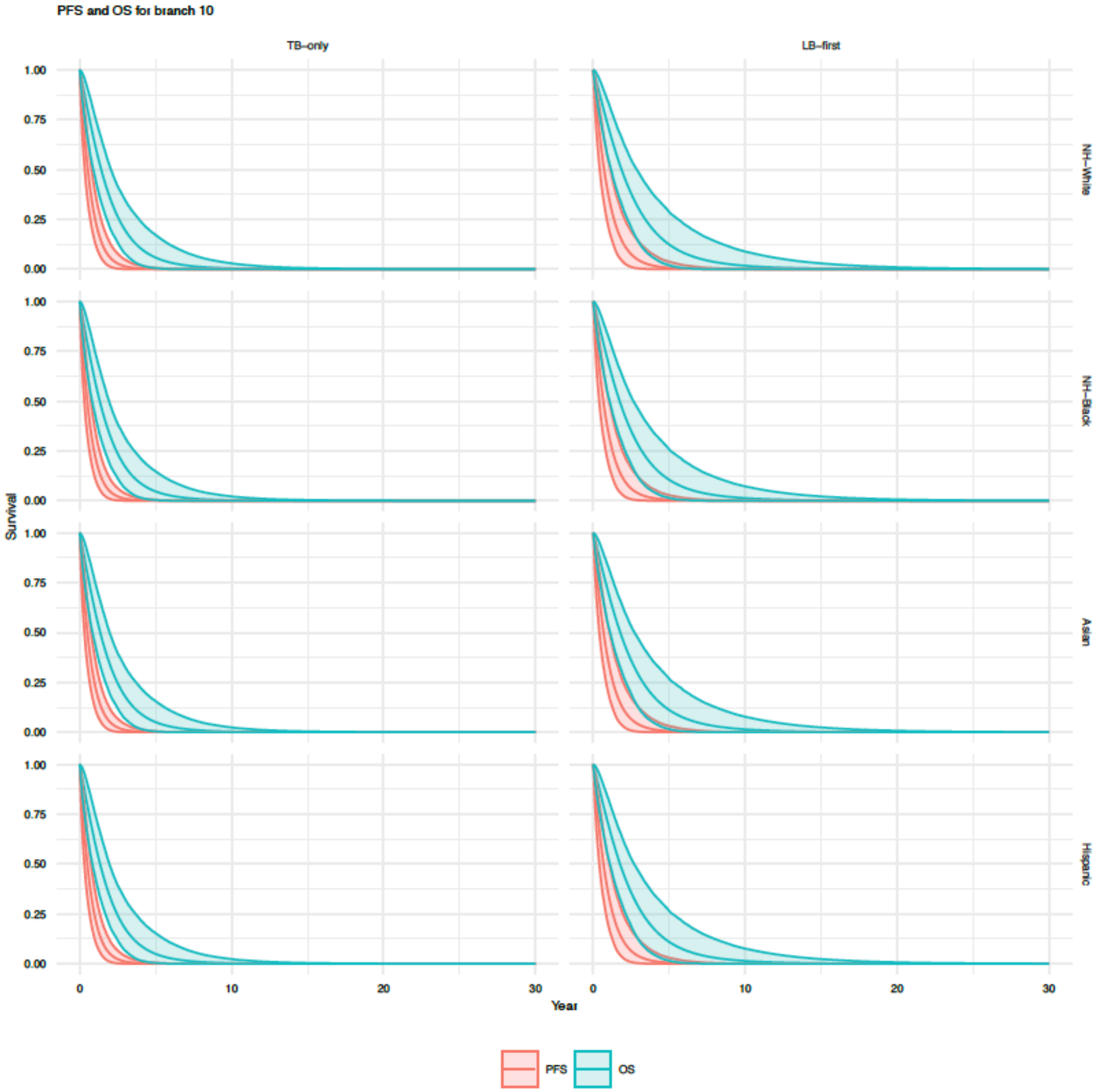

Figure S13: PFS and OS curves with treatment for true positive NTRK mutation with LB-first and TB-only strategies (Branch 11)

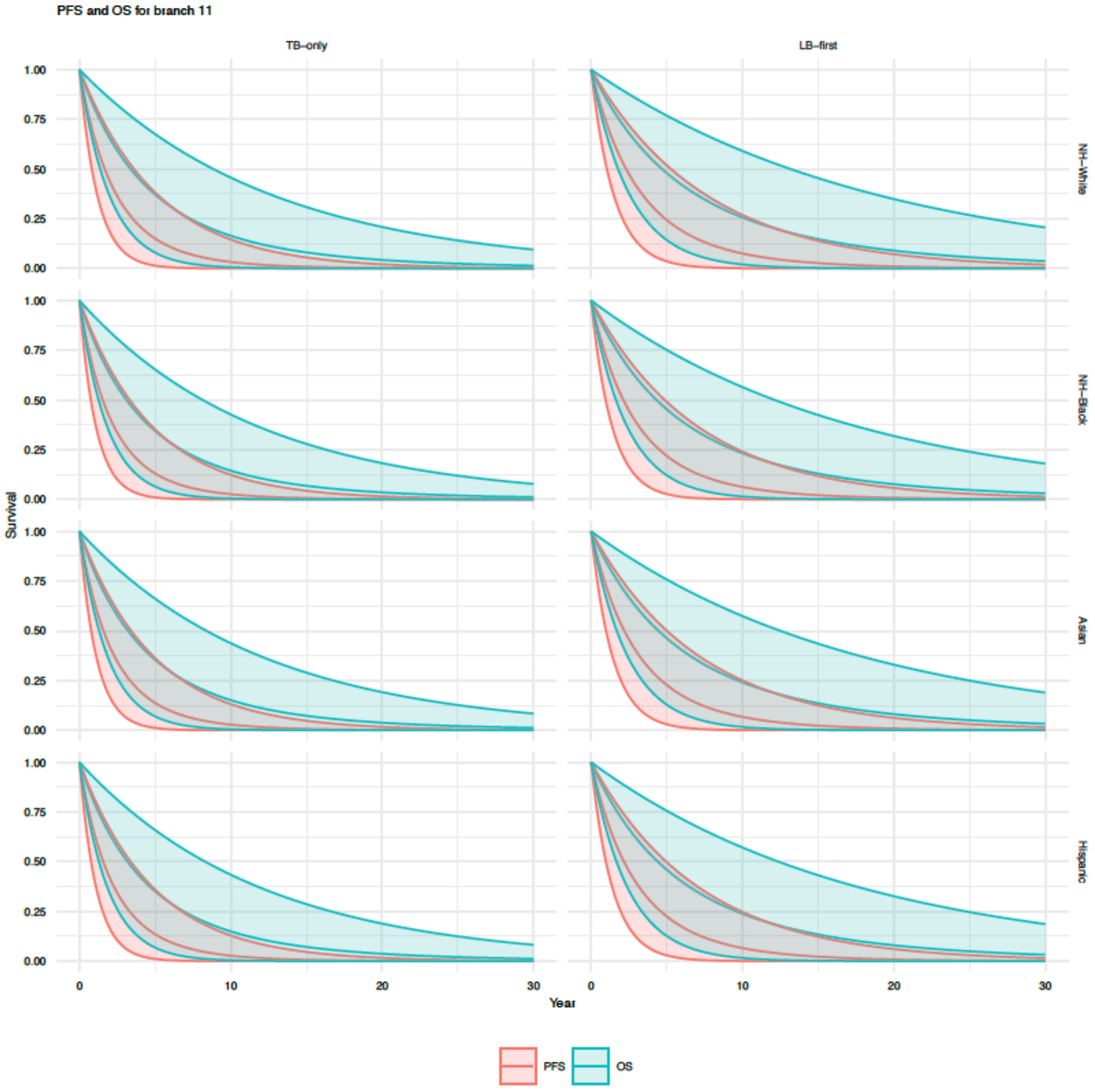

Figure S14: PFS and OS curves with treatment for false positive NTRK mutation with LB-first and TB-only strategies (Branch 12)

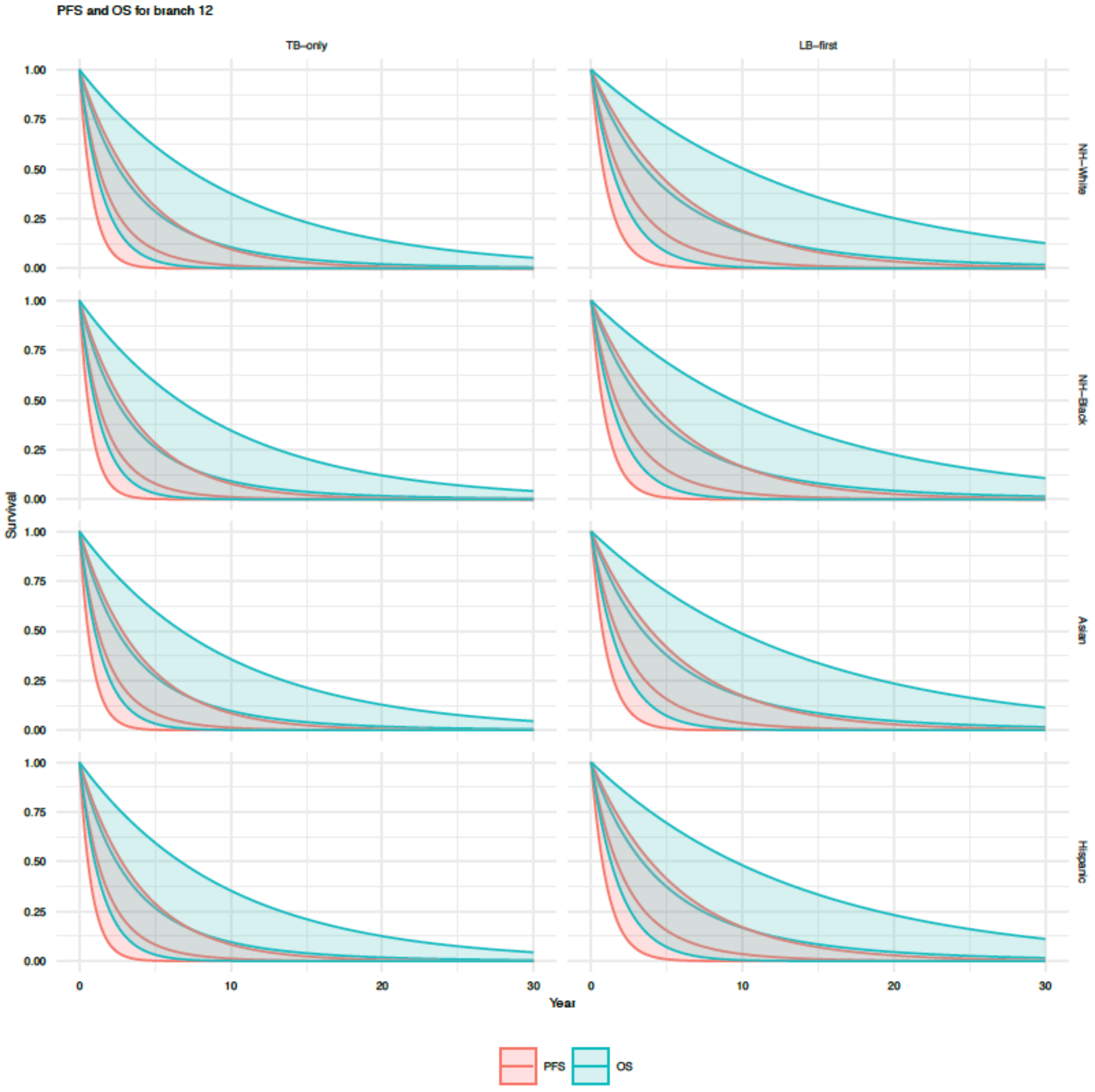

Figure S15: PFS and OS curves with treatment for true positive MET mutation with LB-first and TB-only strategies (Branch 13)

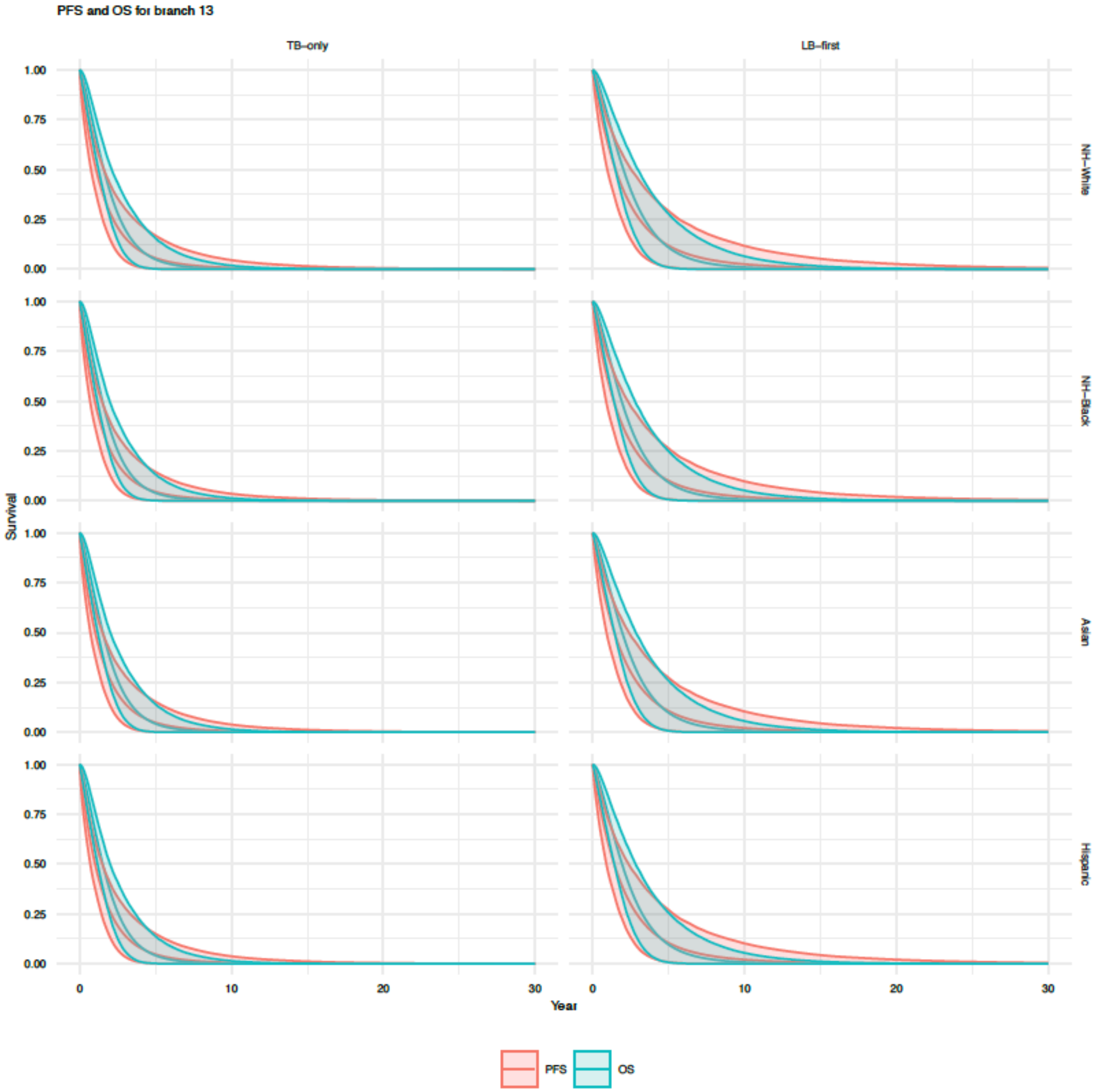

Figure S16: PFS and OS curves with treatment for false positive MET mutation with LB-first and TB-only strategies (Branch 14)

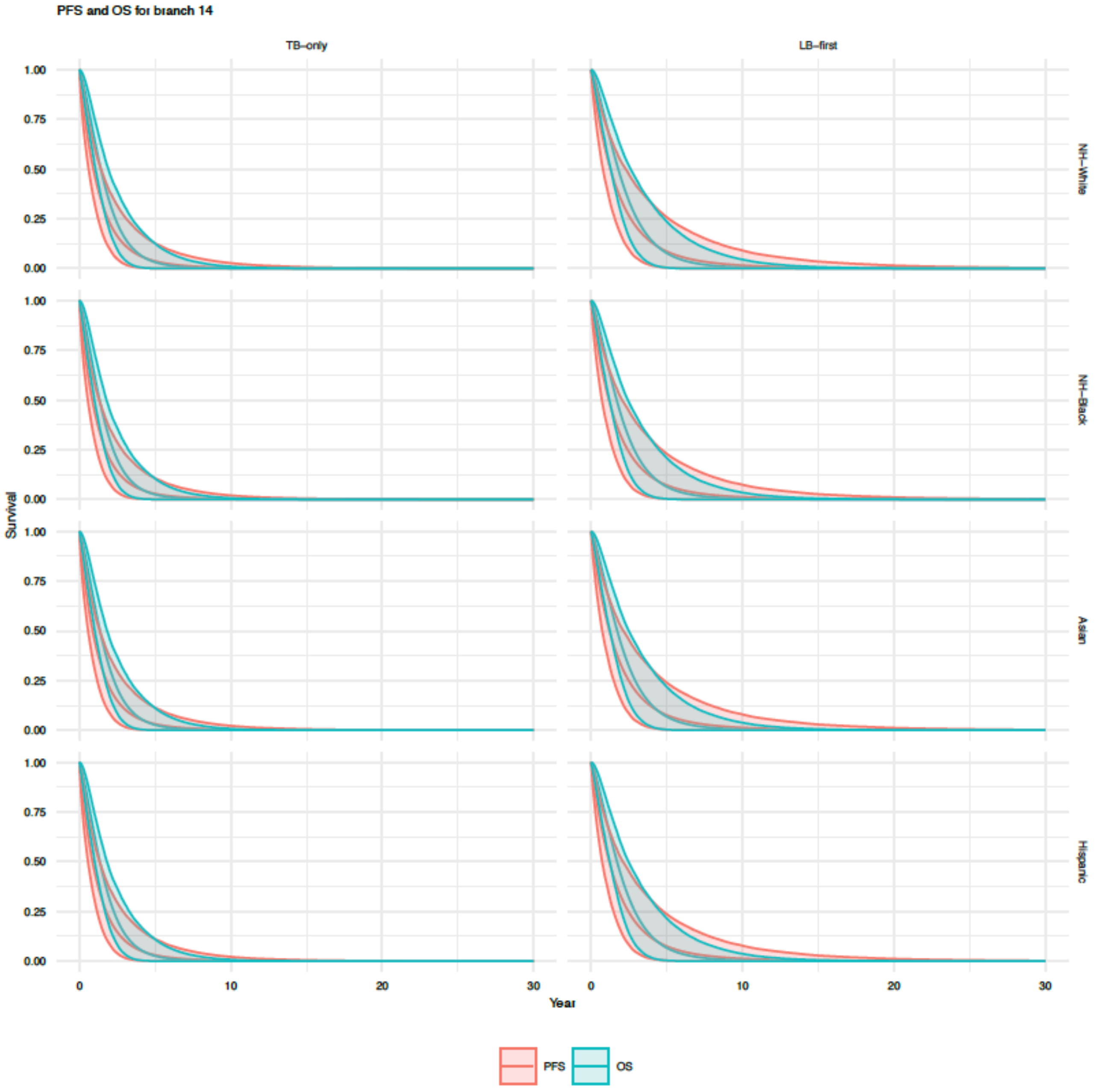

Figure S17: PFS and OS curves with treatment for true positive RET mutation with LB-first and TB-only strategies (Branch 15)

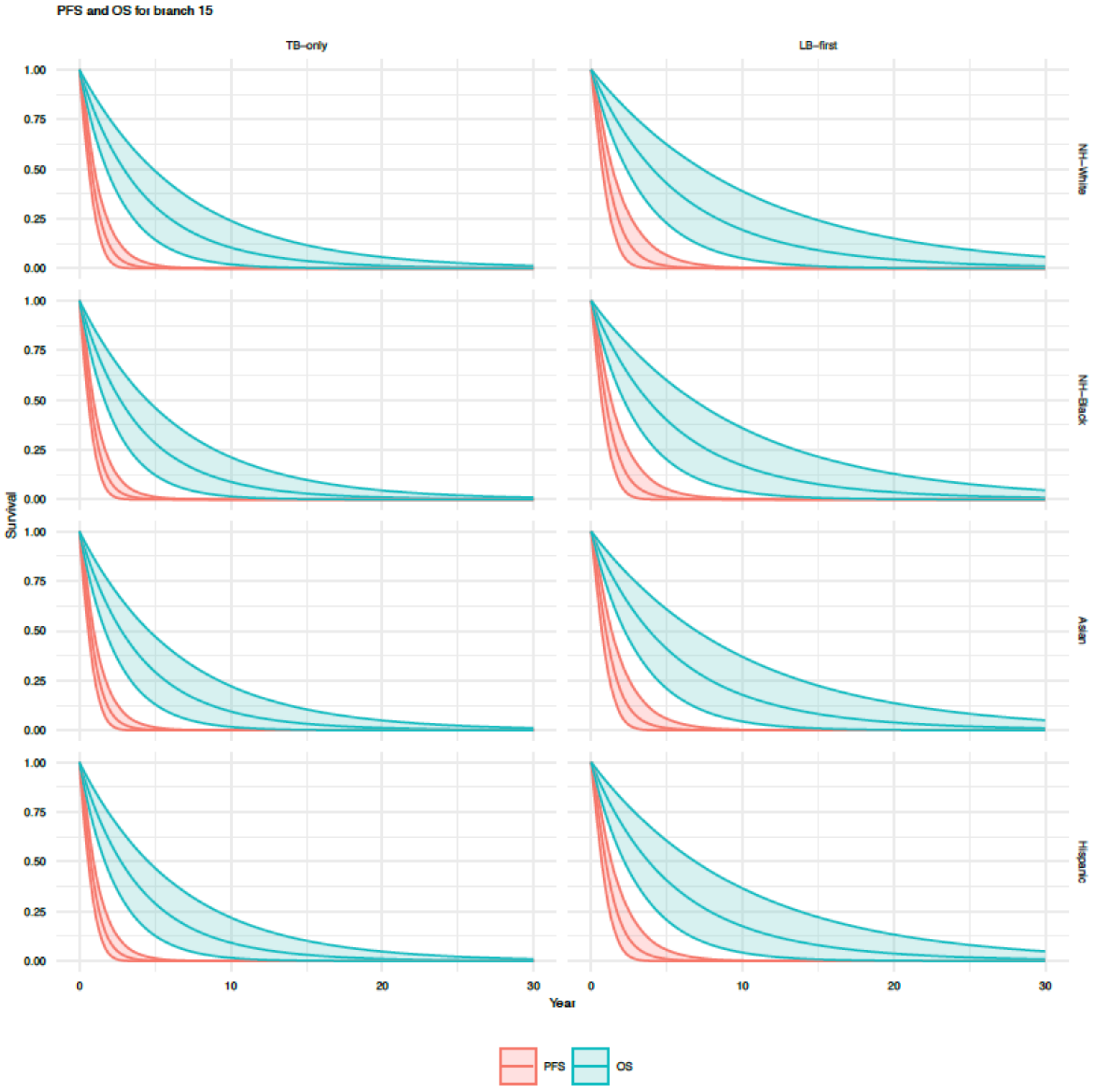

Figure S18: PFS and OS curves with treatment for false positive RET mutation with LB-first and TB-only strategies (Branch 16)

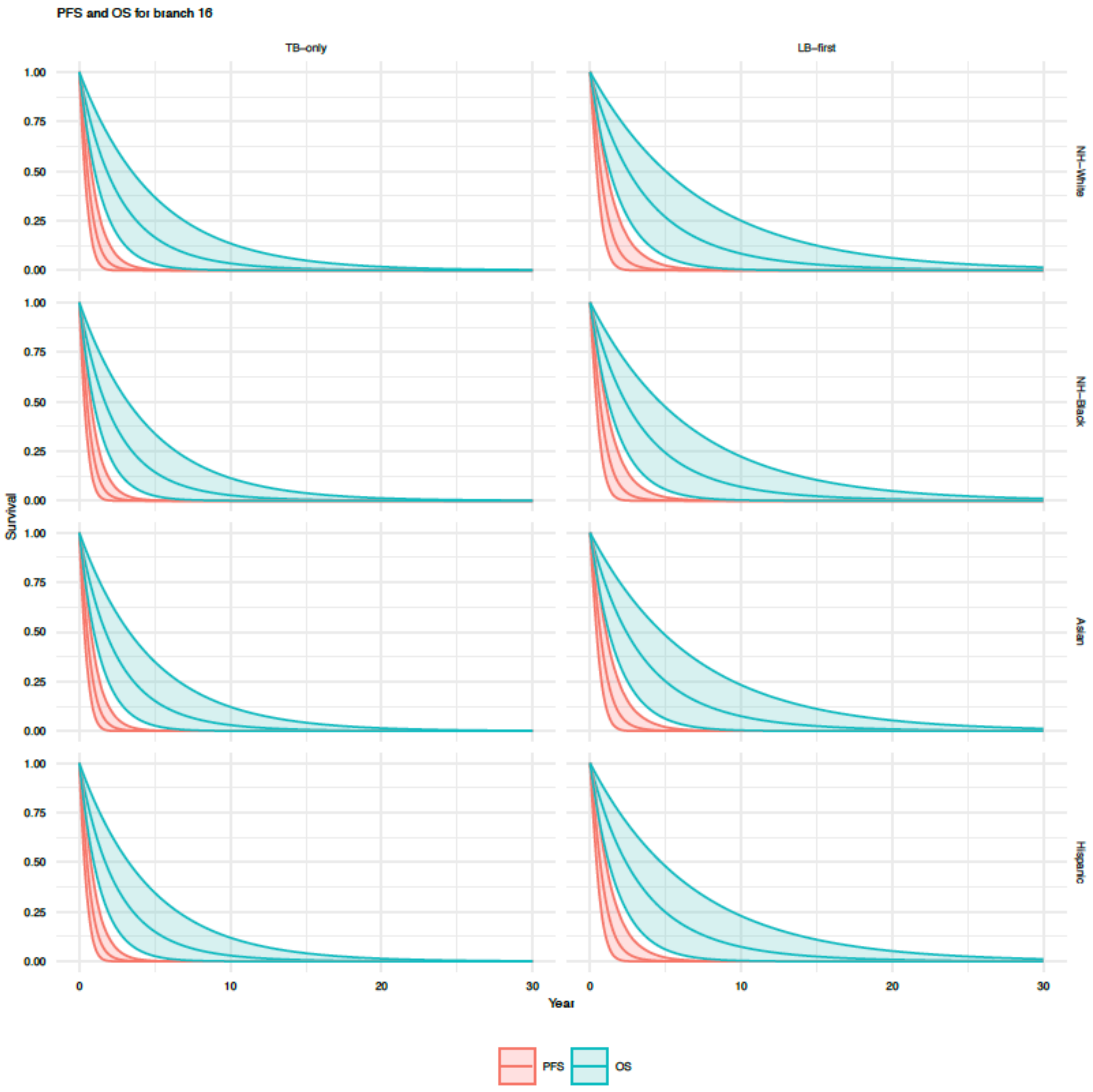

Branches 17 to 32 are the test results with a tissue sample following a negative LB result with the LB-first strategy. PFS and OS curves are the same as for branches 1-16 for the TB-only strategy and are therefore not presented here.

Figure S19: PFS and OS curves with treatment for true PD-L1 >50 positive result and false negative driver mutation with LB-first and TB-only strategies (Branch 33)

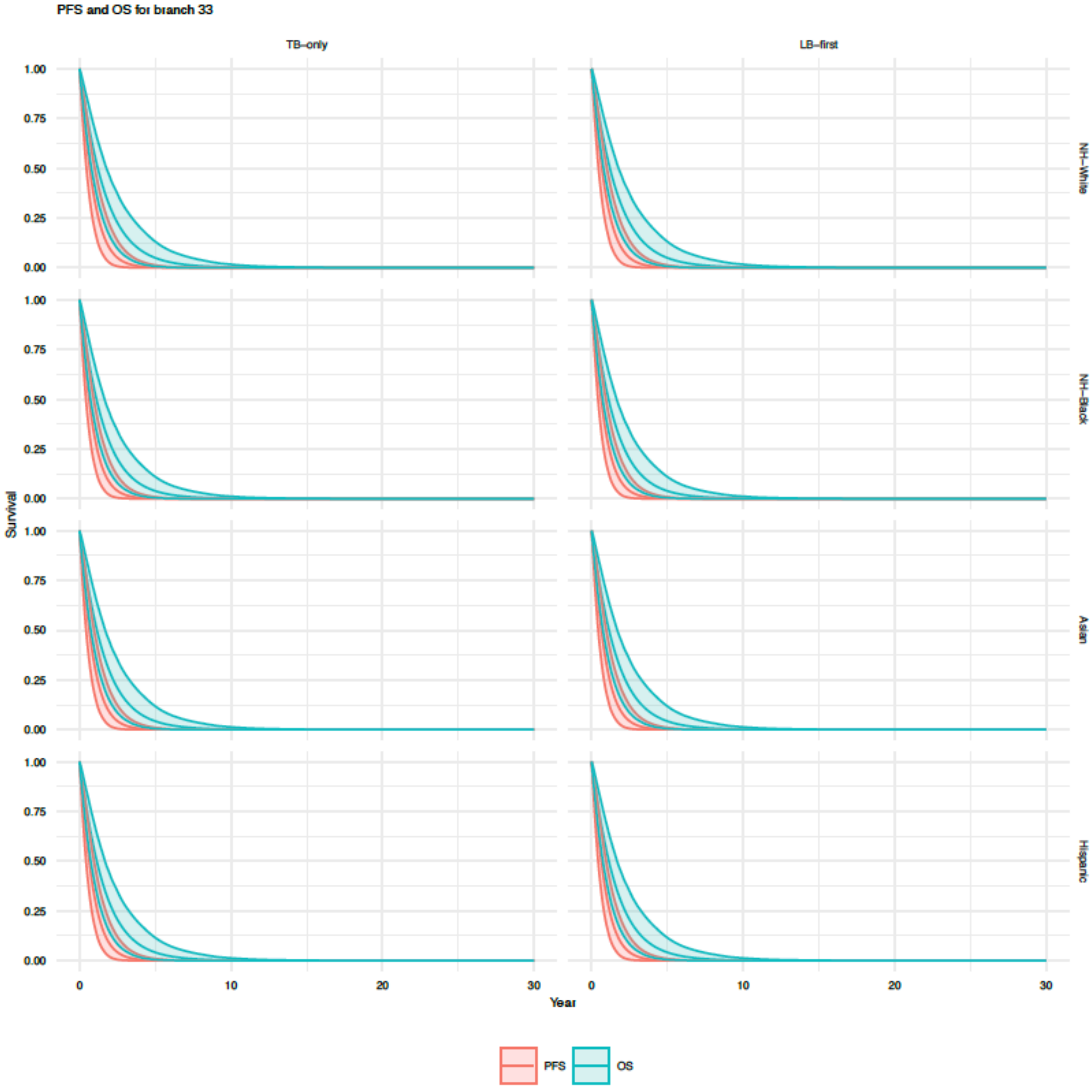

Figure S20: PFS and OS curves with treatment for true PD-L1 1-49 positive result and false negative driver mutation with LB-first and TB-only strategies (Branch 34)

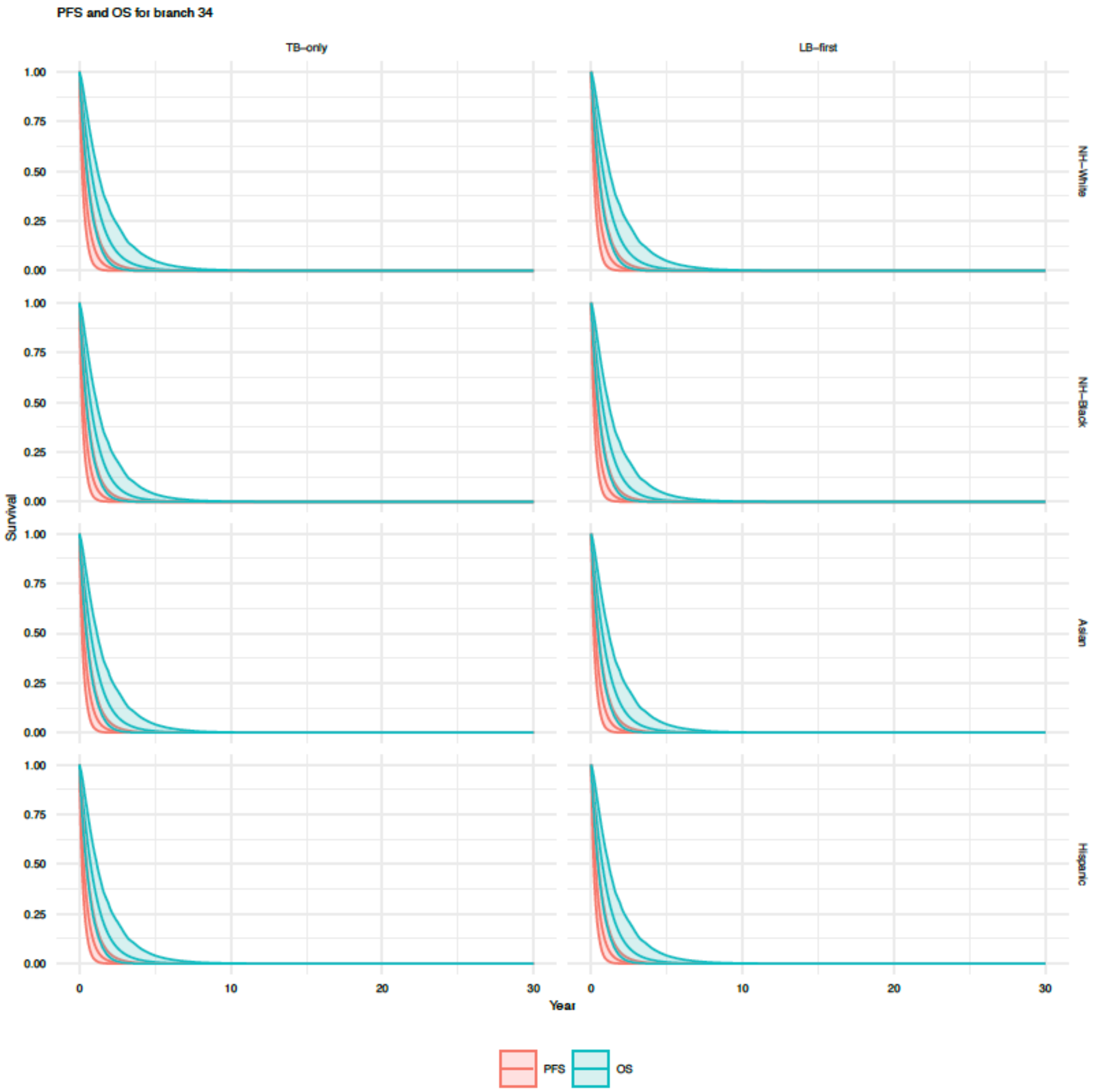

Figure S21: PFS and OS curves with treatment for false PD-L1 positive result and false negative driver mutation with LB-first and TB-only strategies (Branch 35)

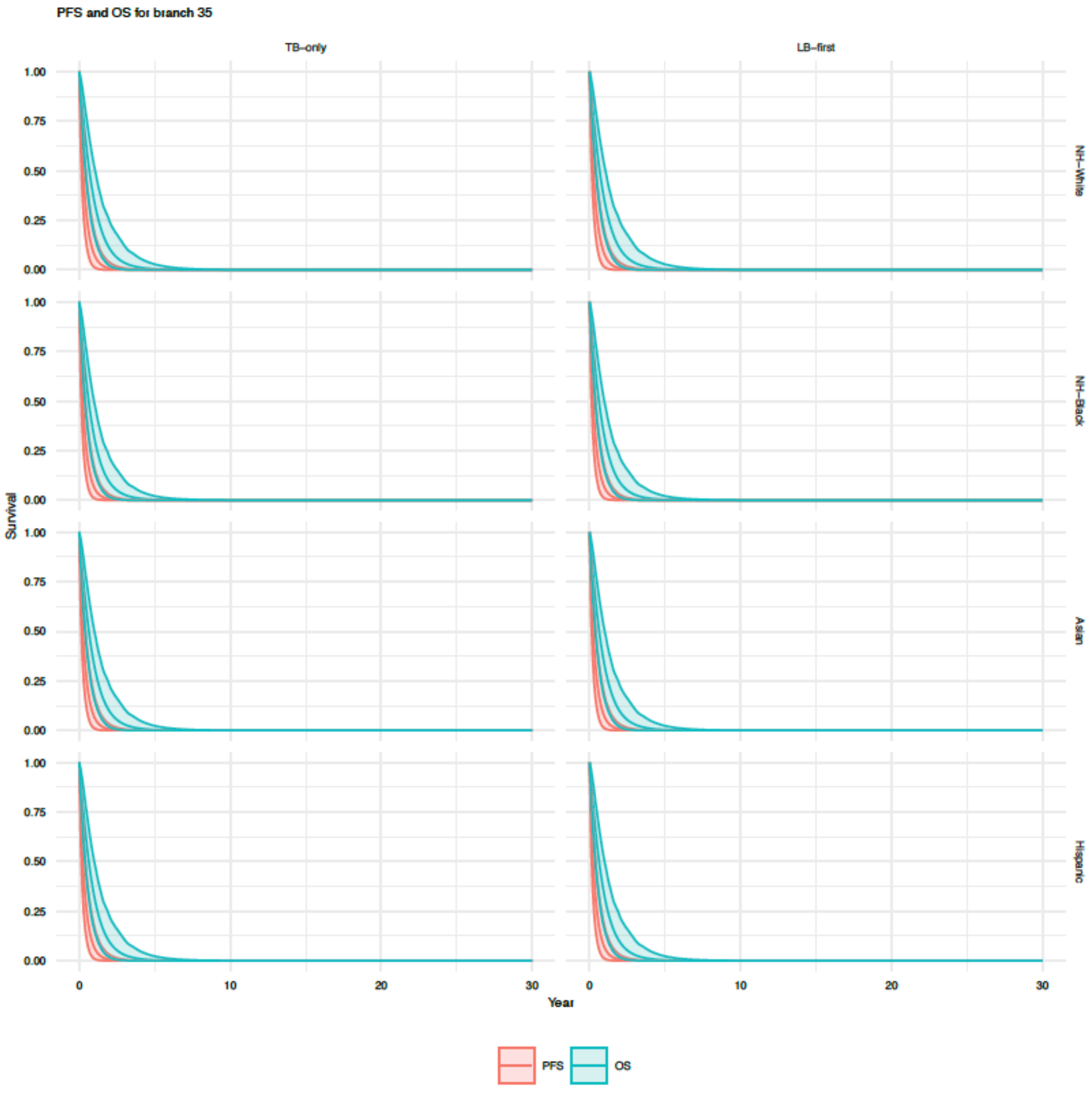

Figure S22: PFS and OS curves with treatment for false PD-L1 negative result and false negative driver mutation with LB-first and TB-only strategies (Branch 36)

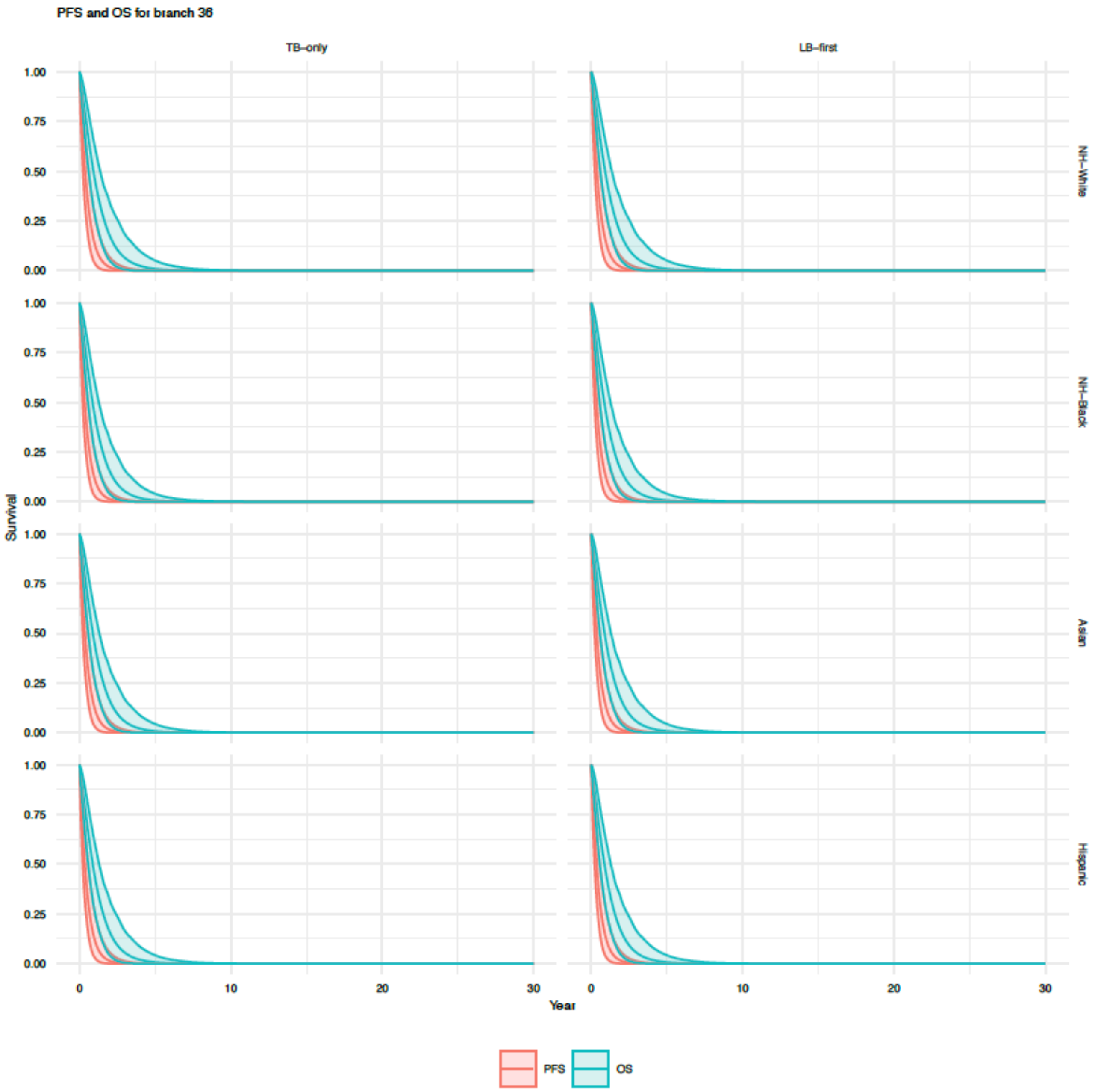

Figure S23: PFS and OS curves with treatment for true PD-L1 negative result and false negative driver mutation with LB-first and TB-only strategies (Branch 37)

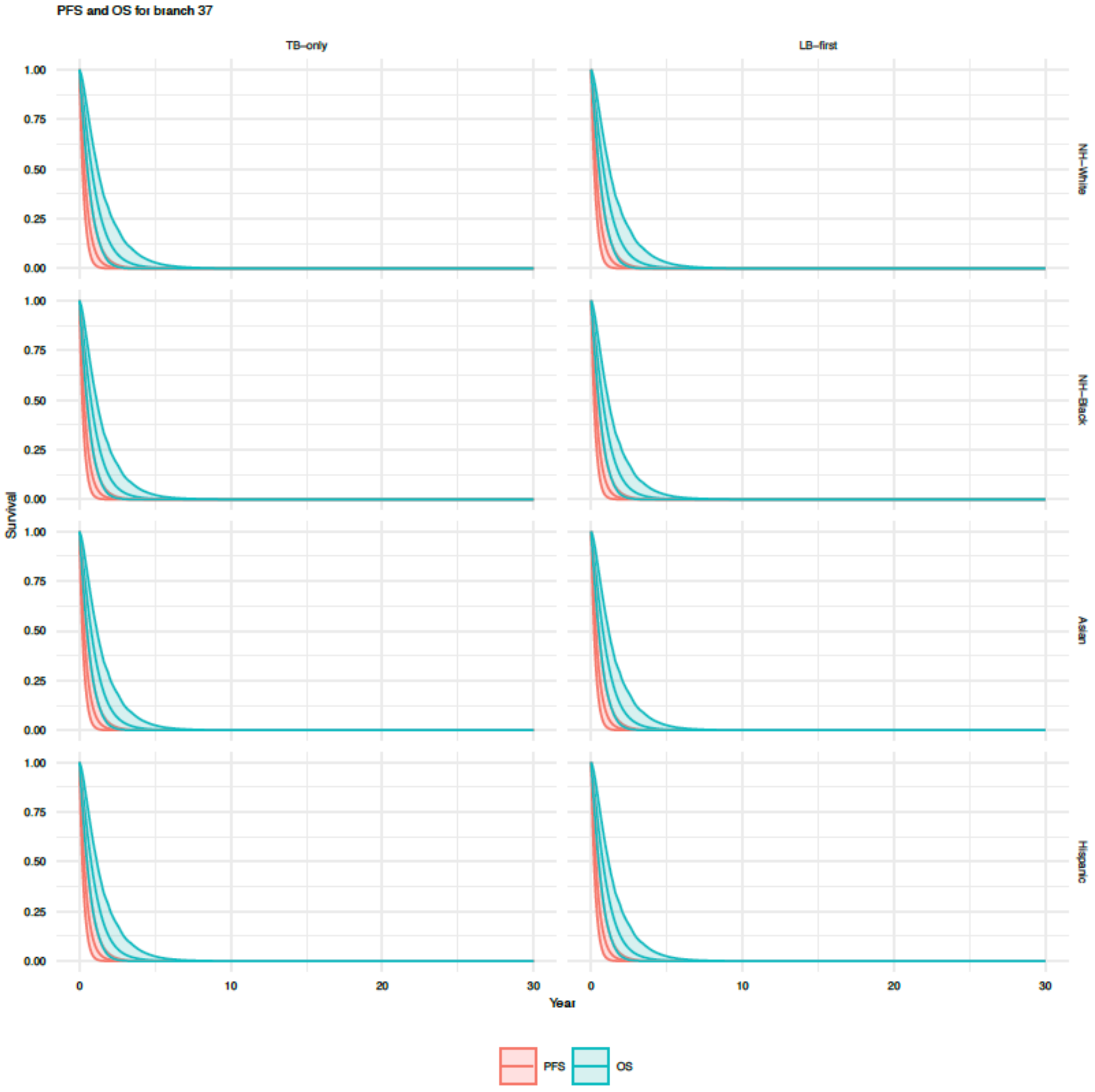

Figure S24: PFS and OS curves with treatment for true PD-L1 >50 positive result and true negative driver mutation with LB-first and TB-only strategies (Branch 38)

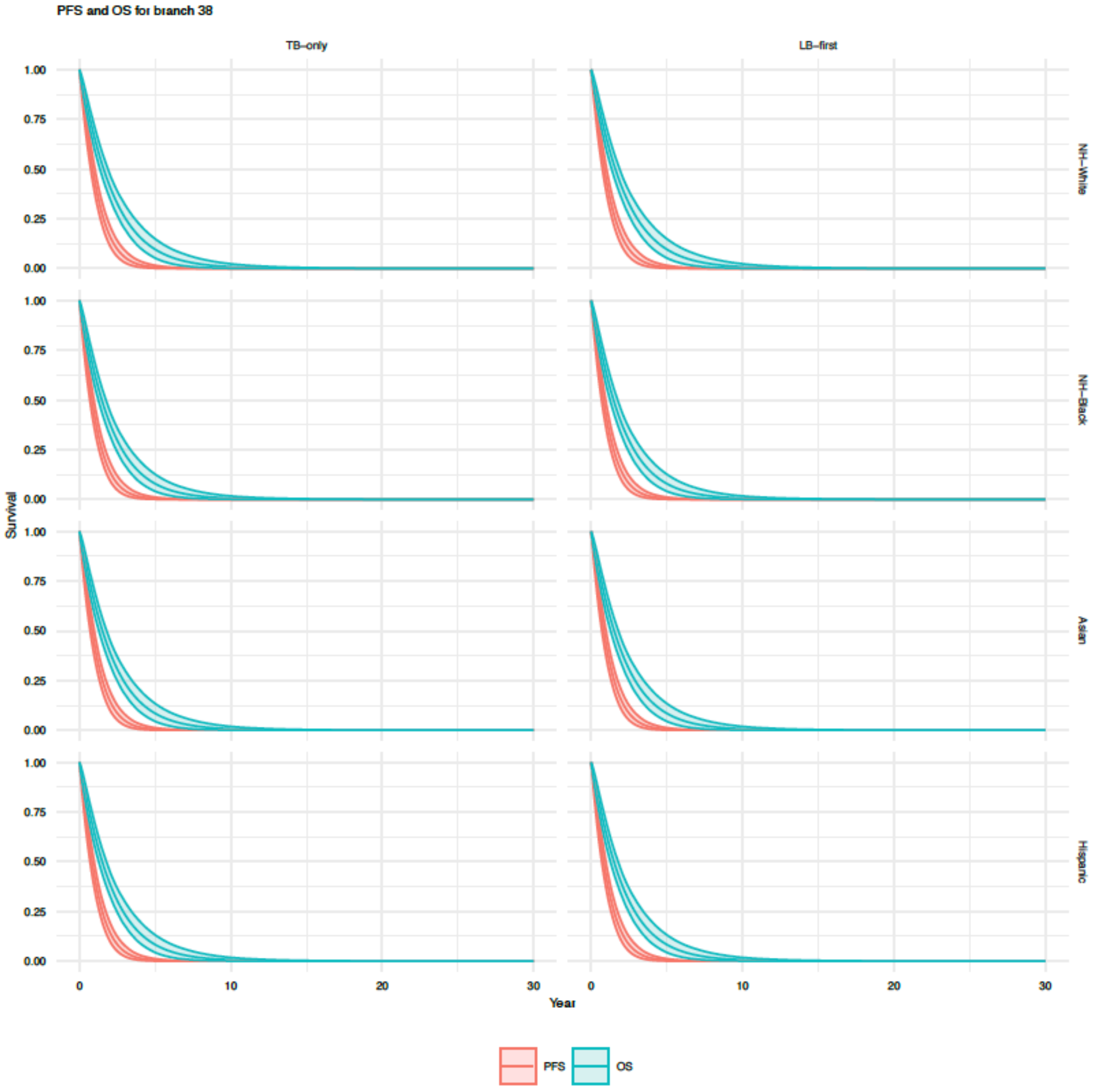

Figure S25: PFS and OS curves with treatment for true PD-L1 1-49 positive result and true negative driver mutation with LB-first and TB-only strategies (Branch 39)

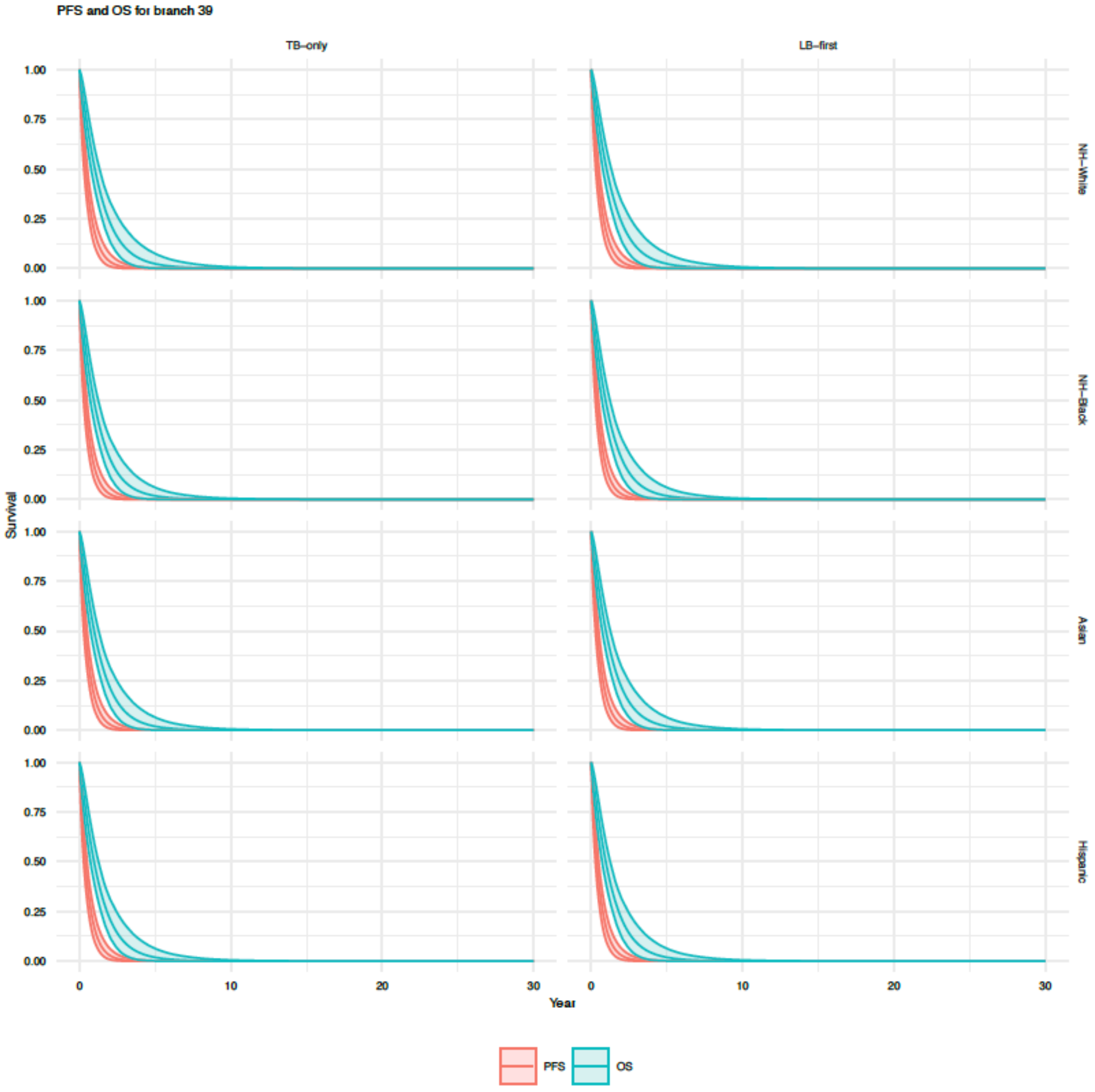

Figure S26: PFS and OS curves with treatment for false PD-L1 positive result and true negative driver mutation with LB-first and TB-only strategies (Branch 40)

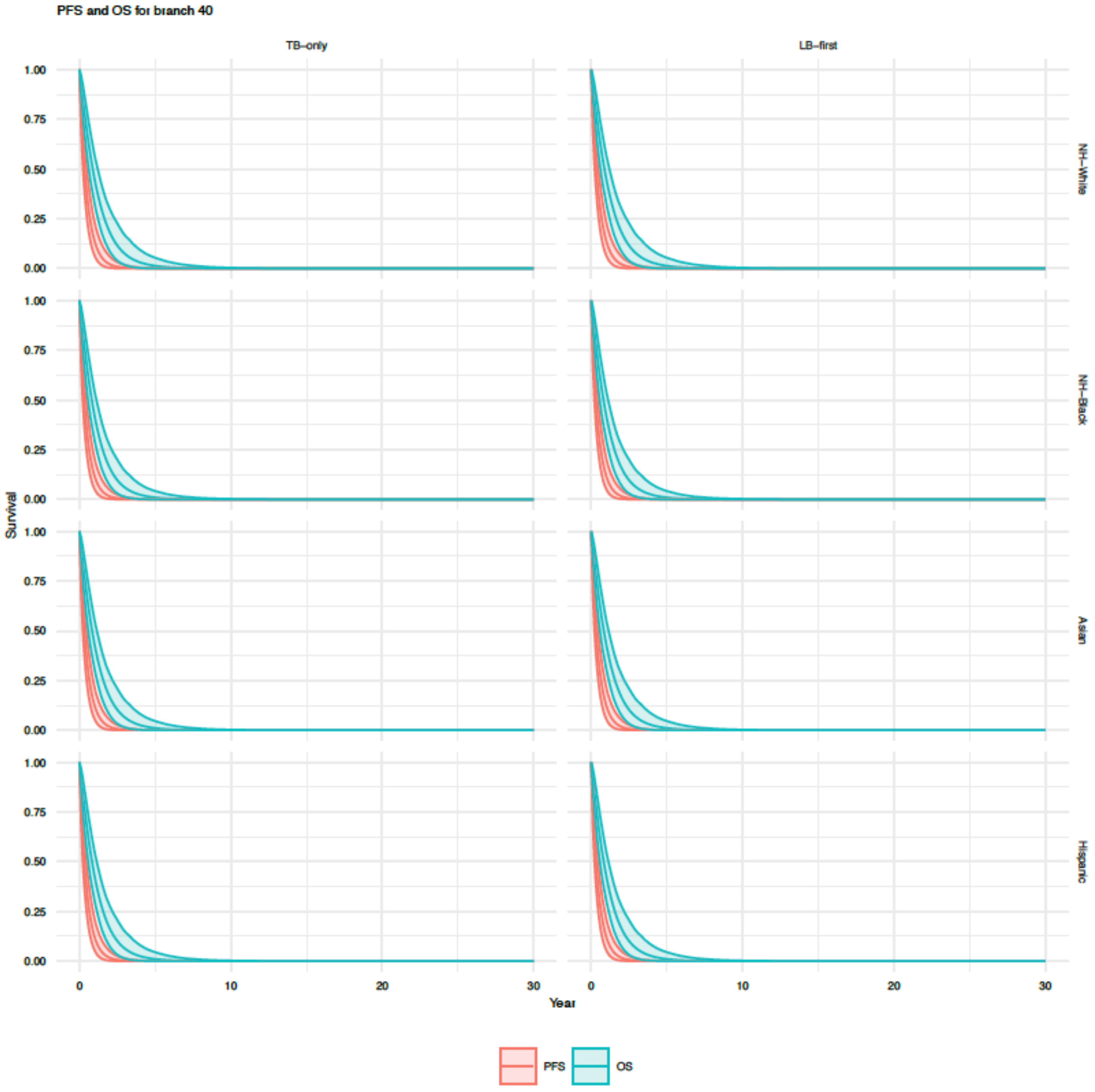

Figure S27: PFS and OS curves with treatment for false PD-L1 negative result and true negative driver mutation with LB-first and TB-only strategies (Branch 41)

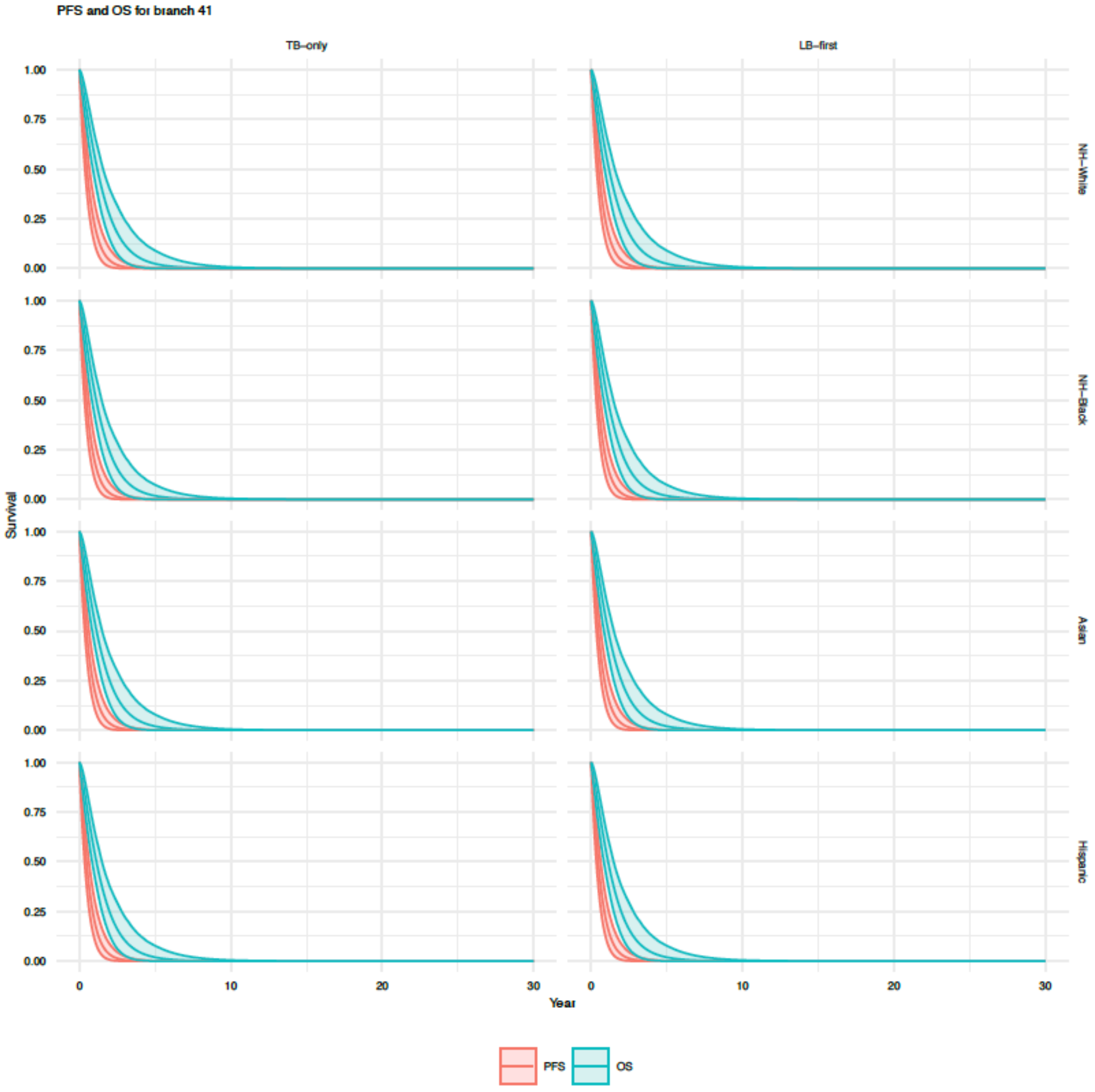

Figure S28: PFS and OS curves with treatment for true PD-L1 negative result and true negative driver mutation with LB-first and TB-only strategies (Branch 42)

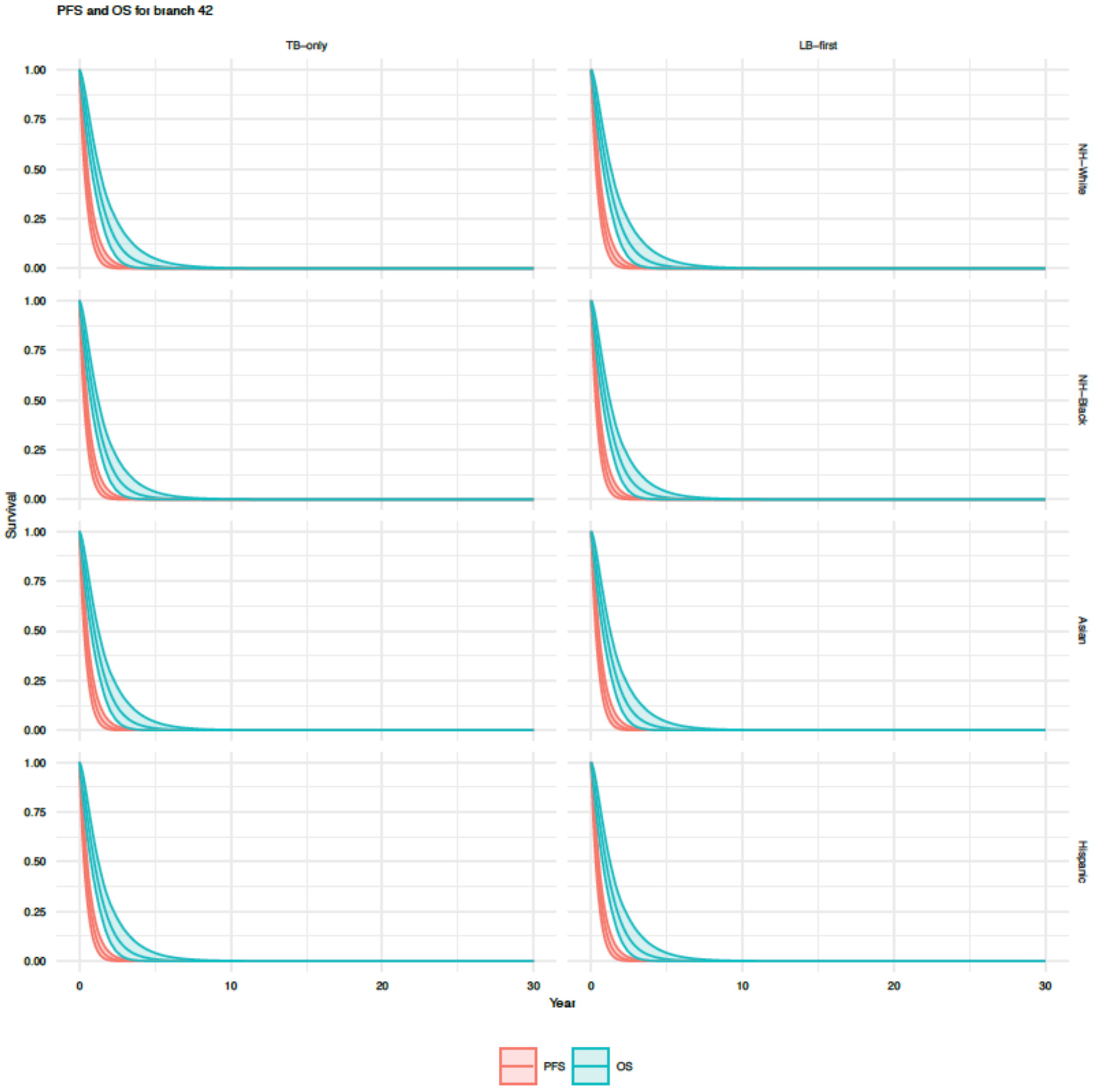

Model input: Resource use and unit costs associated with treatment and disease management

Table S1: Model input - Treatment costs with 1st line therapy according to driver mutation

Driver mutation	Regimen					Total drug costs (US$)						Total treatment costs including infusion if applicable (US$)		
		**Details**	**Drug**	**Dosage (mg)**	**Frequency**	**Strength (mg)**	**Pkg size**	**FSS pkg price**	**Year 1**	**Year 2**	**Year 2+**	**Year 1**	**Year 2**	**Year 2+**
EGFR	osimertinib	80 mg orally once daily until disease progression or unacceptable toxicity	osimertinib	80	daily	80	30	14641.00	**178132**	**178132**	**178132**	**178132**	**178132**	**178132**
KRAS G12c	pembro (+ carbo+ pem)	200 mg IV infusion over 30 minutes every 3 weeks or 400 mg IV over 30 minutes every 6 weeks, for up to 24 months or until disease progression or unacceptable toxicity. Give prior to pemetrexed 500 mg/m(2) IV and carboplatin AUC 5 mg/mL/min IV on day 1 of each 21-day cycle for 4 cycles, followed by pembrolizumab monotherapy with or without pemetrexed maintenance	pembrolizumab	200	every 3 weeks	100	1	5110.83	177662	177662	0			
			carboplatin	male 66, 70kg, cr 1mg/dl, auc5: 484.7; female 66, 60kg, cr 1mg/dl, auc5: 387.1	every 3 weeks for 4 cycles	450	1	36.07	143	0	0			
			pemetrexed	900	every 3 weeks	1000	1	322.47	5044	5044	5044			
	Total								**182849**	**182707**	**5044**	**185374**	**185231**	**7569**
ALK	alectinib, brigatinib, or lorlatinib	600 mg orally twice daily with food until disease progression	alectinib	600	twice daily	150	240	16187.78	196951	196951	196951			
		90 mg orally once daily for the first 7 days, then increase to 180 mg orally once daily until disease progression	brigatinib	90;180	once daily	90;180	7;30	3812.62;16339.49	198797	198797	198797			

Driver mutation	Regimen					Total drug costs (US$)						Total treatment costs including infusion if applicable (US$)		
		Details	Drug	Dosage (mg)	Frequency	Strength (mg)	Pkg size	FSS pkg price	Year 1	Year 2	Year 2+	Year 1	Year 2	Year 2+
		100 mg orally once daily with or without food; take at the same time each day until disease progression	lorlatinib	100	once daily	100	30	18137.97	220679	220679	220679			
	Average								**205476**	**205476**	**205476**	**205476**	**205476**	**205476**
ROS1	entrectinib	600 mg orally once daily with or without food until disease progression	entrectinib	600	once daily	200	90	18179.58	**221185**	**221185**	**221185**	**221185**	**221185**	**221185**
BRAF v600e	dabrafenib + trametinib	1) Usual dosage: 150 mg (two 75 mg capsules) orally twice daily in combination with trametinib 2 mg orally once daily; take 1 hour before or 2 hours after a meal.<br>2) Duration of therapy: Continue treatment until disease progression	dabrafenib	2*75	twice daily	75	120	12350.66	150266	150266	150266			
		1) Usual dosage: 2 mg orally once daily in combination with dabrafenib 150 mg orally every 12 hours. Take 1 hour before or 2 hours after a meal.<br>2) Duration of therapy: Continue treatment until disease progression	trametinib	2	once daily	2	30	13409.44	163148	163148	163148			
	Total								**313415**	**313415**	**313415**	**313415**	**313415**	**313415**
MET	tepotinib	450 mg orally once daily with food at approximately the same time every day until disease progression	tepotinib	450	once daily	225	60	15263.94	**185711**	**185711**	**185711**	**185711**	**185711**	**185711**

Driver mutation	Regimen					Total drug costs (US$)						Total treatment costs including infusion if applicable (US$)		
		Details	Drug	Dosage (mg)	Frequency	Strength (mg)	Pkg size	FSS pkg price	Year 1	Year 2	Year 2+	Year 1	Year 2	Year 2+
RET	pralsetinib	400 mg orally once daily until disease progression	pralsetinib	400	once daily	100	90	14629.32	**237320**	**237320**	**237320**	**237320**	**237320**	**237320**
NTRK	larotrectinib	(Body surface area 1 m(2) or greater) 100 mg orally twice daily with or without food until disease progression	larotrectinib	100	twice daily	100	60	33446.16	**406928**	**406928**	**406928**	**406928**	**406928**	**406928**
PDL1-50	pembrolizu mab	200 mg IV infusion over 30 minutes every 3 weeks or 400 mg IV over 30 minutes every 6 weeks; continue treatment for up to 24 months or until disease progression	pembrolizu mab	200	every 3 weeks	100	1	5110.83	**177662**	**177662**	**0**	**180187**	**180187**	**0**
PDL1-1-49	adeno, large, rare: pembo+ carbo+ pem	200 mg IV infusion over 30 minutes every 3 weeks or 400 mg IV over 30 minutes every 6 weeks, for up to 24 months or until disease progression or unacceptable toxicity. Give prior to pemetrexed 500 mg/m(2) IV and carboplatin AUC 5 mg/mL/min IV on day 1 of each 21-day cycle for 4 cycles, followed by pembrolizumab monotherapy with or without pemetrexed maintenance	pembrolizu mab	200	every 3 weeks	100	1	5110.83	177662	177662	0			
			carboplatin	male 66, 70kg, cr 1mg/dl, auc5: 484.7; female 66, 60kg, cr 1mg/dl, auc5: 387.1	every 3 weeks for 4 cycles	450	1	36.07	143	0	0			
			pemetrexed	900	every 3 weeks	1000	1	322.47	5044	5044	5044			
	Total								**182849**	**182707**	**5044**	**185374**	**185231**	**7569**
	squamous: pembro+ carbo+ pac	200 mg IV infusion over 30 minutes every 3 weeks or 400 mg IV over 30 minutes every 6	pembrolizu mab	200	every 3 weeks	100	1	5110.83	177662	177662	0			

Driver mutation	Regimen					Total drug costs (US$)						Total treatment costs including infusion if applicable (US$)		
		Details	Drug	Dosage (mg)	Frequency	Strength (mg)	Pkg size	FSS pkg price	Year 1	Year 2	Year 2+	Year 1	Year 2	Year 2+
		weeks and continue treatment for up to 24 months or until disease progression or unacceptable toxicity; give prior to carboplatin AUC 6 mg/mL/min on day 1 of each 21-day cycle and either paclitaxel 200 mg/m(2) on day 1 or nab-paclitaxel 100 mg/m(2) on days 1, 8, and 15 of each 21-day cycle for 4 cycles	carboplatin	male 66, 70kg, cr 1mg/dl, auc6: 581.7; female 66, 60kg, cr 1mg/dl, auc5: 464.5	every 3 weeks for 4 cycles	600	1	57.02	203	0	0			
			paclitaxel	360	3 times every 3 weeks for 4 cycles	100	1	778.39	33626	0	0			
	Total								**211492**	**177662**	**0**	**214017**	**180187**	**2525**
Wildtype	adeno, large, rare: pembo+ carbo+ pem	200 mg IV infusion over 30 minutes every 3 weeks or 400 mg IV over 30 minutes every 6 weeks, for up to 24 months or until disease progression or unacceptable toxicity. Give prior to pemetrexed 500 mg/m(2) IV and carboplatin AUC 5 mg/mL/min IV on day 1 of each 21-day cycle for 4 cycles, followed by pembrolizumab monotherapy with or without pemetrexed maintenance	pembrolizumab	200	every 3 weeks	100	1	5110.83	177662	177662	0			
			carboplatin	male 66, 70kg, cr 1mg/dl, auc5: 484.7; female 66, 60kg, cr 1mg/dl, auc5: 387.1	every 3 weeks for 4 cycles	450	1	36.07	143	0	0			
			pemetrexed	900	every 3 weeks	1000	1	322.47	5044	5044	5044			
	Total								**182849**	**182707**	**5044**	**185374**	**185231**	**7569**
	squamous: pembro+ carbo+ pac	200 mg IV infusion over 30 minutes every 3 weeks or 400 mg IV over 30 minutes every 6	pembrolizumab	200	every 3 weeks	100	1	5110.83	177662	177662	0			

Driver mutation	Regimen					Total drug costs (US$)						Total treatment costs including infusion if applicable (US$)		
		**Details**	**Drug**	**Dosage (mg)**	**Frequency**	**Strength (mg)**	**Pkg size**	**FSS pkg price**	**Year 1**	**Year 2**	**Year 2+**	**Year 1**	**Year 2**	**Year 2+**
		weeks and continue treatment for up to 24 months or until disease progression or unacceptable toxicity; give prior to carboplatin AUC 6 mg/mL/min on day 1 of each 21-day cycle and either paclitaxel 200 mg/m(2) on day 1 or nab-paclitaxel 100 mg/m(2) on days 1, 8, and 15 of each 21-day cycle for 4 cycles	carboplatin	male 66, 70kg, cr 1mg/dl, auc6: 581.7; female 66, 60kg, cr 1mg/dl, auc5: 464.5	every 3 weeks for 4 cycles	600	1	57.02	203	0	0			
			paclitaxel	360	3 times every 3 weeks for 4 cycles	100	1	778.39	33626	0	0			
	Total								**211492**	**177662**	**0**	**214017**	**180187**	**2525**

Table S2: Model input - Treatment costs with 2nd line therapy according to initial driver mutation

Driver mutation	Regimen					Drug costs (US$)				Total treatment costs including infusion if applicable (US$)
		Details	Drug	Dosage (mg)	Frequency	Strength (mg)	Pkg size	FSS pkg price	Per year	Per year
EGFR	afatinib+ cetuximab	1) Usual dosage: 40 mg orally once daily, at least 1 hour before or 2 hours after a meal [5]. 2) Duration: Continue until disease progression	afatinib	40	daily	40	30	9063.04	110267	
		cetuximab 500 mg/m2 i.v. every 2 weeks	cetuximab	900	every 2 weeks	100	1	660.58	155000	
	Total								**265267**	**269055**
KRAS G12c	sotorasib	960 mg (eight 120-mg tablets) orally once daily at the same time each day until disease progression	sotorasib	960	once daily	120	240	18703.83	**227563**	**227563**
ALK	lorlatinib	100 mg orally once daily with or without food; take at the same time each day until disease progression	lorlatinib	100	once daily	100	30	18137.97	220679	
	lorlatinib	100 mg orally once daily with or without food; take at the same time each day until disease progression	lorlatinib	100	once daily	100	30	18137.97	220679	
	pembro + carbo+ pem	200 mg IV infusion over 30 minutes every 3 weeks or 400 mg IV over 30 minutes every 6 weeks, for up to 24 months or until disease progression or unacceptable toxicity. Give prior to pemetrexed 500 mg/m(2) IV and carboplatin AUC 5 mg/mL/min IV on day 1 of each 21-day cycle for 4 cycles, followed by pembrolizumab monotherapy with or without pemetrexed maintenance	pembrolizumab	200	every 3 weeks	100	1	5110.83	177662	
			carboplatin	male 66, 70kg, cr 1mg/dl, auc5: 484.7; female 66, 60kg, cr 1mg/dl, auc5: 387.1	every 3 weeks for 4 cycles	450	1	36.07	143	
			pemetrexed	900	every 3 weeks	1000	1	322.47	5044	
	Total								182849	
	Average								**208069**	**210594**
ROS1	lorlatinib	100 mg orally once daily with or without food; take at the same time each day until disease progression		100	once daily	100	30	18137.97	**220679**	**220679**

Driver mutation	Regimen					Drug costs (US$)				Total treatment costs including infusion if applicable (US$)
		Details	Drug	Dosage (mg)	Frequency	Strength (mg)	Pkg size	FSS pkg price	Per year	Per year
BRAF v600e; MET, RET, NTRK	pembro + carbo+ pem	200 mg IV infusion over 30 minutes every 3 weeks or 400 mg IV over 30 minutes every 6 weeks, for up to 24 months or until disease progression or unacceptable toxicity. Give prior to pemetrexed 500 mg/m(2) IV and carboplatin AUC 5 mg/mL/min IV on day 1 of each 21-day cycle for 4 cycles, followed by pembrolizumab monotherapy with or without pemetrexed maintenance	pembrolizumab	200	every 3 weeks	100	1	5110.83	177662	
			carboplatin	male 66, 70kg, cr 1mg/dl, auc5: 484.7; female 66, 60kg, cr 1mg/dl, auc5: 387.1	every 3 weeks for 4 cycles	450	1	36.07	143	
			pemetrexed	900	every 3 weeks	1000	1	322.47	5044	
	Total								**182849**	**185374**
PD-L1 or wild type	docetaxel	75 mg/m(2) or 60 mg/m(2) IV infusion every 21 days until disease progression or unacceptable toxicity	docetaxel	135	every 3 weeks	20	1	12.66	1485	**4045**

Abbreviations: CT = computed tomography; ED = emergency department; GP = general practitioner; MRI = magnetic resonance imaging

Table S3: Model input – Unit costs associated with disease management

	HCPCS Code	Medicare rate (2022. US$)	Commercial rate (2022; inflated from Stargardter, US$)	Source
GP visit	99212	58.95	106.17	CMS Physician Fee Schedule (2022 - accessed Jan 29, 2023) or inflated from Stargardter et al. (2021) using US CPI: Medical Care Services from Dec 2020 to Dec 2022
Specialist visit	99214	133.21	220.34	
Nurse visit	99211	24.34	65.09	
Physiotherapist	97110	30.95	68.82	
CT scan	71250	147.11	1,525.31	
MRI	71550	386.97	3,640.16	
Respiratory surgical procedure	-	28,537.63	26,803.01	
Ultrasound	76604	62.04	339.85	
X-ray	71048	49.43	136.05	
Radiotherapy	77750	386.97	338.25	
Unscheduled hospitalization day	-	3,495.66	3,731.26	
ICU visit	Included in hospital stay cost			
ED visit	-	2,524.85	2,524.85	
Home care services	-	139.79	254.49	

Abbreviations: CT = computed tomography; ED = emergency department; GP = general practitioner; MRI = magnetic resonance imaging

Table S4: Model input – Resource use and costs associated with disease management

	Resource use		Medicare (2022, US$)		Commercial (2022, US$)	
	**Pre-progression** (per month)	**Post-progression** (per month)	**Pre-progression** (per month)	**Post-progression** (per month)	**Pre-progression** (per month)	**Post-progression** (per month)
GP visit	0.05	3.05	2.95	179.80	5.31	323.82
Specialist visit	0.5	1	66.61	133.21	$110.17	220.34
Nurse visit	0.13	0.12	3.16	2.92	8.46	7.81
Physiotherapist	0.002	0	0.06	0.00	0.14	0.00
CT scan	0.5	1	73.56	147.11	762.66	1,525.31
MRI	0.5	1	193.49	386.97	1,820.08	3,640.16
Respiratory surgical procedure	0.008	0	228.30	0.00	214.42	0.00
Ultrasound	0.5	1	31.02	62.04	169.92	339.85
X-ray	0.5	1	24.72	49.43	68.02	136.05
Radiotherapy	0.003	0	1.16	0.00	1.01	0.00
Unscheduled hospitalization day	0.07	1.72	244.70	6,012.53	261.19	6,417.76
ICU visit	0.03	0.13				
ED visit	0.06	0.02	151.49	50.50	151.49	50.50
Home care services	0	0.57	0.00	79.68	0.00	145.06
Total			**1,021.20**	**7,104.19**	**3,572.88**	**12,806.65**

Abbreviations: CT = computed tomography; ED = emergency department; GP = general practitioner; MRI = magnetic resonance imaging

Calculating net health benefit

Multiplying the QALYs and costs per patient with treatment *k* in subgroup *g* with the lifetime risk of aNSCLC without sufficient tissue for molecular testing among corresponding individuals of the general population, i_g, we get the expected QALYs and costs expressed per subgroup member of the general population. Assuming equally distributed opportunity costs across members of the general population, the corresponding NHB per member of the general population is:

$$NHB_{gk} = i_g QALY_{gk} - \frac{\sum_{g=1}^{G} w_g i_g cost_{gk}}{\lambda}$$

with w_g the proportion of subpopulation *g* (e.g., NH-White, NH-Black, Asian, and Hispanic) in the general population, and λ the opportunity cost threshold.

The incremental NHB with LB-first relative to TB-only per member of the general population is:

$$\mathrm{i}NHB_g = NHB_{g2} - NHB_{g1}$$

with the distribution of quality-adjusted life expectancy at birth (QALE) without LB-first defined as $QALE_{g1}$, the QALE with LB-first is defined as:

$$QALE_{g2} = QALE_{g1} + \mathrm{i}NHB_g$$

Atkinson and Kolm inequality equations

The Atkinson inequality index for strategy k (LB-first or TB-only) is defined as:

$$A_k^{\varepsilon} = 1 - \left[\sum_g w_g \left(\frac{Q_{gk}}{\overline{Q_k}}\right)^{1-\varepsilon}\right]^{\frac{1}{1-\varepsilon}}$$

where w_i is the proportion of race and ethnicity subgroup g in the target patient population or general population, Q_{gk} are the expected QALYs per patient or QALE per member of the general population for subgroup g with strategy k. $\overline{Q_k}$ are the expected QALYs for the target patient population overall or QALE for the general population overall, and ε is the inequality aversion parameter that quantifies the concern for relative inequality. The corresponding equally distributed equivalent (EDE) levels of health (QALYs or QALE) with strategy k is defined as $Q_{EDE,k}^{A^{\varepsilon}} = (1 - A_k^{\varepsilon})\overline{Q_k}$

The Kolm inequality index for strategy k is defined as: $K_k^{\alpha} = \frac{1}{\alpha} log\left(\sum_i w_g e^{\alpha[\overline{Q_k} - Q_{gk}]}\right)$ where α the inequality aversion parameter that quantifies the concern for absolute inequality. (The corresponding EDE levels of health with strategy k is defined as: $Q_{EDE,k}^{K^{\alpha}} = \overline{Q_k} - K_k^{\alpha}$)

Additional results

Table S5: Expected discounted QALYs per patient, discounted costs related to diagnostic workup, treatment, and disease management per patient, incremental net health benefit (iNHB) per 100,000 individuals of the general population factoring in equally distributed opportunity costs at a threshold of $150k, and QALE per member of the general population by race and ethnicity without and with LB-first.

Outcome	Group	Tissue biopsy only		Liquid biopsy first		Difference	
		mean	95% uncertainty interval	mean	95% uncertainty interval	mean	95% uncertainty interval
QALYs (per patient)	NH-White	1.38	(1.11; 1.74)	1.58	(1.23; 2.06)	0.20	(0.07; 0.38)
	NH-Black	1.38	(1.09; 1.75)	1.59	(1.22; 2.09)	0.21	(0.07; 0.41)
	Asian	1.67	(1.37; 2.06)	1.98	(1.56; 2.5)	0.31	(0.09; 0.61)
	Hispanic	1.46	(1.18; 1.84)	1.63	(1.28; 2.07)	0.17	(0.05; 0.35)
	All	1.41	(1.13; 1.77)	1.61	(1.26; 2.09)	0.21	(0.07; 0.39)
Costs (US$, diagnostic workup, treatment, and disease management, per patient)	NH-White	352,553	(289,470; 435,554)	407,620	(325,169; 517,676)		
	NH-Black	370,022	(295,305; 463,782)	431,625	(335,561; 553,211)		
	Asian	462,075	(382,793; 559,581)	549,955	(442,521; 686,573)		
	Hispanic	374,506	(302,941; 460,700)	425,852	(335,181; 539,679)		
	All	364,233	(299,721; 447,579)	421,863	(337,282; 532,310)	57,629	(20,389; 107,920)
Incremental NHB (in QALYs per 100,000 individuals of the general population)	NH-White					-80	(-153; -23)
	NH-Black					-67	(-136; -21)
	Asian					-48	(-110; -6)
	Hispanic					-142	(-269; -51)
	All					-88	(-166; -29)
		Pre LB-first distribution		*Post LB-first distribution*			
QALE (per individual general population)	NH-White	68.798		68.797	(68.796; 68.798)		
	NH-Black	65.446		65.446	(65.445; 65.446)		
	Asian	74.878		74.878	(74.877; 74.878)		
	Hispanic	71.762		71.761	(71.759; 71.762)		
	All	69.283		69.283	(69.282; 69.283)		

Table S6: Inequality metrics for expected QALYs per patient and expected QALE per individual of the general population factoring in equally distributed opportunity costs related to diagnostic workup, treatment, and disease management at a threshold of $150k and different degrees of inequality aversion.

Outcome	Metric	Inequality aversion	Tissue biopsy only		Liquid biopsy first		Difference*	
			mean	95% uncertainty interval	mean	95% uncertainty interval	mean	95% uncertainty interval
QALYs (per patient)	Atkinson	0.9	0.00134	(0.00048; 0.00256)	0.0017	(0.00063; 0.00328)	0.00036	(-0.00014; 0.00118)
		5	0.00627	(0.00234; 0.01184)	0.0076	(0.00303; 0.0143)	0.00134	(-0.00077; 0.00487)
		11 (base-case)	0.01109	(0.00439; 0.02149)	0.01291	(0.00542; 0.02404)	0.00182	(-0.00145; 0.00803)
		15	0.01337	(0.00535; 0.02654)	0.0153	(0.00657; 0.02966)	0.00193	(-0.00201; 0.01026)
	Kolm	0.025	0.00008	(0.00003; 0.00015)	0.00013	(0.00005; 0.00028)	0.00006	(0; 0.00017)
		0.1	0.00031	(0.00013; 0.00061)	0.00053	(0.0002; 0.00111)	0.00022	(0.00001; 0.00065)
		0.15 (base-case)	0.00046	(0.0002; 0.00091)	0.00080	(0.00029; 0.00166)	0.00033	(0.00002; 0.00097)
		0.3	0.00092	(0.00039; 0.0018)	0.00157	(0.00058; 0.00327)	0.00065	(0.00003; 0.0019)
QALE (per individual general population)	Atkinson	0.9	0.0004948	(0.0004948; 0.0004948)	0.0004948	(0.0004947; 0.0004948)	0.0000000	(-0.0000001; 0.0000000)
		5	0.0027011	(0.0027011; 0.0027011)	0.0027009	(0.0027006; 0.0027011)	-0.0000002	(-0.0000006; 0.0000000)
		11 (base-case)	0.0058032	(0.0058032; 0.0058032)	0.0058027	(0.0058019; 0.0058032)	-0.0000005	(-0.0000013; -0.0000001)
		15	0.0077995	(0.0077995; 0.0077995)	0.0077988	(0.0077977; 0.0077994)	-0.0000007	(-0.0000018; -0.0000001)

Outcome	Metric	Inequality aversion	Tissue biopsy only		Liquid biopsy first		Difference*	
			mean	95% uncertainty interval	mean	95% uncertainty interval	mean	95% uncertainty interval
	Kolm	0.025	0.0659460	(0.065946; 0.065946)	0.0659395	(0.0659311; 0.0659447)	-0.0000065	(-0.0000149; -0.0000013)
		0.1	0.2569327	(0.2569327; 0.2569327)	0.2569052	(0.2568691; 0.2569265)	-0.0000275	(-0.0000635; -0.0000061)
		0.15 (base-case)	0.3791431	(0.3791431; 0.3791431)	0.3791010	(0.3790459; 0.3791334)	-0.0000421	(-0.0000972; -0.0000097)
		0.3	0.7259447	(0.7259447; 0.7259447)	0.7258599	(0.7257466; 0.7259244)	-0.0000848	(-0.0001981; -0.0000203)

* difference: $Atkinson_{LB\text{-}first}$ – $Atkinson_{TB\text{-}only}$ or $Kolm_{LB\text{-}first}$ – $Kolm_{TB\text{-}only}$. A positive number implies an increase in inequality

Table S7: Estimated iNHB with LB-first per 100,000 individuals of the general population factoring in equally distributed opportunity costs at a threshold of $50k, $100k, $150k, $200k by race and ethnicity.

	Group	iNHB based on diagnostic workup costs		iNHB based on diagnostic workup, treatment and disease management costs	
		mean	95% uncertainty interval	mean	95% uncertainty interval
$50k/QALY	NH-White	77	(7; 174)	-459	(-865; -167)
	NH-Black	91	(10; 204)	-446	(-833; -159)
	Asian	109	(11; 254)	-427	(-808; -160)
	Hispanic	16	(-18; 67)	-521	(-982; -188)
	All	69	(4; 162)	-467	(-877; -169)
$100k/QALY	NH-White	93	(22; 190)	-175	(-331; -61)
	NH-Black	107	(27; 219)	-161	(-305; -59)
	Asian	126	(26; 269)	-143	(-268; -50)
	Hispanic	32	(-1; 84)	-236	(-450; -87)
	All	85	(20; 177)	-183	(-341; -66)
$150k/QALY	NH-White	99	(28; 195)	-80	(-153; -23)
	NH-Black	112	(32; 224)	-67	(-136; -21)
	Asian	131	(32; 273)	-48	(-110; -6)
	Hispanic	37	(4; 89)	-142	(-269; -51)
	All	91	(25; 182)	-88	(-166; -29)
$200k/QALY	NH-White	101	(30; 198)	-33	(-67; -6)
	NH-Black	115	(35; 227)	-19	(-60; 11)
	Asian	134	(34; 276)	0	(-39; 40)
	Hispanic	40	(7; 91)	-94	(-179; -34)
	All	93	(28; 184)	-41	(-78; -11)

Table S8: Inequality metrics for QALE per individual of the general population factoring in equally distributed opportunity costs related to diagnostic workup at threshold of $50k, $100k, $150k, $200k for different degrees of inequality aversion.

Outcome	Metric	Inequality aversion	Tissue biopsy only		Liquid biopsy first		Difference*		% Difference	
			mean	95% uncertainty interval	mean	95% uncertainty interval	mean	95% uncertainty interval	mean	95% uncertainty interval
QALE at $50k	Atkinson	0.9	0.00049	(0.00049; 0.00049)	0.00049	(0.00049; 0.00049)	-0.0000001	(-0.0000001; 0.0000000)	-0.012	(-0.027; -0.003)
		5	0.0027	(0.0027; 0.0027)	0.0027	(0.0027; 0.0027)	-0.0000003	(-0.0000008; -0.0000001)	-0.013	(-0.028; -0.003)
		11	0.0058	(0.0058; 0.0058)	0.0058	(0.0058; 0.0058)	-0.0000008	(-0.0000017; -0.0000002)	-0.013	(-0.030; -0.003)
		15	0.0078	(0.0078; 0.0078)	0.0078	(0.0078; 0.0078)	-0.0000011	(-0.0000024; -0.0000003)	-0.014	(-0.031; -0.003)
	Kolm	0.025	0.06595	(0.06595; 0.06595)	0.06594	(0.06593; 0.06594)	-0.0000065	(-0.0000149; -0.0000013)	-0.010	(-0.023; -0.002)
		0.1	0.25693	(0.25693; 0.25693)	0.25691	(0.25687; 0.25693)	-0.0000275	(-0.0000635; -0.0000061)	-0.011	(-0.025; -0.002)
		0.15	0.37914	(0.37914; 0.37914)	0.3791	(0.37905; 0.37913)	-0.0000421	(-0.0000972; -0.0000097)	-0.011	(-0.026; -0.003)
		0.3	0.72594	(0.72594; 0.72594)	0.72586	(0.72575; 0.72592)	-0.0000848	(-0.0001981; -0.0000203)	-0.012	(-0.027; -0.003)
QALE at $100k	Atkinson	0.9	0.00049	(0.00049; 0.00049)	0.00049	(0.00049; 0.00049)	-0.0000001	(-0.0000001; 0.0000000)	-0.012	(-0.027; -0.003)
		5	0.0027	(0.0027; 0.0027)	0.0027	(0.0027; 0.0027)	-0.0000004	(-0.0000008; -0.0000001)	-0.013	(-0.028; -0.004)
		11	0.0058	(0.0058; 0.0058)	0.0058	(0.0058; 0.0058)	-0.0000008	(-0.0000017; -0.0000002)	-0.014	(-0.030; -0.004)
		15	0.0078	(0.0078; 0.0078)	0.0078	(0.0078; 0.0078)	-0.0000011	(-0.0000024; -0.0000003)	-0.014	(-0.031; -0.004)

	Kolm	0.025	0.06595	(0.06595; 0.06595)	0.06594	(0.06593; 0.06594)	-0.0000065	(-0.0000149; -0.0000013)	-0.010	(-0.023; -0.002)
		0.1	0.25693	(0.25693; 0.25693)	0.25691	(0.25687; 0.25693)	-0.0000275	(-0.0000635; -0.0000061)	-0.011	(-0.025; -0.002)
		0.15	0.37914	(0.37914; 0.37914)	0.3791	(0.37905; 0.37913)	-0.0000421	(-0.0000972; -0.0000097)	-0.011	(-0.026; -0.003)
		0.3	0.72594	(0.72594; 0.72594)	0.72586	(0.72575; 0.72592)	-0.0000848	(-0.0001981; -0.0000203)	-0.012	(-0.027; -0.003)
QALE at $150k	Atkinson	0.9	0.00049	(0.00049; 0.00049)	0.00049	(0.00049; 0.00049)	-0.0000001	(-0.0000001; 0.0000000)	-0.012	(-0.027; -0.003)
		5	0.0027	(0.0027; 0.0027)	0.0027	(0.0027; 0.0027)	-0.0000004	(-0.0000008; -0.0000001)	-0.013	(-0.029; -0.004)
		11	0.0058	(0.0058; 0.0058)	0.0058	(0.0058; 0.0058)	-0.0000008	(-0.0000018; -0.0000002)	-0.014	(-0.03; -0.004)
		15	0.0078	(0.0078; 0.0078)	0.0078	(0.0078; 0.0078)	-0.0000011	(-0.0000024; -0.0000003)	-0.014	(-0.031; -0.004)
	Kolm	0.025	0.06595	(0.06595; 0.06595)	0.06594	(0.06593; 0.06594)	-0.0000065	(-0.0000149; -0.0000013)	-0.010	(-0.023; -0.002)
		0.1	0.25693	(0.25693; 0.25693)	0.25691	(0.25687; 0.25693)	-0.0000275	(-0.0000635; -0.0000061)	-0.011	(-0.025; -0.002)
		0.15	0.37914	(0.37914; 0.37914)	0.3791	(0.37905; 0.37913)	-0.0000421	(-0.0000972; -0.0000097)	-0.011	(-0.026; -0.003)
		0.3	0.72594	(0.72594; 0.72594)	0.72586	(0.72575; 0.72592)	-0.0000848	(-0.0001981; -0.0000203)	-0.012	(-0.027; -0.003)
QALE at $200k	Atkinson	0.9	0.00049	(0.00049; 0.00049)	0.00049	(0.00049; 0.00049)	-0.0000001	(-0.0000001; 0.0000000)	-0.013	(-0.027; -0.003)
		5	0.0027	(0.0027; 0.0027)	0.0027	(0.0027; 0.0027)	-0.0000004	(-0.0000008; -0.0000001)	-0.013	(-0.029; -0.004)
		11	0.0058	(0.0058; 0.0058)	0.0058	(0.0058; 0.0058)	-0.0000008	(-0.0000018; -0.0000002)	-0.014	(-0.030; -0.004)
		15	0.0078	(0.0078; 0.0078)	0.0078	(0.0078; 0.0078)	-0.0000011	(-0.0000024; -0.0000003)	-0.014	(-0.031; -0.004)
	Kolm	0.025	0.06595	(0.06595; 0.06595)	0.06594	(0.06593; 0.06594)	-0.0000065	(-0.0000149; -0.0000013)	-0.010	(-0.023; -0.002)

0.1	0.25693	(0.25693; 0.25693)	0.25691	(0.25687; 0.25693)	-0.0000275	(-0.0000635; -0.0000061)	-0.011	(-0.025; -0.002)
0.15	0.37914	(0.37914; 0.37914)	0.3791	(0.37905; 0.37913)	-0.0000421	(-0.0000972; -0.0000097)	-0.011	(-0.026; -0.003)
0.3	0.72594	(0.72594; 0.72594)	0.72586	(0.72575; 0.72592)	-0.0000848	(-0.0001981; -0.0000203)	-0.012	(-0.027; -0.003)

* difference: $Atkinson_{LB\text{-}first} - Atkinson_{TB\text{-}only}$ or $Kolm_{LB\text{-}first} - Kolm_{TB\text{-}only}$. A positive number implies an increase in inequality

** %difference: $100*(Atkinson_{LB\text{-}first} - Atkinson_{TB\text{-}only})/Atkinson_{TB\text{-}only}$ or $100*(Kolm_{LB\text{-}first} - Kolm_{TB\text{-}only})/Kolm_{TB\text{-}only}$. A positive number implies an increase in inequality

Table S9: Inequality metrics for QALE per individual of the general population factoring in equally distributed opportunity costs related to diagnostic workup, treatment, and disease management at threshold of $50k, $100k, $150k, $200k for different degrees of inequality aversion.

Outcome	Metric	Inequality aversion	Tissue biopsy only		Liquid biopsy first		Difference*		% Difference	
			mean	95% uncertainty interval	mean	95% uncertainty interval	mean	95% uncertainty interval	mean	95% uncertainty interval
QALE at $50k	Atkinson	0.9	0.00049	(0.00049; 0.00049)	0.00049	(0.00049; 0.00049)	0.0000000	(0.0000000; 0.0000001)	0.004	(-0.004; 0.013)
		5	0.0027	(0.0027; 0.0027)	0.0027	(0.0027; 0.0027)	0.0000001	(-0.0000001; 0.0000003)	0.003	(-0.005; 0.012)
		11	0.0058	(0.0058; 0.0058)	0.0058	(0.0058; 0.0058)	0.0000001	(-0.0000004; 0.0000007)	0.002	(-0.007; 0.012)
		15	0.0078	(0.0078; 0.0078)	0.0078	(0.0078; 0.0078)	0.0000001	(-0.0000006; 0.0000008)	0.002	(-0.008; 0.011)
	Kolm	0.025	0.06595	(0.06595; 0.06595)	0.06594	(0.06593; 0.06594)	-0.0000065	(-0.0000149; -0.0000013)	-0.010	(-0.023; -0.002)
		0.1	0.25693	(0.25693; 0.25693)	0.25691	(0.25687; 0.25693)	-0.0000275	(-0.0000635; -0.0000061)	-0.011	(-0.025; -0.002)
		0.15	0.37914	(0.37914; 0.37914)	0.37910	(0.37905; 0.37913)	-0.0000421	(-0.0000972; -0.0000097)	-0.011	(-0.026; -0.003)
		0.3	0.72594	(0.72594; 0.72594)	0.72586	(0.72575; 0.72592)	-0.0000848	(-0.0001981; -0.0000203)	-0.012	(-0.027; -0.003)
QALE at $100k	Atkinson	0.9	0.00049	(0.00049; 0.00049)	0.00049	(0.00049; 0.00049)	0.0000000	(-0.0000001; 0.0000000)	-0.005	(-0.015; 0.002)
		5	0.0027	(0.0027; 0.0027)	0.0027	(0.0027; 0.0027)	-0.0000001	(-0.0000005; 0.0000000)	-0.005	(-0.017; 0.002)
		11	0.0058	(0.0058; 0.0058)	0.0058	(0.0058; 0.0058)	-0.0000004	(-0.0000011; 0.0000001)	-0.006	(-0.018; 0.001)
		15	0.0078	(0.0078; 0.0078)	0.0078	(0.0078; 0.0078)	-0.0000005	(-0.0000015; 0.0000001)	-0.006	(-0.019; 0.001)

	Kolm	0.025	0.06595	(0.06595; 0.06595)	0.06594	(0.06593; 0.06594)	-0.0000065	(-0.0000149; -0.0000013)	-0.01	(-0.023; -0.002)
		0.1	0.25693	(0.25693; 0.25693)	0.25691	(0.25687; 0.25693)	-0.0000275	(-0.0000635; -0.0000061)	-0.011	(-0.025; -0.002)
		0.15	0.37914	(0.37914; 0.37914)	0.3791	(0.37905; 0.37913)	-0.0000421	(-0.0000972; -0.0000097)	-0.011	(-0.026; -0.003)
		0.3	0.72594	(0.72594; 0.72594)	0.72586	(0.72575; 0.72592)	-0.0000848	(-0.0001981; -0.0000203)	-0.012	(-0.027; -0.003)
QALE at $150k	Atkinson	0.9	0.00049	(0.00049; 0.00049)	0.00049	(0.00049; 0.00049)	0.0000000	(-0.0000001; 0.0000000)	-0.007	(-0.019; 0.000)
		5	0.0027	(0.0027; 0.0027)	0.0027	(0.0027; 0.0027)	-0.0000002	(-0.0000006; 0.0000000)	-0.008	(-0.02; -0.001)
		11	0.0058	(0.0058; 0.0058)	0.0058	(0.0058; 0.0058)	-0.0000005	(-0.0000013; -0.0000001)	-0.009	(-0.022; -0.001)
		15	0.0078	(0.0078; 0.0078)	0.0078	(0.0078; 0.0078)	-0.0000007	(-0.0000018; -0.0000001)	-0.009	(-0.023; -0.001)
	Kolm	0.025	0.06595	(0.06595; 0.06595)	0.06594	(0.06593; 0.06594)	-0.0000065	(-0.0000149; -0.0000013)	-0.01	(-0.023; -0.002)
		0.1	0.25693	(0.25693; 0.25693)	0.25691	(0.25687; 0.25693)	-0.0000275	(-0.0000635; -0.0000061)	-0.011	(-0.025; -0.002)
		0.15	0.37914	(0.37914; 0.37914)	0.3791	(0.37905; 0.37913)	-0.0000421	(-0.0000972; -0.0000097)	-0.011	(-0.026; -0.003)
		0.3	0.72594	(0.72594; 0.72594)	0.72586	(0.72575; 0.72592)	-0.0000848	(-0.0001981; -0.0000203)	-0.012	(-0.027; -0.003)
QALE at $200k	Atkinson	0.9	0.00049	(0.00049; 0.00049)	0.00049	(0.00049; 0.00049)	0.0000000	(-0.0000001; 0.0000000)	-0.009	(-0.021; -0.001)
		5	0.0027	(0.0027; 0.0027)	0.0027	(0.0027; 0.0027)	-0.0000003	(-0.0000006; 0.0000000)	-0.009	(-0.023; -0.002)
		11	0.0058	(0.0058; 0.0058)	0.0058	(0.0058; 0.0058)	-0.0000006	(-0.0000014; -0.0000001)	-0.01	(-0.024; -0.002)
		15	0.0078	(0.0078; 0.0078)	0.0078	(0.0078; 0.0078)	-0.0000008	(-0.000002; -0.0000002)	-0.01	(-0.025; -0.002)
	Kolm	0.025	0.06595	(0.06595; 0.06595)	0.06594	(0.06593; 0.06594)	-0.0000065	(-0.0000149; -0.0000013)	-0.01	(-0.023; -0.002)

	0.1	0.25693	(0.25693; 0.25693)	0.25691	(0.25687; 0.25693)	-0.0000275	(-0.0000635; -0.0000061)	-0.011	(-0.025; -0.002)
	0.15	0.37914	(0.37914; 0.37914)	0.3791	(0.37905; 0.37913)	-0.0000421	(-0.0000972; -0.0000097)	-0.011	(-0.026; -0.003)
	0.3	0.72594	(0.72594; 0.72594)	0.72586	(0.72575; 0.72592)	-0.0000848	(-0.0001981; -0.0000203)	-0.012	(-0.027; -0.003)

* difference: $Atkinson_{LB\text{-}first} – Atkinson_{TB\text{-}only}$ or $Kolm_{LB\text{-}first} – Kolm_{TB\text{-}only}$. A positive number implies an increase in inequality

** %difference: $100*(Atkinson_{LB\text{-}first} – Atkinson_{TB\text{-}only})/Atkinson_{TB\text{-}only}$ or $100*(Kolm_{LB\text{-}first} – Kolm_{TB\text{-}only})/Kolm_{TB\text{-}only}$. A positive number implies an increase in inequality

Table S10: Expected discounted QALYs per patient, discounted costs related to diagnostic workup per patient, iNHB per 100,000 individuals of the general population factoring in equally distributed opportunity costs at a threshold of $150k, and QALE per member of the general population by race and ethnicity without and with LB-first under the assumption of a 1-week shorter TAT with LB-first.

Outcome	Group	Tissue biopsy only		Liquid biopsy first		Difference	
		mean	95% uncertainty interval	mean	95% uncertainty interval	mean	95% uncertainty interval
QALYs (per patient)	NH-White	1.38	(1.11; 1.74)	1.45	(1.15; 1.82)	0.07	(0.02; 0.13)
	NH-Black	1.38	(1.09; 1.75)	1.45	(1.14; 1.87)	0.07	(0.02; 0.13)
	Asian	1.67	(1.37; 2.06)	1.77	(1.44; 2.18)	0.1	(0.02; 0.19)
	Hispanic	1.46	(1.18; 1.84)	1.52	(1.21; 1.9)	0.05	(0.02; 0.11)
	All	1.41	(1.13; 1.77)	1.47	(1.18; 1.86)	0.07	(0.02; 0.13)
Costs (US$, diagnostic workup, per patient)	NH-White	4072	(4016; 4127)	7398	(7064; 7731)		
	NH-Black	4072	(4016; 4127)	7267	(6847; 7698)		
	Asian	4072	(4016; 4127)	6608	(6099; 7125)		
	Hispanic	4072	(4016; 4127)	7503	(7041; 7963)		
	All	4072	(4016; 4127)	7342	(7011; 7699)	3270	(2934; 3633)
Incremental NHB (in QALYs per 100,000 individuals of the general population)	NH-White					25	(1; 57)
	NH-Black					30	(2; 66)
	Asian					33	(1; 76)
	Hispanic					4	(-7; 21)
	All					22	(0; 52)
		Pre LB-first distribution		*Post LB-first distribution*			
QALE (per individual general population)	NH-White	68.798		68.798	(68.798; 68.799)		
	NH-Black	65.446		65.447	(65.446; 65.447)		
	Asian	74.878		74.879	(74.878; 74.879)		
	Hispanic	71.762		71.762	(71.762; 71.762)		
	All	69.283		69.284	(69.283; 69.284)		

Table S11: Inequality metrics for expected QALYs per patient and expected QALE per individual of the general population factoring in equally distributed opportunity costs related to diagnostic workup at a threshold of $150k and different degrees of inequality aversion under the assumption of a 1-week shorter TAT with LB-first.

Outcome	Metric	Inequality aversion	Tissue biopsy only		Liquid biopsy first		Difference*	
			mean	95% uncertainty interval	mean	95% uncertainty interval	mean	95% uncertainty interval
QALYs (per patient)	Atkinson	0.9	0.00134	(0.00048; 0.00256)	0.0014	(0.00052; 0.0027)	0.00006	(-0.00012; 0.00032)
		5	0.00627	(0.00234; 0.01184)	0.00646	(0.0025; 0.01243)	0.0002	(-0.00059; 0.00120)
		11 (base-case)	0.01109	(0.00439; 0.02149)	0.01126	(0.00454; 0.02205)	0.00018	(-0.00116; 0.00192)
		15	0.01337	(0.00535; 0.02654)	0.01349	(0.00554; 0.02646)	0.00012	(-0.00143; 0.00219)
	Kolm	0.025	0.00008	(0.00003; 0.00015)	0.00009	(0.00004; 0.00017)	0.00001	(0.0000000; 0.00003)
		0.1	0.00031	(0.00013; 0.00061)	0.00036	(0.00015; 0.00068)	0.00005	(-0.00001; 0.00014)
		0.15 (base-case)	0.00046	(0.00020; 0.00091)	0.00054	(0.00023; 0.00102)	0.00007	(-0.00001; 0.00020)
		0.3	0.00092	(0.00039; 0.00180)	0.00106	(0.00045; 0.00201)	0.00014	(-0.00002; 0.00039)
QALE (per individual general population)	Atkinson	0.9	0.0004948	(0.0004948; 0.0004948)	0.0004948	(0.0004948; 0.0004948)	0.0000000	(0.0000000; 0.0000000)
		5	0.0027011	(0.0027011; 0.0027011)	0.002701	(0.0027009; 0.0027011)	-0.0000001	(-0.0000003; 0.0000000)
		11 (base-case)	0.0058032	(0.0058032; 0.0058032)	0.005803	(0.0058027; 0.0058032)	-0.0000003	(-0.0000006; -0.0000001)
		15	0.0077995	(0.0077995; 0.0077995)	0.0077991	(0.0077987; 0.0077994)	-0.0000004	(-0.0000008; -0.0000001)

Outcome	Metric	Inequality aversion	Tissue biopsy only		Liquid biopsy first		Difference*	
			mean	95% uncertainty interval	mean	95% uncertainty interval	mean	95% uncertainty interval
	Kolm	0.025	0.065946	(0.065946; 0.065946)	0.0659436	(0.0659408; 0.0659455)	-0.0000024	(-0.0000052; -0.0000006)
		0.1	0.2569327	(0.2569327; 0.2569327)	0.2569227	(0.2569119; 0.2569303)	-0.0000099	(-0.0000208; -0.0000024)
		0.15 (base-case)	0.3791431	(0.3791431; 0.3791431)	0.379128	(0.3791116; 0.3791391)	-0.0000151	(-0.0000315; -0.0000039)
		0.3	0.7259447	(0.7259447; 0.7259447)	0.725915	(0.7258813; 0.7259371)	-0.0000297	(-0.0000634; -0.0000076)

* difference: $Atkinson_{LB\text{-}first} - Atkinson_{TB\text{-}only}$ or $Kolm_{LB\text{-}first} - Kolm_{TB\text{-}only}$. A positive number implies an increase in inequality
